# Induction of DEPP1 by HIF Mediates Multiple Hallmarks of Ischemic Cardiomyopathy

**DOI:** 10.1161/CIRCULATIONAHA.123.066628

**Published:** 2024-06-17

**Authors:** Gregory A. Wyant, Qinqin Jiang, Madhu Singh, Shariq Qayyum, Clara Levrero, Bradley A. Maron, William G. Kaelin

**Affiliations:** Department of Medical Oncology, Dana-Farber Cancer Institute, Boston, MA (G.A.W., Q.J., C.L., W.G.K.).; Department of Cardiovascular Medicine (B.A.M.), Brigham and Women’s Hospital, Harvard Medical School, Boston, MA.; Department of Medicine (W.G.K.), Brigham and Women’s Hospital, Harvard Medical School, Boston, MA.; Cardiovascular Research Center, Cardiology Division, Department of Medicine, Massachusetts General Hospital and Harvard Medical School, Boston, MA (G.A.W., M.S., S.Q.).; Howard Hughes Medical Institute, Chevy Chase, MD (W.G.K.).

**Keywords:** autophagy, cardiomyocyte, DEPP1, HIF, hypoxia, mitochondria, peroxisome, VHL

## Abstract

**BACKGROUND:**

HIF (hypoxia inducible factor) regulates many aspects of cardiac function. We and others previously showed that chronic HIF activation in the heart in mouse models phenocopies multiple features of ischemic cardiomyopathy in humans, including mitochondrial loss, lipid accumulation, and systolic cardiac dysfunction. In some settings, HIF also causes the loss of peroxisomes. How, mechanistically, HIF promotes cardiac dysfunction is an open question.

**METHODS:**

We used mice lacking cardiac pVHL (von Hippel-Lindau protein) to investigate how chronic HIF activation causes multiple features of ischemic cardiomyopathy, such as autophagy induction and lipid accumulation. We performed immunoblot assays, RNA sequencing, mitochondrial and peroxisomal autophagy flux measurements, and live cell imaging on isolated cardiomyocytes. We used CRISPR-Cas9 gene editing in mice to validate a novel mediator of cardiac dysfunction in the setting of chronic HIF activation.

**RESULTS:**

We identify a previously unknown pathway by which cardiac HIF activation promotes the loss of mitochondria and peroxisomes. We found that DEPP1 (decidual protein induced by progesterone 1) is induced under hypoxia in a HIF-dependent manner and localizes inside mitochondria. DEPP1 is both necessary and sufficient for hypoxia-induced autophagy and triglyceride accumulation in cardiomyocytes ex vivo. DEPP1 loss increases cardiomyocyte survival in the setting of chronic HIF activation ex vivo, and whole-body Depp1 loss decreases cardiac dysfunction in hearts with chronic HIF activation caused by *VHL* loss in vivo.

**CONCLUSIONS:**

Our findings identify DEPP1 as a key component in the cardiac remodeling that occurs with chronic ischemia.

**Clinical Perspective**

**What Is New?**

- We investigated how chronic HIF (hypoxia inducible factor) activation leads to many features of ischemic cardiomyopathy and identified a previously unknown pathway by which cardiac HIF activation promotes the loss of mitochondria and peroxisomes through autophagy.
- We discovered that DEPP1 (decidual protein induced by progesterone 1) is a key component in the cardiac remodeling that occurs with chronic ischemia and that DEPP1 loss decreases cardiac dysfunction in the setting of chronic HIF activation.

**What Are the Clinical Implications?**

- Our findings provide mechanistic insights that underlie key molecular changes that occur in the ischemic myocardium.
- Strategies that increase mitochondrial and peroxisome abundance, such as by inhibiting DEPP1, might help preserve cardiac function in the setting of chronic ischemia.

**Editorial, see p 787**

Chronic HIF (hypoxia inducible factor) activation in mouse models is sufficient to produce many of the hallmarks of ischemic cardiomyopathy.^1–3^ For example, HIF causes the loss of cardiac mitochondria, lipid accumulation, and systolic cardiac dysfunction, which are features of human ischemic cardiomyopathy.^1–3^ How HIF induces these changes is poorly understood.

The HIF transcription factor, which consists of a labile α subunit (HIFα) and a stable β subunit (HIFβ/ARNT [aryl hydrocarbon nuclear translocator]), accumulates during hypoxia and activates genes that orchestrate the cellular adaptation to hypoxia.^4^ In the presence of oxygen, HIFα becomes hydroxylated on 1 (or both) of 2 conserved proline residues by members of the PHD1-3 (prolyl hydroxylase domain), also known as the EglN (Egl9 homolog), family. PHD enzymatic activity requires oxygen, reduced iron, and 2-oxoglutarate and is sensitive to other inputs that indirectly reflect oxygen availability, such as reactive oxygen species (ROS) and Krebs cycle intermediates. Thus, the PHD proteins act as oxygen sensors upstream of the HIFα transcription factor. Once hydroxylated, HIFα is polyubiquitinylated by an E3 ubiquitin ligase complex containing pVHL (von Hippel-Lindau protein), leading to its proteasomal degradation.^4^

Systematic inactivation of PHD2, especially when combined with PHD3 loss, leads to dilated cardiomyopathy, suggesting that impaired PHD activity, as would occur in the setting of chronic ischemia, causes cardiac dysfunction.^5,6^ Although these studies were potentially confounded by the polycythemia and volume overload that occurs with systemic PHD2 loss, cardiac-specific *VHL* deletion in mice is also sufficient to promote cardiomyopathy, which is completely prevented by concomitant deletion of HIF1α.^2^ Moreover, we and others showed that cardiomyocyte-intrinsic HIF activation is sufficient to account for many of the electron microscopic, histological, and functional changes in hearts with ischemic cardiomyopathy.^1–3^ Collectively these findings suggest that chronic HIF activation in cardiomyocytes plays a causal role in the pathogenesis of ischemic cardiomyopathy.

How chronic HIF activation promotes heart failure, however, remains unclear. Chronic HIF activation leads to decreased mitochondrial biogenesis, increased autophagy, increased lipid accumulation, and subsequent metabolic dysfunction.^1,7,8^ HIF can selectively induce mitochondria and peroxisome autophagy, 2 organelles that play major roles in oxygen, redox, energy, and lipid metabolism.^9^ BNIP3 (Bcl-2/adenovirus E1B 19-kDa interacting protein 3), a canonical HIF target gene, plays a role in hypoxia-induced mitochondrial autophagy and is increased in mouse hearts lacking PHD or pVHL function.^10^ *BNIP3* loss minimizes ventricular remodeling and cardiomyocyte apoptosis after acute ischemic reperfusion injury, which suggests that inhibiting autophagy would be therapeutically useful in this acute setting.^11^ Whether increased autophagy is an adaptive or deleterious response to chronic cardiac ischemia and whether other specific HIF-responsive proteins are necessary for HIF to promote autophagy in the heart remains to be determined. Whether autophagy inhibition would alter the natural history of heart failure in the setting of chronic HIF activation is therefore an open question.

Here, we show that hypoxia and chronic HIF activation promotes mitochondrial and peroxisome autophagy and lipid accumulation in cardiomyocytes ex vivo and in vivo. We identify DEPP1 (decidual protein induced by progesterone 1) as a direct transcriptional target of HIF that is necessary for mitochondrial and peroxisome autophagy mediated by HIF activation, but not by other autophagy inducers, such as mammalian Target of Rapamycin (mTOR) inhibition. Upon expression, DEPP1 localizes inside mitochondria and is sufficient to promote cardiomyocyte autophagy and lipid accumulation. *DEPP1* loss increases cardiomyocyte survival in the setting chronic HIF activation, and global *Depp1* loss decreases cardiac dysfunction in mouse hearts lacking *VHL*. These studies provide new insights into how HIF activation promotes autophagy and suggest that inhibiting autophagy in the setting of chronic ischemia would prevent the loss of mitochondria and peroxisomes and preserve cardiac function.

## METHODS

Detailed methods are provided in the Supplemental Material. All data needed to evaluate the conclusions in the article are present in the article or the Supplemental Material. All plasmids described herein are available from the authors on request.

### Experimental Animals

All care and treatment of experimental animals were carried out in strict accordance with good animal practice as defined by the US Office of Laboratory Animal Welfare and approved by the Dana-Farber Cancer Institute (protocols 04-019 and 04-020) institutional animal care and use committee.

### Human Heart Biopsy Samples

Hearts were obtained under a protocol approved by the University of Utah institutional review board.

### Statistical Analysis

GraphPad Prism versions 9 and 10 were used for statistical analysis included in the main and supplemental figures. Statistical significance was calculated using the unpaired, 2-tailed Student *t* test or 1-way ANOVA with Šidák multiple comparisons test for post hoc pairwise comparisons. When 2 independent factors were used in the experimental design, statistical significance was calculated using a 2-way ANOVA with Šidák multiple comparisons test. Data were evaluated for normality before parametric statistics were applied using the Shapiro-Wilk test. For groups of unequal variances, data were reevaluated by *t* test or ANOVA with Welch correction. For comparisons for 2 groups with nonnormal distribution, the Mann-Whitney test was used. For comparisons of >2 groups with nonnormal distribution, the Kruskal-Wallis test was used. Survival curve analysis was performed using the log-rank test. *P* values were considered statistically significant if the *P* value was <0.05. For all figures, an asterisk indicates *P < 0.05 unless otherwise indicated. Error bars represent SD unless otherwise indicated. No statistical methods were used to predetermine the sample size.

## RESULTS

### Chronic HIF Activation Caused by Cardiac VHL Loss or Hypoxia Promotes Autophagy-Dependent Mitochondria and Peroxisome Loss

**Figure 1.**
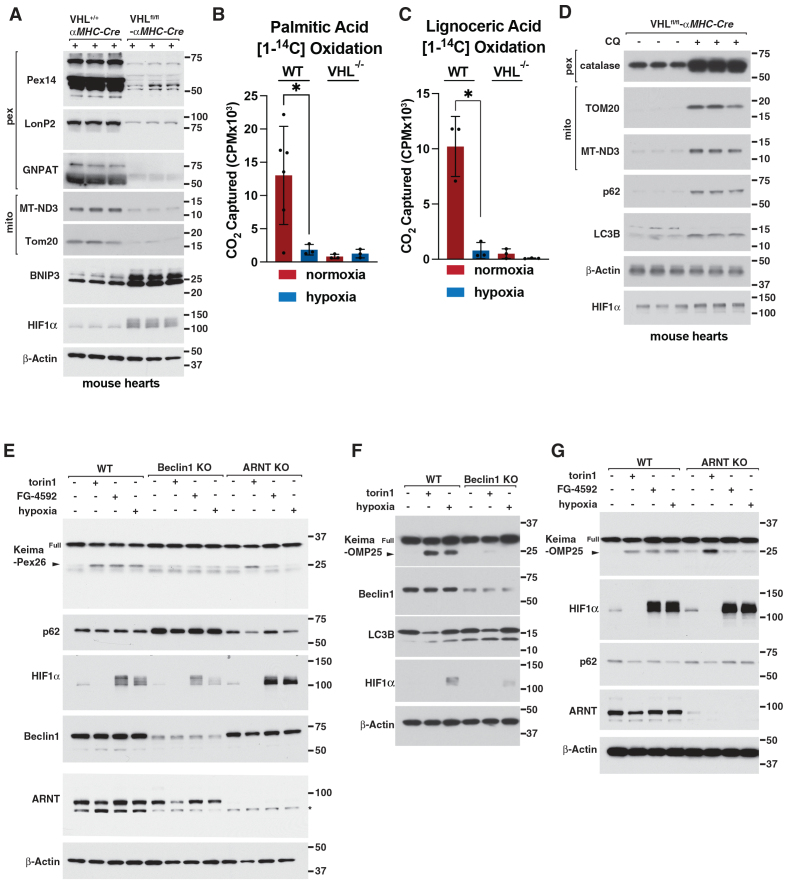
**Hypoxia or VHL loss induces autophagy-dependent mitochondrial and peroxisomal loss. A**, Immunoblot analysis of heart lysates from mice with the indicated genotypes (n=3 per genotype, male 5 weeks of age). **B** and **C**, ^14^C-palmitic acid (**B**) or lignoceric acid (**C**) oxidation assay in wild-type or *VHL* knockout mouse neonatal cardiomyocytes grown under hypoxia (1% O_2_) or normoxia for 18 hours. Mean±SD. n>3, *P<0.05 by 2-way ANOVA with Šidák multiple comparisons test. **D**, Immunoblot analysis of mouse *VHL*^-/-^*-αMHC-Cre* heart lysates (n=3 per genotype, male 5 weeks of age). Where indicated, the mice were treated with 60 mg/kg chloroquine (CQ) for 7 days. **E**, Immunoblot analysis of wild-type, *Beclin1* knockout, or *ARNT* knockout AC16 human cardiomyocytes stably expressing Keima-Pex26. Where indicated, cells were treated with 250 nM Torin1, 100 μM FG-4592, or hypoxia (1% O_2_) for 18 hours. Arrow indicates liberated Keima protein. *Nonspecific band. **F** and **G**, Immunoblot analysis of wild-type, *Beclin1* knockout, or *ARNT* knockout AC16 cardiomyocytes stably expressing Keima-OMP25. Where indicated, cells were treated with 250 nM Torin1, 100 µM FG-4592, or hypoxia (1% O_2_) for 18 hours. Arrow indicates liberated Keima protein.

To understand how chronic HIF activation promotes the development of cardiomyopathy, we inactivated the *VHL* gene in the mouse heart. *VHL*^fl/fl^ and *VHL*^+/+^ mice were crossed with mice expressing Cre recombinase in cardiomyocytes under the control of the myosin heavy chain promoter (αMHC-Cre). As expected, cardiac *VHL* loss increased the accumulation of HIF and proteins encoded by well-studied HIF-responsive mRNAs, such as BNIP3, in mouse hearts, but not in skeletal muscle, as determined by immunoblot analysis (Figure 1A; Figure S1A).

We previously showed that mice lacking *VHL* in the heart succumb to heart failure in ≈10 weeks and display dysregulated metabolism, mitochondrial abnormalities, and increased autophagy.^1^ Previous studies showed that HIF promotes mitochondrial and peroxisomal autophagy, at least in some settings, presumably to maintain O_2_ homeostasis under hypoxia.^9^ Mitochondria and peroxisomes play essential roles in redox, lipid, and energy metabolism.^12^ Whether the loss of these 2 organelles causes, rather than merely correlates with, heart failure in the setting of chronic ischemia has not been determined. Cardiac *VHL* loss decreased peroxisomal and mitochondrial protein abundance, peroxisomal number and size, and mitochondrial area in whole tissue lysates and isolated mouse cardiomyocytes compared with wild-type counterparts (Figure 1A; Figure S1B through S1F). Similarly, hypoxia and *VHL* loss decreased mitochondrial and peroxisomal function as measured by mitochondrial oxygen consumption through Seahorse analysis and oxidation of the mitochondria-specific substrate palmitic acid and the peroxisome-specific substrate lignoceric acid, respectively (Figure 1B and 1C; Figure S1G).

We asked whether *VHL* loss decreased cardiac mitochondria and peroxisome abundance through autophagy-dependent degradation. We confirmed cardiomyocytes isolated from *VHL*^fl/fl^; αMHC-Cre hearts or *VHL* knockout cardiomyocytes generated using CRISPR-Cas9 displayed increased autophagy as measured by increased microtubule-associated proteins 1A/1B light chain 3B (LC3B), WD repeat domain phosphoinositide-interacting protein 1 (WIPI-I), and Sequestosome-1 (SQSTM1/p62) puncta compared with wild-type counterparts by confocal microscopy (Figure S2A through S2C). Similarly, hypoxia promoted mitochondria and peroxisome colocalization with LAMP1 (Lysosome-associated membrane glycoprotein 1)-positive lysosomes, the final destination of autophagy cargo, in fixed cardiomyocytes by confocal microscopy (Figure S3A through S3B). In addition, hypoxia promoted biotin labeling of LAMP1, as measured by confocal microscopy using streptavidin-568 as a probe, in cardiomyocytes expressing PEX26 (Peroxisome assembly protein 26), a resident peroxisome membrane protein, fused to a biotin ligase (APEX [ascorbate peroxidase]) (Figure S4A and S4B). These results were autophagy-dependent because they were abrogated by loss of *Beclin1*, a core autophagy component, through CRISPR-Cas9 (Figure S3A and S3B; Figure S4B). To test whether autophagy inhibition could rescue mitochondria or peroxisome abundance in *VHL*^fl/fl^; αMHC-Cre hearts, we treated *VHL*^fl/fl^; αMHC-Cre mice with chloroquine, a pharmacologic inhibitor of lysosomal degradation. Chloroquine treatment of *VHL*^fl/fl^; αMHC-Cre mice increased mitochondrial and peroxisomal protein abundance in heart lysates (Figure 1D).

To facilitate the study of organelle trafficking during autophagy, we used Keima fusion proteins.^13^ Keima provides 2 important advantages. First, Keima itself is stable to lysosomal proteases. Accordingly, when a Keima fusion enters the lysosome, it liberates “processed” Keima (Keima and any peptidic remnants left behind after proteolysis of the fusion partner), which can be readily detected by immunoblot analysis. Second, Keima is also a pH-responsive reporter that undergoes a chromophore resting charge state change upon trafficking to the lysosome (pH ≈4.5). This enables autophagy flux measurements by quantifying the ratio of 561 nm/442 nm excitation by flow cytometry or confocal microscopy. We generated 2 different peroxisome-Keima reporters: one in which Keima was fused to the N terminus of amino acids 237–305 of human PEX26, which is sufficient for localization to and insertion into the peroxisomal membrane, and the other in which Keima was fused to the N terminus of full-length human PEX11A (Peroxisomal membrane protein 11A).^14,15^ In tandem, we generated a mitochondrially targeted Keima by fusing Keima to the N terminus of the well-characterized, outer mitochondrial membrane localization sequence of OMP25 (Keima-OMP25).^16^ We validated that the PEX26 and OMP25 sequences were sufficient to localize to PEX14 (Peroxisomal membrane protein PEX14)-mCherry peroxisomes or MitoTracker DeepRed–positive mitochondria, respectively, when fused to a fluorescent protein (Figure S5A and S5B).

We stably infected human AC16 cardiomyocytes to express either of the 2 Keima-peroxisome reporters (Keima-PEX26 or Keima-PEX11A) or the Keima-OMP25 reporter. Conditions that activate the HIF pathway such as hypoxia, *VHL* loss, and pharmacologic EglN inhibition with FG-4592 promoted Keima-PEX processing by immunoblotting (Figure 1E; Figure S5C through S5E). This processing was inhibited by SAR405, a VPS34 inhibitor that blocks the autophagy system, CRISPR-Cas9–mediated *Beclin1* loss, and chloroquine (Figure 1E; Figure S5C through S5E).^17^ As expected, other well-studied autophagy inducers, such as mTOR inhibition with Torin1 or amino acid starvation, also promoted Keima-PEX processing in a phosphatidylinositol 3-kinase, catalytic subunit type 3 (VPS34)- and Beclin1- dependent manner, consistent with autophagy-mediated peroxisome degradation (Figure 1E; Figure S5C and S5D). Similarly, hypoxia, FG-4592, Torin1, and mitophagy activators such as carbonyl cyanide p-trifluoro-methoxyphenyl hydrazone (FCCP) and oligomycin promoted VPS34- and Beclin1-dependent Keima-OMP25 processing by immunoblotting, consistent with autophagy-mediated mitochondrial degradation under these conditions (Figure 1F; Figure S5F).

To ask whether the HIF pathway specifically regulates the autophagy-mediated degradation of mitochondria and peroxisomes in cardiomyocytes, we inactivated *ARNT* using CRISPR-Cas9 in our Keima-PEX or OMP25 reporter cardiomyocytes to block HIF-mediated transcription. *ARNT* loss completely abrogated Keima-PEX or -OMP25 processing under hypoxia or FG-4592, as measured by immunoblotting. In contrast, *ARNT* loss did not abrogate Keima-PEX or -OMP25 processing induced by Torin1 (Figure 1E and 1G). Therefore, HIF is selectively required for the autophagy-dependent degradation of mitochondria and peroxisomes under hypoxia, rather than being generally required for autophagy.

### DEPP1 Is a Direct Transcriptional Target of HIF

**Figure 2.**
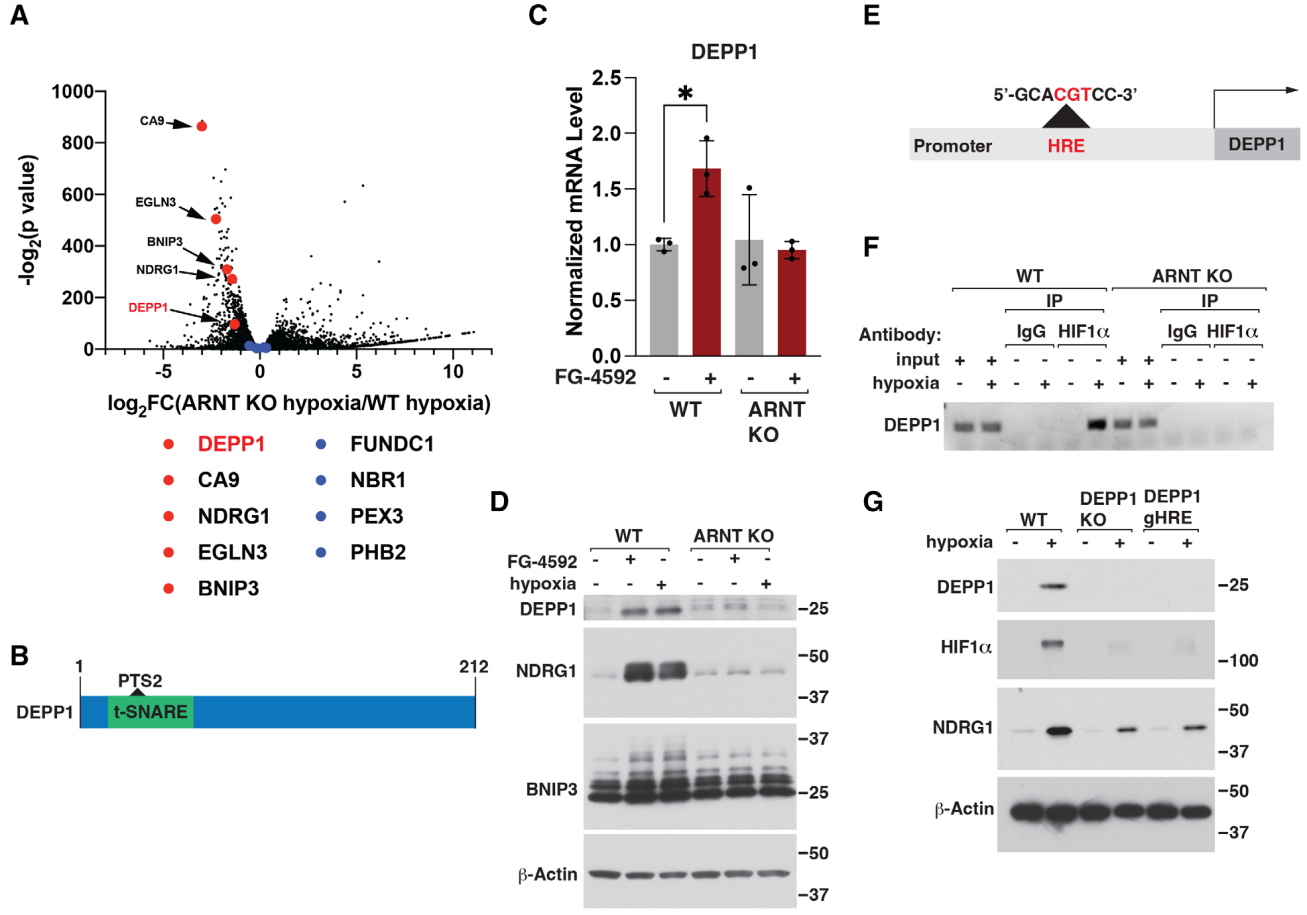
**DEPP1 is a direct HIFα target gene. A**, RNA sequencing analysis of wild-type or *ARNT* knockout human induced pluripotent stem cell–derived cardiomyocytes grown under hypoxia (1% O_2_) or normoxia for 18 hours. Fold change (FC). **B**, Schematic of DEPP1 protein domains. DEPP1 contains a peroxisomal targeting sequence type 2 (PTS2) within an N-terminal t-snare domain. **C**, *DEPP1* mRNA levels, as determined by real-time polymerase chain reaction, in wild-type or *ARNT* knockout cells treated with 100 µM FG-4592 or DMSO for 18 hours. Data show mean fold change ±SD, n=3, *P<0.05 by 2-way ANOVA with Šidák multiple comparisons test. **D**, Immunoblot analysis of wild-type or *ARNT* knockout U2OS cells treated, where indicated, with 100 µM FG-4592 or hypoxia (1% O_2_) for 18 hours. **E**, Schematic of *DEPP1* hypoxia response element (HRE). **F**, HIF1α chromatin immunoprecipitation (ChIP) in wild-type or *ARNT* knockout AC16 human cardiomyocytes grown at hypoxia (1% O_2_) or normoxia for 18 hours. IgG was used as a control antibody. **G**, Immunoblot analysis of wild-type, *DEPP1* knockout, or CRISPR-Cas9 editing of *DEPP1* HRE (DEPP1 gHRE) human IPS-derived cardiomyocytes cells grown under hypoxia (1% O_2_) or normoxia for 18 hours.

Our results with *ARNT* knockout cardiomyocytes suggested that 1 or more HIF-responsive gene products are necessary to promote mitochondrial and peroxisomal autophagy under conditions that activate the HIF pathway. To identify the responsible factor, we performed RNA sequencing in wild-type and *ARNT* knockout human induced pluripotent stem (hIPS) cell–derived cardiomyocytes (hIPS-CMs) grown under normoxic or hypoxia conditions (Figure 2A). As expected, hypoxia significantly increased the mRNA abundance of established HIF target genes, such as *CA9*, *EGLN3*, *BNIP3*, and *NDRG1*, in an ARNT-dependent manner. Hypoxia did not alter (positively or negatively) mRNA abundance of known mitochondrial or peroxisome autophagy factors, such as *NBR1*, *PEX3*, *PHB2*, or *FUNDC1* (Figure 2A).^18–21^

To narrow our search for candidate factor(s), we hypothesized that the mediator(s) of HIF-regulated mitochondrial and peroxisomal autophagy would have a mitochondrial or peroxisomal targeting sequence. Similarly, because several proteins have been identified that regulate both mitochondrial and peroxisomal compartments, we reasoned HIF would regulate autophagy of both organelles through a shared mechanism. Among the hypoxia-inducible, ARNT-dependent genes was *DEPP1* (Decidual protein induced by progesterone 1). DEPP1 was of interest because it contains a conserved PTS2 (peroxisomal targeting sequence type 2) nested within an N-terminal t-snare domain (Figure 2B). Moreover, DEPP1 has been linked with autophagy before through the nutrient sensitive FOXO (Forkhead box O) 3 transcription factor.^22–24^ We confirmed that *DEPP1* mRNA and protein abundance were induced by hypoxia and FG-4592 in an ARNT-dependent manner by real-time polymerase chain reaction and immunoblot assays in hIPS-CMs (Figure 2C) and U2OS cells (Figure 2D). *DEPP1* contains a conserved hypoxia response element (HRE) upstream of the *DEPP1* transcriptional start site (Figure 2E).^25^ We confirmed that HIF1α bound the *DEPP1* HRE during hypoxia in an ARNT-dependent manner using chromatin immunoprecipitation assays in human AC16 cardiomyocytes (Figure 2F). CRISPR-Cas9 *DEPP1* HRE editing, like CRISPR-Cas9 targeting of the *DEPP1* open reading frame, abrogated hypoxia-induced DEPP1 protein abundance in hIPS-CMs (Figure 2G). *DEPP1* loss also damped the induction of HIF1α and the product of HIF-responsive mRNAs, such as NDRG1, under hypoxia, suggesting that the HIF and DEPP1 participate in a positive feedback loop.

### DEPP1 Is Localized Inside Mitochondria

**Figure 3.**
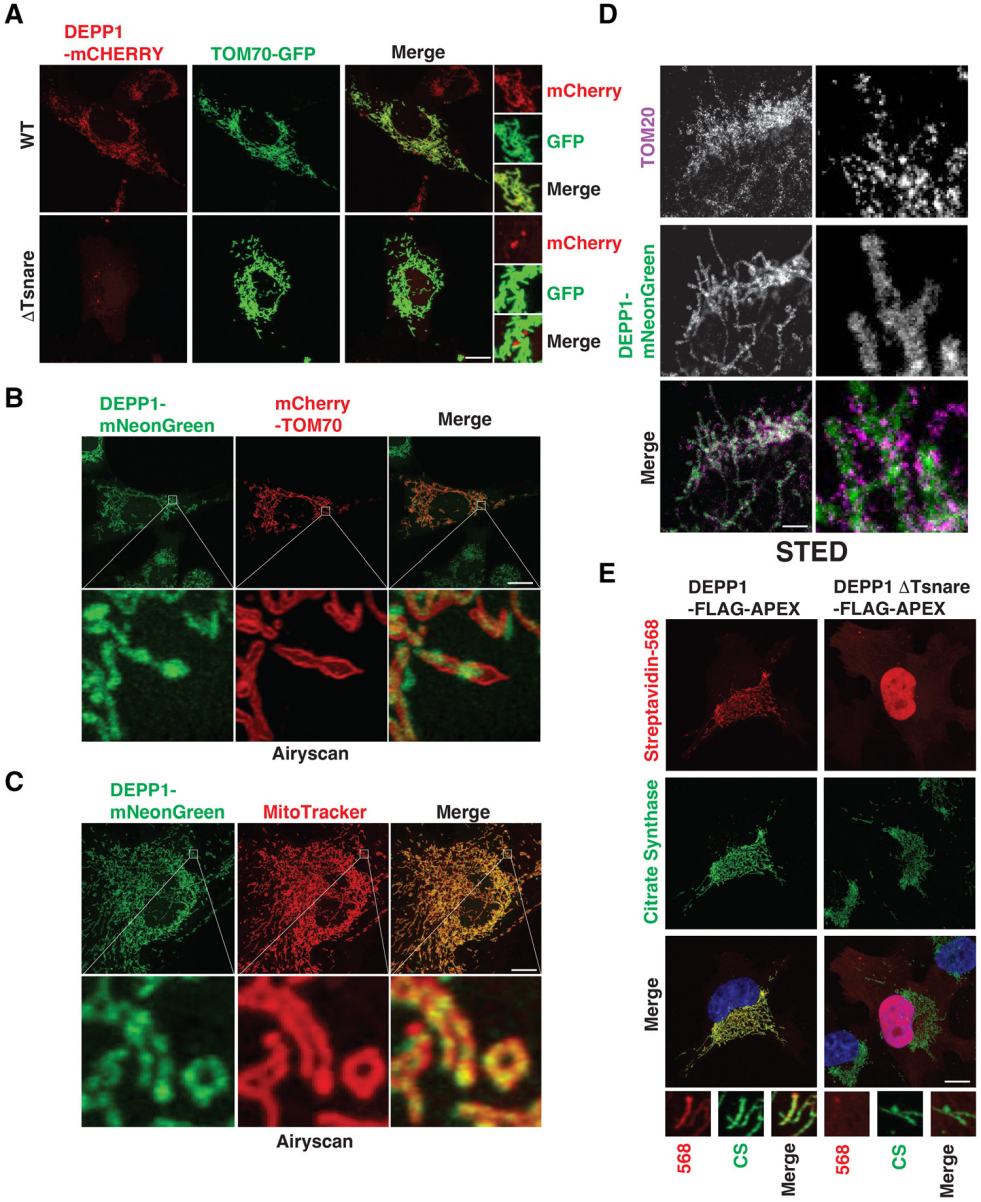
**DEPP1 localizes inside mitochondria. A**, Live cell confocal microscopy of *DEPP1* knockout AC16 human cardiomyocytes stably expressing full-length (WT) or ΔTsnare DEPP1-mCherry and TOM70-GFP. Scale bar=10 µm. **B** and **C**, Live cell confocal microscopy with Airyscan of *DEPP1* knockout AC16 human cardiomyocytes stably expressing DEPP1-mNeonGreen and mCherry-TOM70 or treated with MitoTracker. Scale bar, 10 µm. **D**, Super resolution microscopy (STED) of mouse neonatal cardiomyocytes stably expressing DEPP1-mNeonGreen. Scale bar=1 µm. **E**, Confocal microscopy of *DEPP1* knockout U2OS cells stably expressing wild-type or ΔTsnare DEPP1-Flag-APEX. Cells were treated with biotin-tryamide for 30 minutes followed by H_2_O_2_ for 1 minute (to enable biotinylation) and visualized with streptavidin-568. Scale bar=10 µm.

Previous overexpression studies showed that DEPP1 localizes to peroxisomes.^24^ To study this further, we made fluorescent versions of DEPP1 and a DEPP1 variant that lacks the N-terminal t-snare domain (ΔTsnare) by fusing them to either mCherry or mNeonGreen. These proteins were then introduced into *DEPP1* knockout cardiomyocytes by lentiviral infection followed by fluorescence cell sorting for cells in which the levels of the exogenous proteins approximated endogenous DEPP1 levels. We found, unexpectedly, that wild-type DEPP1 localized to mitochondria rather than peroxisomes in cardiomyocytes by confocal microscopy irrespective of the fluorophore (Figure 3A through 3C). Mitochondrial localization was confirmed using TOM70 (Mitochondrial import receptor subunit TOM70)-GFP (green fluorescent protein) and MitoTracker, 2 markers of mitochondria. In contrast, the ΔTsnare variant gave rise to diffuse cytosolic staining.

DEPP1-mCherry did not overlap with peroxisomes marked by GFP fused to PTS1 (GFP-PTS1) (Figure S6A). DEPP1-mCherry–positive mitochondria did, however, associate with GFP-PTS1, consistent with direct mitochondrial-peroxisome contacts between these organelles (Figure S6A).

We next tested whether DEPP1 is present inside or outside of mitochondria. Confocal microscopy with Airyscan revealed that DEPP1 resides in a diffuse pattern that completely overlaps with MitoTracker encased within the TOM70 outer mitochondrial membrane (Figure 3B and 3C; Figure S6B). Stimulated emission depletion super-resolution microscopy imaging, which provides an optical resolution superior to diffraction-limited confocal microscopy, revealed a similar diffuse DEPP1 pattern, whereas outer mitochondrial membrane protein TOM20 (Mitochondrial import receptor subunit TOM20) was distributed in a punctated pattern throughout the outer mitochondrial membrane (Figure 3D; Figure S6C). To ask whether DEPP1 resides within the mitochondrial inner membrane space or within the mitochondrial matrix, we fused DEPP1 or DEPP1 ΔTsnare to a biotin ligase (APEX) and measured biotinylation by confocal microscopy using streptavidin-568 as a probe. A mitochondrial matrix–localized APEX-generated biotin-phenoxyl radical will generate a restricted biotin-labeling pattern because the matrix is surrounded by the impermeable inner mitochondrial membrane. Conversely, an inner membrane space–localized APEX-generated biotin-phenoxyl radical will generate a diffuse biotinylation pattern because the outer mitochondrial matrix is porous to small molecules, including biotin-phenoxyl radicals.^26^ We introduced DEPP1-APEX or DEPP1ΔTsnare-APEX into DEPP1 knockout U2OS cells by lentiviral infection. We performed single-cell sorting to identify clones in which the exogenous DEPP1 levels approximated endogenous levels. DEPP1-APEX, in a t-snare–dependent manner, caused a restricted biotin-labeling pattern that completely overlapped with citrate synthase, a mitochondrial matrix enzyme (Figure 3E; Figure S6D). PMP70 (peroxisomal membrane protein 70)–positive peroxisomes associated with DEPP1-mediated streptavidin-568 consistent with direct mitochondrial-peroxisome contacts (Figure S6E). Therefore, DEPP1 localizes inside mitochondria, likely within the matrix compartment.

### DEPP1 Is Necessary for HIF-Mediated Autophagy and Triglyceride Accumulation

**Figure 4.**
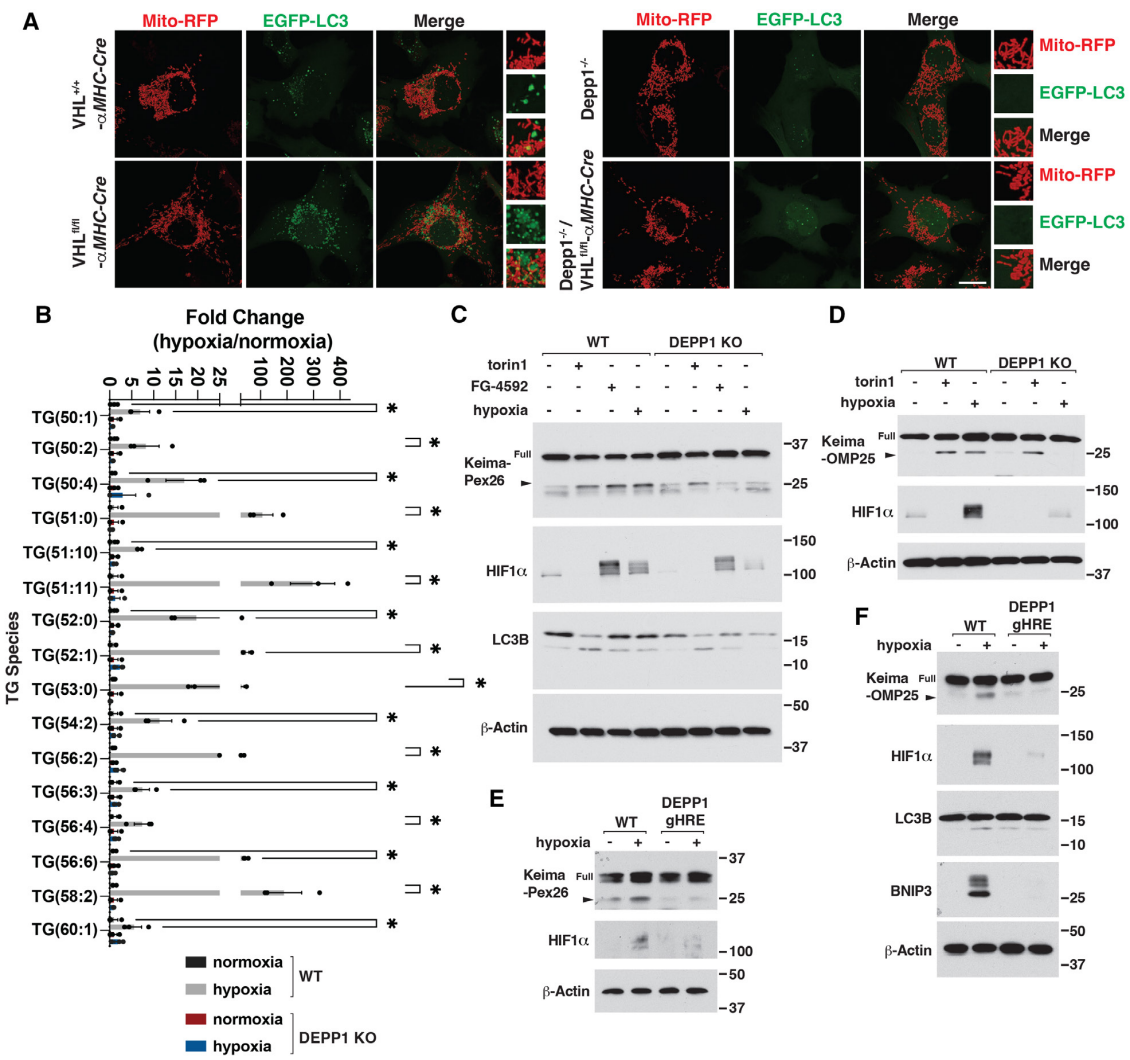
**DEPP1 is necessary for HIFα-mediated autophagy and triglyceride accumulation. A**, Live cell confocal microscopy of *VHL*^*+/+*^*-αMHC-Cre, VHL*^*-/*-^*-αMHC-Cre, Depp1*^*-/*-^*-αMHC-Cre*, or *Depp1*^*-/*-^*/VHL*^*-/*-^*-αMHC-Cre* mouse neonatal cardiomyocytes stably expressing Mito-RFP and EGFP-LC3. Scale bar=10 µm. **B**, Triglyceride (TG) analysis from wild-type and Depp1^-/-^ mouse neonatal cardiomyocytes grown at hypoxia (1% O_2_) or normoxia for 18 hours. Data show mean fold change ±SEM, n=3, *P<0.05 by 2-way ANOVA with Šidák multiple comparisons test. **C**, Immunoblot analysis of wild-type or *Depp1*^*-/*-^ mouse neonatal cardiomyocytes stably expressing Keima-Pex26 treated, where indicated, with 250 nM Torin1, 100 µM FG-4592, or hypoxia (1% O_2_) for 18 hours. **D**, Immunoblot analysis of wild-type or *Depp1*^*-/*-^ mouse neonatal cardiomyocytes stably expressing Keima-OMP25 treated, where indicated, with 250 nM Torin1 or hypoxia (1% O_2_) for 18 hours. **E** and **F**, Immunoblot analysis of wild-type and DEPP1 gHRE cells stably expressing Keima-Pex26 or Keima-OMP25 grown at hypoxia (1% O_2_) or normoxia for 18 hours.

To test the role of DEPP1 in mitochondrial and peroxisomal autophagy in the setting of chronic HIF activation, we generated whole-body *Depp1*^*-/*-^ mice using CRISPR-Cas9–based gene editing of mouse embryos and crossed them to *VHL*^+/+^ or *VHL*^fl/fl^; αMHC-Cre mice (Figure S7A). The *Depp1*^*-/*-^ mice were viable, did not display gross phenotypes, and were normotensive, consistent with previous reports of similar mice (Figure S7B).^27,28^ *DEPP1* loss did not affect peroxisomal protein abundance or mitochondrial membrane potential by immunoblot and microscopy analysis (Figure S7C through S7E). We stably infected cardiomyocytes derived from the hearts of these mice with EGFP (enhanced GFP)–LC3, a reporter for autophagy induction, and Mito-RFP (red fluorescent protein), to visualize mitochondria. As expected, cardiomyocyte *VHL* loss promoted EGFP-LC3 puncta, indicative of autophagy induction, which colocalized with Mito-RFP, consistent with mitochondrial autophagy (Figure 4A; Figure S8A). *Depp1* loss in the setting of *VHL*^fl/fl^; αMHC-Cre abrogated EGFP-LC3 puncta, suggesting Depp1 is necessary for autophagy induction upon VHL loss (Figure 4A; Figure S8A). Consistent with these observations, *DEPP1* loss inhibited LC3B lipidation and p62 degradation mediated by either *VHL* loss or hypoxia in cells and heart tissue lysates in immunoblot assays (Figure S8B through S8D). *DEPP1* loss decreased mitochondrial swelling and increased mitochondrial oxygen consumption in the setting of hypoxia or *VHL* loss as measured by electron microscopy and Seahorse analysis (Figure S9A and S9B). Similarly, *DEPP1* loss reduced accumulation of triglycerides of chain lengths C50 to C60 in the setting of hypoxia and increased peroxisomal number in cardiomyocytes lacking *VHL* as measured by lipidomic analysis and confocal microscopy (Figure 4B; Figure S10A and S10B). *DEPP1* loss did not affect, positively or negatively, HIF stabilization in the setting of *VHL* loss or after FG-4592 treatment in isolated cells or in vivo (Figure 4C; Figure S8B and S8D).

To directly examine the role of DEPP1 in HIF-mediated peroxisome and mitochondrial autophagy, we stably expressed our Keima-PEX or OMP25 reporters in wild-type and *Depp1*^-/-^ cardiomyocytes. As expected, HIF activation through hypoxia or FG-4592, like mTOR inhibition by Torin1 or amino acid starvation, promoted Keima-PEX and Keima-OMP25 processing in wild-type cardiomyocytes as measured by immunoblot analysis (Figure 4C and 4D; Figure S11A through S11C). *Depp1* loss abrogated the Keima-PEX and Keima-OMP25 processing caused by HIF activation but did not affect the Keima processing caused by mTOR inhibition (Figure 4C and 4D; Figure S11A). Similarly, hypoxia and FG-4592 promoted Depp1-dependent mitochondrial (Mito-RFP or ATP5H) and peroxisomal (mRFP-PEX26 or PEX14) colocalization with LAMP1-positive lysosomes and LC3B-positive autophagosomes in cardiomyocytes by confocal microscopy (Figure S12A and S12B; Figure S13A and S13B; Figure S14A; Figure S15A). In addition, hypoxia promoted Depp1-dependent biotin-labeling of LAMP1 by APEX-PEX26, as measured by confocal microscopy using streptavidin-568 as a probe (Figure S15B).

We postulated the direct regulation of *DEPP1* by HIF was necessary to promote autophagy under hypoxia. Indeed, *DEPP1* HRE gene editing by CRISPR-Cas9 inhibited Keima-PEX processing, Keima-OMP25 processing, and p62 degradation under hypoxia (Figure 4E and 4F; Figure S16A). Similarly, *DEPP1* HRE gene editing inhibited autophagosome formation and their association with mitochondria under hypoxia but not Torin1 treatment, as measured by GFP-WIPI-I colocalization with Mito-RFP by live cell imaging (Figure S16B and S16C). Therefore, DEPP1, and its regulation by the HIF transcription factor, is necessary for HIF-mediated mitochondrial and peroxisome autophagy and triglyceride accumulation.

### DEPP1 Is Sufficient to Induce Autophagy, Triglyceride Accumulation, and Mitochondrial Permeability Transition Pore Activation

**Figure 5.**
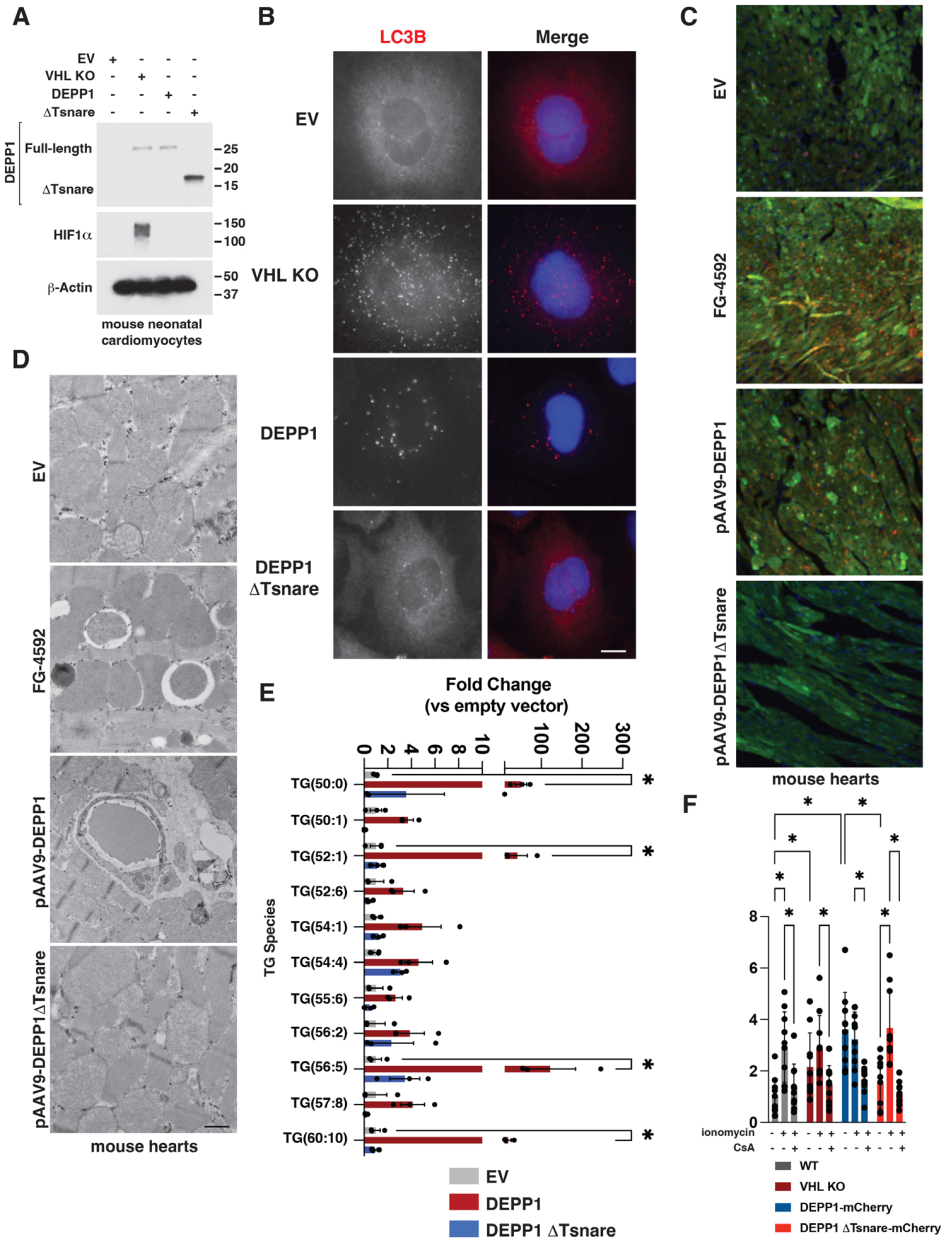
**DEPP1 expression is sufficient to induce autophagy, triglyceride accumulation, and mitochondrial permeability transition pore activation. A** and **B**, Immunoblot analysis (**A**) and fluorescence microscopy (**B**) of wild-type or *VHL* knockout mouse neonatal cardiomyocytes. Where indicated, the wild-type cardiomyocytes stably expressed wild-type or ΔTsnare DEPP1 or were transduced with an empty vector (EV). Scale bar=10 µm. **C** and **D**, Fluorescent microscopy (**C**) and electron micrographs (**D**) of heart cryosections derived from *CAG-RFP-EGFP-LC3* mice treated with 100 mg/kg FG-4592, or infected with an AAV encoding DEPP1, ΔTsnare DEPP1, or the empty vector (EV). n=3 per group, male mice 12 weeks old. Scale bar=20 µm in **C** and 500 nm in **D**. **E**, Triglyceride (TG) analysis in mouse neonatal cardiomyocytes stably expressing DEPP1, ΔTsnare DEPP1, or transduced with an empty vector (EV). Data show mean fold change ±SEM, n=3, *P<0.05 by 2-way ANOVA with Šidák multiple comparisons test. **F**, Mitochondrial permeability transition pore activation, as measured by quantification of calcein-AM fluorescence in live cells using Fiji analysis software, in wild-type, *VHL* knockout, or wild-type AC16 cardiomyocytes stably expressing wild-type or ΔTsnare DEPP1-mCherry. Where indicated, the cells were treated with 1 µM ionomycin, 1 µM cyclosporin A (CsA), or DMSO for 30 minutes before imaging. Data shown mean fold change ±SD, n=10, *P<0.05 by 2-way ANOVA with Šidák multiple comparisons test.

Hypoxia induces HIF, and HIF activation in mice is sufficient to promote many features of ischemic cardiomyopathy, including autophagy induction and lipid accumulation.^1–3,29^ To ask whether DEPP1 is sufficient to induce autophagy, we stably expressed DEPP1, DEPP1 ΔTsnare, or empty vector in cardiomyocytes (Figure 5A). Consistent with *VHL* loss, DEPP1, but not DEPP1 ΔTsnare, increased LC3B lipidation, as measured by fluorescent microscopy (Figure 5B; Figure S17A). These changes were not a result of exogenous DEPP1 indirectly inducing HIF (Figure 5A). We did reproducibly note that *VHL* loss caused more LC3B-positive puncta than exogenous DEPP1 in cardiomyocytes, although the endogenous DEPP1 levels achieved with *VHL* loss were similar to the exogenous levels. Among several possibilities, this could reflect a contribution of additional HIF target genes to autophagy after *VHL* loss.

To ask whether DEPP1 is sufficient to promote autophagy in vivo, we used *CAG-RFP-EGFP-LC3* transgenic mice in which a CAG promotor sequence drives the expression of RFP and an EGFP fused to LC3.^30^ These mice have been widely used to monitor autophagy in tissues as determined by the formation of RFP- and EGFP-positive puncta.^30^ We generated adeno-associated viruses serotype 9 (AAV9) expressing DEPP1 or DEPP1 ΔTsnare and then injected these viruses, or the empty vector, into the tail veins of *CAG-RFP-EGFP-LC3* mice and removed their hearts 7 days later. *CAG-RFP-EGFP-LC3* mice treated with FG-4592 served as controls. As seen with HIF activation by FG-4592, DEPP1, but not DEPP1 ΔTsnare, increased RFP and EGFP puncta in mouse hearts (Figure 5C). Ultrastructural analysis by electron microscopy of mouse hearts derived from mice treated with FG-4592 or expressing AAV9-DEPP1, but not DEPP1 ΔTsnare, revealed increased autophagosome formation and instances of mitochondrial engulfment by autophagosomes, suggestive of mitophagy (Figure 5D).

HIF promotes glucose and fatty acid uptake, aerobic glycolysis, and glucose to lipid conversion, while repressing oxidative phosphorylation and β-oxidation. Because HIF is necessary and sufficient for the accumulation of lipids in hearts lacking pVHL and chronic hypoxia increases triglyceride abundance,^8,31–33^ we tested whether DEPP1 is sufficient to promote cardiomyocyte triglyceride accumulation. Indeed, DEPP1, but not DEPP1 ΔTsnare, increased triacylglyceride abundance of chain lengths C50 to C60 in cardiomyocytes by lipidomic profiling (Figure 5E).

Chronic hypoxia is sufficient to induce mitochondrial permeability transition pore (MPTP) opening leading to cell death.^34–36^ To ask whether DEPP1 is sufficient to induce MPTP opening, we stably expressed DEPP1, DEPP1 ΔTsnare, in cardiomyocytes and treated these cells with and without ionomycin to trigger MPTP activation (Figure 5F). Like *VHL* loss, DEPP1, but not DEPP1 ΔTsnare, increased MPTP opening at baseline, as measured by confocal microscopy and image analysis (Figure 5F). MPTP opening was sensitive to cyclosporin A treatment, a classic MPTP inhibitor (Figure 5F).^36^ MPTP opening promotes apoptosis and cell death.^37^ We asked whether DEPP1 is sufficient to promote apoptosis. Although DEPP1 expression did not activate apoptosis at baseline, as measured by caspase-3 and PARP (Poly [ADP-ribose] polymerase 1) cleavage in immunoblot assays, DEPP1, but not DEPP1 ΔTsnare, increased the PARP and caspase-3 cleavage in response to staurosporine (Figure S17B).

### DEPP1 Loss Inhibits Hypoxia-Mediated ROS Production and HIF Stabilization

**Figure 6.**
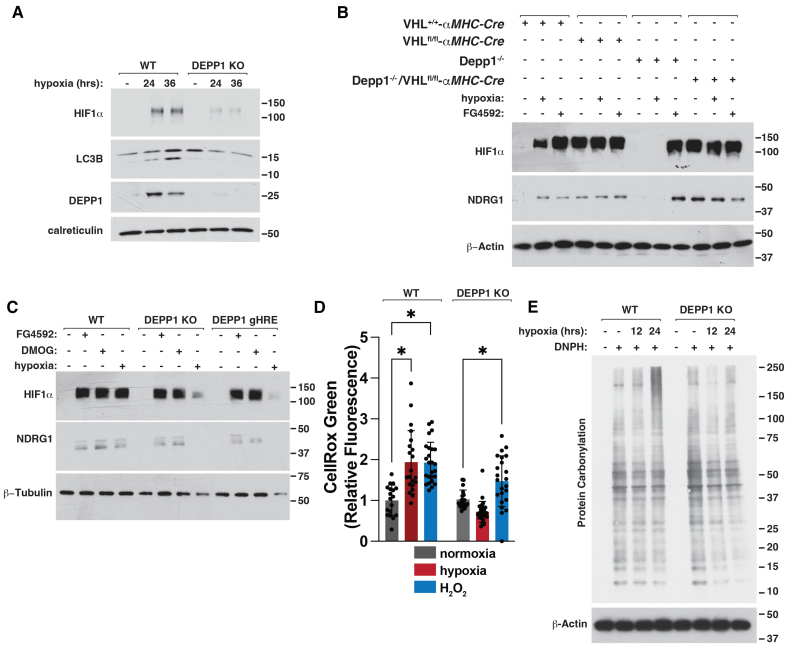
**DEPP1 is necessary for hypoxia mediated HIFα stabilization and ROS production. A**, Immunoblot analysis of wild-type or *DEPP1* knockout U2OS cells grown at hypoxia (1% O_2_) or normoxia for indicated time (hours). **B**, Immunoblot analysis of *VHL*^+/+^-, *VHL*^-/-^*-αMHC-Cre, Depp1*^-/-^*-αMHC-Cre*, or *Depp1*^-/-^/VHL^-/-^*-αMHC-Cre* mouse neonatal cardiomyocytes treated, where indicated, with 100 µM FG-4592 or hypoxia (1% O_2_) for 18 hours. **C**, Immunoblot analysis of wild-type, *DEPP1* knockout, or *DEPP1* gHRE cells treated, where indicated, with 100 µM FG-4592, 1 mM dimethyloxalylglycine (DMOG), or hypoxia (1% O_2_) for 18 hours. **D**, ROS production, as determined by quantification of CellRox Green fluorescence of live cells using Fiji analysis software, in wild-type and *Depp1* knockout mouse neonatal cardiomyocytes treated, where indicated, with 400 µM H_2_O_2_ or hypoxia (1% O_2_) for 18 hours. Data show mean fold change ±SD, n>10, *P<0.05 by 2-way ANOVA with Šidák multiple comparisons test. **E**, Immunoblot analysis of protein carbonylation in wild-type and *Depp1* knockout mouse neonatal cardiomyocytes grown at hypoxia (1% O_2_) or normoxia for indicated times (hours). Where indicated, the cell lysates were treated with 2,4-dinitrophenylhydrazine (DNPH) to detect protein carbonylation.

We frequently detected decreased HIF stabilization under hypoxia after *DEPP1* loss or disruption of the *DEPP1* HRE (Figure 2G; Figure 4C through 4F; Figure S11B; Figure S16A). *DEPP1* loss inhibited HIF stabilization and LC3B lipidation in U2OS cells grown under hypoxia for 24 to 36 hours (Figure 6A). The EglN-pVHL axis appeared to be functionally intact because cells lacking *DEPP1* or *DEPP1* HRE stabilized HIF when treated with FG-4592 or the prolyl hydroxylase inhibitor dimethyloxalylglycine, or after *VHL* inactivation (Figure 6B and 6C). Inhibition of hypoxia-mediated HIF stabilization in *DEPP1* knockout cells correlated with decreased abundance of the HIF-responsive gene product NDRG1 (Figure 6B and 6C). Mitochondrial ROS is essential for proper O_2_ sensing and subsequent HIF stabilization.^38–40^ We hypothesized that *DEPP1* loss reduces hypoxia-induced ROS production, thereby suppressing HIF. Indeed, *DEPP1* loss suppressed hypoxia-induced ROS production and oxidative stress, as measured by CellRox Green and protein carbonylation (Figure 6D and 6E). Therefore, DEPP1 is necessary for hypoxia-induced ROS and modulates HIF stabilization.

### DEPP1 Loss Increases Cardiomyocyte Survival After Chronic HIFα Activation and Decreases Cardiac Dysfunction Upon VHL Loss

**Figure 7.**
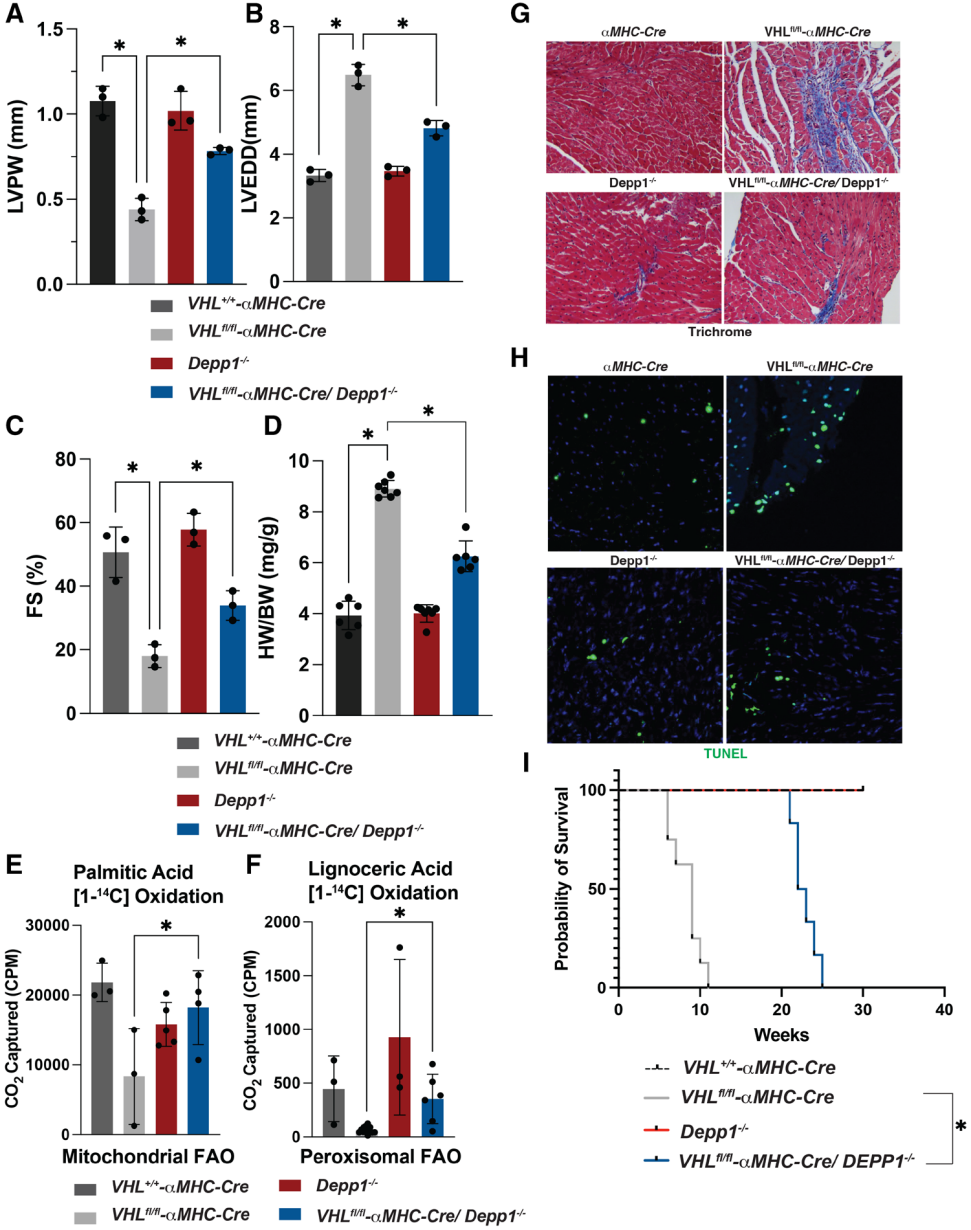
**DEPP1 loss decreases cardiac dysfunction upon VHL loss. A** through **C**, Echocardiographic measurement of left ventricular wall thickness (LVPW; **A**), left ventricular end diastolic diameter (LVEDD; **B**), and fractional shortening (FS; **C**) of mice with indicated genotypes at 8 weeks of age (n=3 mice per group, male), *P<0.05 by 1-way ANOVA with Šidák multiple comparisons test. **D**, Heart weight/body weight ratio (HW/BW) for 8-week-old mice with indicated genotypes (n>6 mice per group), *P<0.05 by 1-way ANOVA with Šidák multiple comparisons test. **E** and **F**, ^14^C-palmitic acid (**E**) or lignoceric acid (**F**) oxidation assay in mouse neonatal cardiomyocytes derived from mice with the indicated genotypes. n>3, *P<0.05 by 1-way ANOVA with Šidák multiple comparisons test. **G** and **H**, Trichrome (**G**) and TUNEL (**H**) staining of hearts from mice with the indicated genotypes (male, 8 weeks of age, 20× magnification). Propidium iodide (blue) is shown in **H** to stain nuclei. **I**, Kaplan-Meier survival curves for mice with indicated genotypes (n>5 per group). *P<0.05 by log-rank test.

*DEPP1* loss inhibits HIF-mediated autophagy of mitochondria and peroxisomes and triglyceride accumulation. To ask whether *DEPP1* loss would be beneficial or deleterious in the setting of chronic HIF activation, we used CRISPR-Cas9 to generate hIPS cells lacking *DEPP1* and then infected them or wild-type counterparts with a lentivirus encoding a doxycycline inducible non-hydroxylatable HIF2α variant (HIF2α-dPA). These cells were then induced to become cardiomyocytes (hiPS-CMs) and treated with doxycycline (Figure S18A).^41^ We previously showed that HIF2α-dPA is sufficient to cause cardiomyopathy in vivo.^1^ HIF2α-dPA increased hIPS-CM DEPP1 abundance and decreased hIPS-CM survival (Figure S18A and S18B). *DEPP1* loss increased hIPS-CM survival in the setting of HIF2α-dPA (Figure S18B). Consistent with this, whole-body *Depp1* loss decreased cardiac dysfunction in mice lacking pVHL in the heart as measured by myocardial thickness, fractional shortening, and cardiomegaly (Figure 7A through 7D). *Depp1* loss increased peroxisomal and mitochondrial fatty acid oxidation in isolated cardiomyocytes from hearts lacking pVHL (Figure 7E through 7F). Similarly, whole-body *Depp1* loss reduced cardiac fibrosis and apoptosis in hearts lacking pVHL (Figure 7G and 7H). These changes were associated with increased survival (median survival of ≈10 weeks for mice with hearts lacking pVHL and ≈22 weeks for mice with hearts lacking Depp1 and pVHL; Figure 7I).

HIF1α loss has been shown to completely rescue the cardiomyopathy and premature mortality in mice lacking cardiac pVHL.^2^ In contrast, *Depp1* loss extended, but did not fully rescue, the survival defect in mice lacking pVHL in the heart. Among multiple possibilities, this would suggest the contribution of additional HIF target genes to cardiomyopathy in the context of chronic HIF activation. Indeed, pVHL loss has been shown to result in cardiac inflammation, apoptotic cell death, and pathological hypertrophy through NF-κB (nuclear factor-κB) activation, and NF-κB inhibition is protective in the setting of heart failure.^42,43^ Whole-body *Depp1* loss did not reduce NF-κB activation in hearts lacking pVHL, as measured by NF-κB p62 S536 and IKKαβS176/S177 phosphorylation in immunoblot assays (Figure S19A). Similarly, pVHL-defective hearts had increased proinflammatory cytokine abundance, such as IL (interleukin)–6 and IL1β, that was unaffected by whole-body *Depp1* loss (Figure S19A). These results suggest that although *Depp1* loss decreases cardiac dysfunction in the setting of chronic HIF activation, pVHL loss still compromises cardiac function and longevity, at least in part, because of the Depp1-independent chronic activation of inflammation through NF-kB.

Because HIF activation is a predictable consequence of impaired oxygen delivery, we asked whether DEPP1 levels are increased in human ischemic cardiomyopathy. In the limited number of autopsy samples available to us from patients with chronic ischemic cardiomyopathy (n=3), DEPP1 and HIF abundance was increased, and this correlated with deregulated mitochondrial and peroxisomal protein abundance and increased LC3B lipidation by immunoblot analysis and decreased mitochondrial SDHA (succinate dehydrogenase) and PEX11B (peroxisomal biogenesis factor 11B) immunohistochemical reactivity (Figure S20A through S20C). Collectively, these results suggest that DEPP1 induction plays a key role in the pathogenesis of chronic ischemic cardiomyopathy and that its loss would decrease cardiac dysfunction in the setting of chronic HIF activation.

## DISCUSSION

Chronic HIF activation is a predictable consequence of chronic ischemia. HIF regulates many aspects of cardiac function, and the induction of cardiomyopathy by HIF is likely multifactorial. Therefore, understanding the contribution of HIF-regulated processes to heart failure and whether manipulation of HIF, or its downstream targets, could alter the natural course of this disease remains a key question. Here, we identify a mechanism by which hypoxia activates autophagy through the HIF pathway. We show in both cardiomyocytes as well as in noncardiac-derived cells, such as U2OS cells, that *DEPP1* is under direct control of the HIF transcription factor, localizes inside mitochondria, and is necessary and sufficient for HIF-mediated autophagy. *DEPP1* loss is protective in the setting of chronic HIF activation, and whole-body *Depp1* loss in mice decreases cardiac dysfunction and maladaptive metabolic remodeling after pVHL loss.

Patients with heart failure display systemic perturbations in energy-related metabolites, especially those reflecting mitochondrial and peroxisomal fatty acid metabolism, and derangement in cardiac substrate use is thought to contribute to contractile dysfunction and disease progression.^44,45^ These changes may reflect altered organelle biogenesis, increased autophagy, direct effects on fatty acid oxidation within mitochondria or peroxisomes, or a combination of all three. Mitochondrial autophagy plays a clear role in cardiac homeostasis, whereas the role of the peroxisome and its quality control remain understudied. Our studies identify DEPP1 as necessary and sufficient for HIF-mediated autophagy in cardiomyocytes. *DEPP1* loss increases mitochondrial and peroxisomal fatty acid oxidation and decreases cardiac dysfunction in the setting of chronic HIF activation. Similarly, *DEPP1* loss decreases triglyceride accumulation under hypoxia. Moreover, DEPP1 abundance is increased in ischemic cardiomyopathy patient biopsies, and this correlates with dysregulated mitochondrial and peroxisomal protein abundance.

Our work suggests increasing mitochondrial and peroxisomal abundance may provide a therapeutic strategy for ischemic cardiomyopathy. In this regard, activation of PPARα (peroxisome proliferator-activated receptor α), a key transcriptional activator of mitochondrial and peroxisomal fatty acid metabolism, improves cardiac function after pressure overload, ischemia, and pacing-induced heart failure in mice.^46–50^ Although PPARα agonists have not been directly tested in human heart failure trials, they were shown to reduce the risk of major cardiovascular events in the setting of type 2 diabetes, and this effect correlated with the degree to which they lowered serum lipids.^51,52^ Although cardioprotection mediated by PPARα activation presumably reflects, at least in part, reduced cardiotoxic lipid accumulation through increased extracardiac tissue lipid use, our results suggest that it could also confer cardiomyocyte-intrinsic protection in the setting of chronic ischemia by preserving mitochondrial and peroxisome abundance. Furthermore, it would be of interest to test whether PPARα agonists reduce cardiac dysfunction in the setting of chronic HIFα activation. In line with this possibility, peroxisomes are selectively essential under hypoxia, and peroxisomal fatty acid metabolism activation improves cardiac recovery after ischemia in mouse models.^53–55^

We find DEPP1 is necessary and sufficient for HIF-mediated autophagy, and *Depp1* inhibition decreases cardiac dysfunction in the setting of chronic HIF activation. Whether increased autophagy is an adaptive or maladaptive response to chronic myocardial ischemia remains a debate.^56,57^ In this regard, cardiomyocyte death in hearts from patients with end-stage heart failure is mediated most prominently through autophagy.^58^ Consistent with this, autophagy inhibition through heterozygous disruption of *Beclin 1* decreases cardiomyocyte autophagy and pathological cardiac remodeling after severe pressure overload in mice. Conversely, *Beclin 1* overexpression increases autophagy and pathological cardiac remodeling.^59^ Similarly, activation of Tfeb (transcription factor EB), a master regulator of lysosomal biogenesis and autophagy, causes heart failure in mice in the setting of pressure overload.^60^ It will be important to confirm experimentally whether increasing autophagy of mitochondrial and peroxisomes is sufficient to induce cardiac dysfunction. Further studies are necessary to understand whether autophagy inhibition is beneficial in the setting of chronic ischemia and heart failure.

Our studies do not preclude a protective role of HIF activation in the setting of acute myocardial ischemia. Indeed, HIF activates many genes that would be predicted to promote survival under conditions of acute hypoxia. PHD inhibition with pharmacologic inhibitors or genetic strategies, and transgenic expression of HIF1α in the heart, have been shown to be beneficial in animal models of acute myocardial infarction.^61–66^ Similarly, autophagy induction in the setting of acute ischemia reperfusion injury is believed to be beneficial rather than maladaptive. Autophagosome number increases in the heart during both the ischemia and reperfusion phases.^67^ Increased autophagy correlates with recovery and salvage of the myocardium in animal models of ischemia reperfusion injury and cardiomyocyte-specific ATG7 (Ubiquitin-like modifier-activating enzyme ATG7) loss, which enfeebles autophagy and aggravates ischemia reperfusion injury, leading to increased cardiac hypertrophy, dysfunction, and fibrosis.^68^ In addition, complete abrogation of autophagy is sufficient to trigger rapid-onset heart failure in mice, supporting a key role of basal levels of cardiomyocyte autophagy in cardiac homeostasis.^69^

DEPP1 appears to be a critical component of the metabolic control of autophagy in multiple contexts. We found that *DEPP1* is directly regulated by the HIF transcription factor, thereby connecting DEPP1 expression to cellular oxygen levels and autophagy. In addition, *DEPP1* mRNA is induced after insulin or nutrient deprivation, fasting, or H_2_O_2_ through the FOXO1/3 transcription factors and is necessary for FOXO3-mediated autophagy.^24,70–72^ FOXO activation is necessary and sufficient to induce fasting-mediated skeletal and cardiac muscle autophagy and atrophy.^73–76^ Because chronic exposure to high altitude leads to hypoxia-induced muscle wasting, it will be important to learn whether DEPP1 acts as a muscle atrophy–related factor. Similarly, autophagy-mediated mitochondrial clearance controls beige adipocyte maintenance, and autophagy inhibition prevents beige adipocyte loss and protects against diet-induced insulin resistance in mice.^77^ Because *Depp1* deficiency induces white adipocyte browning (“beiging”) and protects against diet-induced insulin resistance in mice, it would be of interest to understand DEPP1 and its role in adipocyte autophagy and adaptive thermogenesis.^28^

DEPP1 function remains to be determined. Mechanistically, DEPP1 is not a core component of the autophagy machinery because autophagy induction by mTOR inhibition is unaffected by *DEPP1* loss (Figure 4C and 4D). Instead, DEPP1 localizes inside mitochondria (Figure 3A through 3E). A recent study showed *DEPP1* inhibition suppressed MPTP opening in the setting of mitochondrial Ca^2+^ overload, a key step in cardiomyocyte death in the setting of ischemia, suggesting DEPP1 may play a role in mitochondrial Ca^2+^ homeostasis.^78,79^ In line with this, we find DEPP1 is sufficient to induce MPTP opening (Figure 5F). We hypothesize that DEPP1 regulates mitochondrial function to coordinate autophagy induction under conditions of low oxygen. Because DEPP1 is necessary and sufficient for hypoxia-mediated reactive oxygen species production, and mitochondrial ROS plays a clear role in hypoxia sensing and HIF pathway activation, precise understanding of how DEPP1 controls mitochondrial function and ROS production, how this is coordinated with associated peroxisomes, and how these signals regulate the autophagy machinery will need to be determined.

### Study Limitations

Our work demonstrates that *DEPP1* loss reduces cardiac dysfunction in the context of chronic HIF activation modeled through loss of pVHL in the heart. Although chronic HIF activation is a predictable consequence of chronic ischemia, the effects of pVHL loss and of chronic ischemia on the heart are unlikely to be entirely congruent. It will therefore be of interest to determine whether *DEPP1* loss is cardioprotective in other models of acute or chronic ischemia as well in the setting of other cardiomyopathies. Our work was restricted to isolated cardiomyocytes, immortalized U2OS osteosarcoma cells, and whole-body *Depp1* knockout mice. Because communication between cardiac resident cell types is known to provide cardioprotection, it will be of interest in future experiments to use mice in which *Depp1* is specifically deleted in cardiomyocytes or other cardiac resident cell populations, such as endothelial cells, epicardial cells, cardiac fibroblasts, and pericytes. Our mouse studies were restricted to 1 genetic background (C57/BL6) and 1 animal model (mouse). Our studies used male mice only because of their increased sensitivity to heart failure.^80^ It will be important to explore other strains and to test female mice. Because the mouse is an imperfect model of cardiovascular physiology, it will also be important to explore our findings in larger animal models.

## ARTICLE INFORMATION

### Acknowledgments

We thank the members of the Kaelin laboratory, J. Moslehi (University of California San Francisco), and R. Malhotra (Massachusetts General Hospital) for helpful discussions and critical reading of the article. Special thanks to the Cardiovascular Physiology Core at Brigham and Women's Hospital/Harvard Medical School (HMS) for assistance with cardiac physiology experiments, S. Drakos (University of Utah) for access to human heart samples and critical reading of the article, the Transgenic Mouse Core (HMS) for assistance with generation of CRISPR editing of mouse embryos, the HMS Electron Microscopy Facility for consultation and sample preparation, and the Beth Israel Deaconess Medical Center Mass Spectrometry Core facility for assistance with lipidomic profiling and analysis. This article is subject to HHMI’s Open Access to Publications policy. HHMI laboratory heads have previously granted a nonexclusive CC BY 4.0 license to the public and a sublicensable license to HHMI in their research articles. Pursuant to those licenses, the author-accepted article of this article can be made freely available under a CC BY 4.0 license immediately upon publication. G.A.W. performed experiments and, together with W.G.K., designed experiments, analyzed data, and assembled and wrote the article. Q.J. assisted in RNA sequencing sample preparation, sequence analysis, and experimental design. M.S. performed immunohistochemistry and assisted in confocal imaging. S.Q. performed quantitative polymerase chain reaction and apoptosis analysis. C.L. assisted in experimental design. B.A.M. assisted in human biopsy collection and sample preparation.

### Sources of Funding

W.G.K. is supported by the National Institutes of Health (grant No. 5R35CA210068) and is an HHMI investigator. G.A.W. is supported by the National Institutes of Health (grant No. 4R00HL163396; National Heart, Lung, and Blood Institute) and a Hassenfeld Award (MGH).

### Disclosures

W.G.K. has financial interests in Lilly Pharmaceuticals, Fibrogen, Nextech Invest, Tango Therapeutics, LifeMine Therapeutics, Circle Pharma, IconOVir Bio, and Casdin Capital. The other authors report no conflicts.

### Supplemental Material

Expanded Methods

Figures S1–S20

## Supplementary Material


