# Supplementary material for: Induction of DEPP1 by HIF Mediates Multiple Hallmarks of Ischemic Cardiomyopathy
Source: Circulation. 2024 Jun 17;150(10):770–86. doi: 10.1161/CIRCULATIONAHA.123.066628 (PMC11361356; doi:10.1161/CIRCULATIONAHA.123.066628)
Supplement: Supplementary file 1 [file cir-150-770-s001.pdf]

Supplemental Material

Induction of DEPP1 by HIF Mediates Multiple Hallmarks of Ischemic Cardiomyopathy

Gregory A. Wyant[1,4], Qinqin Jiang[1], Madhu Singh[4], Shariq Qayyum[4], Clara Levrero[1], Bradley A. Maron[2], William G. Kaelin, Jr[1,3,5,6]

[1]Department of Medical Oncology, Dana-Farber Cancer Institute, Boston, MA 02215, USA

[2]Department of Cardiovascular Medicine, Brigham and Women's Hospital, Harvard Medical School, Boston, MA 02215, USA

[3]Department of Medicine, Brigham and Women's Hospital, Harvard Medical School, Boston, MA 02215, USA

[4]Cardiovascular Research Center, Cardiology Division, Department of Medicine, Massachusetts General Hospital and Harvard Medical School, Boston, MA 02129, USA

[5]Howard Hughes Medical Institute, Chevy Chase, MD 20815, USA

[6]Corresponding author. Email: William_kaelin@dfci.harvard.edu

Methods

Cell Culture

293FT human embryonic kidney cells and U2OS osteosarcoma cells were originally obtained from the American Type Culture Collection (ATCC). AC16 human cardiomyocytes were originally obtained from Sigma-Aldrich. 293FT cells were maintained in Dulbecco's minimum essential medium (DMEM) supplemented with 10% fetal bovine serum (FBS), penicillin (100 U/ml), and streptomycin (100 µg/ml). Fresh aliquots of 293FT were thawed every 4 weeks. AC16 cells were maintained in DMEM/F12 medium supplemented with 15% FBS, penicillin (100 U/ml), and streptomycin (100 µg/ml). All experiments using AC16 cells were performed with passage 4-6 cells. Neonatal cardiomyocytes were isolated from mouse cardiac tissue (see below) and were maintained in DMEM supplemented with 15% FBS, penicillin (100 U/ml), and streptomycin (100 µg/ml). Human induced pluripotent stem cells (hIPSCS) were a gift from Dr. Christine Seidman. All hiPS-cardiomyocytes used were derived from the male parent iPSC line PGP1 (Personal Genome Project 1) derived from primary human skin fibroblasts and were mycoplasma-free. hIPSCs were grown on Matrigel (hESC-qualified, BD Biosciences) coated plates in mTeSR1 maintenance medium (StemCell Technologies) and media was replaced daily. Lentivirally infected cells were selected with puromycin (2 µg/ml) or blasticidin (10 µg/ml) as appropriate for the vector used. All mammalian cells were grown in a humidified atmosphere containing 21% oxygen and 5% CO_2 at 37°C unless otherwise stated.

Chemicals

Biotin Tyramide (Sigma) was prepared as a stock solution of 100 mM in DMSO and was diluted in cell culture medium at the indicated final concentration. Chloroquine (Selleck) was prepared as a stock solution of 30 mM in dH_2O and was diluted in cell culture medium at the indicated concentration. Torin1 (CST) was prepared as a stock solution of 1 mM in DMSO and was diluted in cell culture medium at the indicated final concentration. FG-4592 (Selleck) was prepared as a stock solution of 100 mM in DMSO and was diluted in cell culture medium at the indicated concentration. Dimethyloxalylglycine (DMOG) (Sigma) was prepared as a stock solution of 200 mM in DMSO and was diluted in cell culture medium at the indicated final concentration. Tamoxifen (Sigma) was prepared as a stock concentration of 200 mg/mL in ethanol. MitoTracker DeepRed FM (CST) was prepared at a 1 mM stock concentration in DMSO and was diluted in cell culture media at a final concentration of 500 nM. SAR405 (Selleck) was prepared as a stock solution of 200 mM in DMSO and was diluted in cell culture medium at the indicated final concentration. FCCP (Selleck) was prepared as a stock solution of 20 mM and was diluted in cell culture medium at the indicated final concentration. Oligomycin (CST) was prepared as a stock solution of 5 mM in DMSO and was diluted in cell culture medium at the indicated final concentration. Tetramethylrhodamine (TMRM) (Invitrogen) was prepared as a 100 mM stock in DMSO and was diluted in cell culture medium at the indicated final concentration.

Antibodies

Primary antibodies used were: Rabbit anti-FLAG (CST #14793), Mouse anti-FLAG (CST #8146), Rabbit anti-HA (CST #5017), Rabbit anti-citrate synthase (CST #14309), Mouse anti-citrate synthase (Santa Cruz sc-390693), Rabbit anti-VDAC (CST #4661), Rabbit anti-p70 S6 Kinase (CST #9202), Rabbit anti-LAMP1 (CST #9091), Mouse anti-LAMP2 (Santa Cruz sc-18822), Rabbit anti-PEX14 (Proteintech 10594-1-AP), Rabbit anti-Calreticulin (CST #12238), Rabbit anti-TOM20 (CST #42406), Rabbit anti-golgin97 (CST #13192), Rabbit anti-ARNT (CST #5537), Mouse anti- β-Actin (Santa Cruz sc-47778), Rabbit anti-HIF1A (CST #14179), Rabbit anti-LONP2 (ProteinTech 18035-1-AP), Rabbit anti-GNPAT (ProteinTech 14931-1-AP), Rabbit anti-MT-ND3 (Novus NBP2-93832), Rabbit anti-BNIP3 (CST #3769), Rabbit anti-BNIP3 (CST #44060), Rabbit anti-NDRG1 (CST #5196), Mouse anti-SQSTM1/p62 (Abcam ab56416), Rabbit anti-LC3B (CST #43566), Mouse anti-LC3B (CST #83506), Rabbit anti-catalase (CST #14097), Mouse Anti-Keima-Red (MBL International, M182-3M), Rabbit anti-Beclin1 (CST #3495), Rabbit anti-C10orf10/DEPP1 (ProteinTech 25833-1-AP), Rabbit anti-C10orf10/DEPP1 (Novus NBP2-38367), Rabbit anti-HIF2A (CST #71565), Rabbit anti-PEX5 (CST #83020), Rabbit anti-PEX3 (ProteinTech 10946-1-AP), Rabbit anti-TFAM (CST #8076), Mouse anti-PMP70 (Sigma SAB4200181), Rabbit anti-PEX19 (ProteinTech 14713-1-AP), Rabbit anti-ACOX1 (ProteinTech 10957-1-AP), Rabbit anti-ATP5H (ProteinTech 17589-1-AP), Rabbit anti-S6Kinase (CST #34475), Rabbit anti-PARP (CST #9542), Rabbit anti-Cleaved Caspase-3 (CST #9664), Rabbit anti-SDHA (CST #11998), and Rabbit anti-PEX11B (Invitrogen #PA5-37011). All primary antibodies used for immunoblot were diluted at 1:1000 in TBS-T+5% BSA.

cDNA Synthesis

Total RNA was extracted from 786-O, hIPS-CMs, 293FT, and U2OS cells using TRIzol Reagent (Life Technologies, 15596). cDNA was generated by reverse transcription using AffinityScript qPCR cDNA Synthesis kit (Agilent) according to manufacturer's instructions.

Plasmids

mCherry-TOMM20-N-10 was a gift from Michael Davidson (addgene plasmid: 55146; http://n2t.net/addgene:55146 ; RRID:Addgene_55146). pMXs-IP GFP-WIPI-1 was a gift from Noboru Mizushima (Addgene plasmid # 38272; http://n2t.net/addgene:38272; RRID:Addgene_38272). pMXs-IP-EGFP-LC3 was a gift from Noboru Mizushima (Addgene plasmid # 38195 ; http://n2t.net/addgene:38195 ; RRID:Addgene_38195). Tom70 (1-70)-GFP was a gift from Josef Kittler (Addgene plasmid # 127633 ; http://n2t.net/addgene:127633 ; RRID:Addgene_127633).

Cloning of cDNA expression vectors

To make expression vector pRK5-DEPP1-HA, a human DEPP1 cDNA was PCR amplified from from human 786-O cDNA using primers that introduce a 5'SalI and a 3' NotI site using the following primers:

SalI_DEPP1F: ACGCGTCGACGATGAGGTCCCGGCTTCTGCT
NotI_Depp1R: AAGGAAAAAAGCGGCCGCTCAGAGTTCATGGATCACCGGG

The resulting PCR product was digested using SalI and NotI, gel purified, ligated into a pRK5-HA vector linearized with these two enzymes and transformed into DH5a competent cells. Ampicillin-resistant colonies were verified by Sanger Sequencing.

To make expression vectors pMXS-IP Keima-PEX26, pMXS-IP Keima-PEX11A, or pMXS-IP Keima-OMP25, the Keima-fusion cDNA was synthesized as a dsDNA gBlock by Integrated DNA Technologies (IDT). The synthetic dsDNA fragment was then PCR amplified using the following primers to introduce a 5'EcoRI and 3'NotI site.

EcoRI_KozaK_mKeimaPEX26_F: GGAATTCGCCACCATGGTGAGTGTGATCGCTA
NotI_MkeimaPEX26_R: AAGGAAAAAAGCGGCCGCtcagtcacggatgcggagct

EcoRI_kozak_KeimaPEX11a_F: GGAATTCGCCACCATGGTGAGTGTGATCGCTA
NotI_Stop_Pex11a_R: AAGGAAAAAAGCGGCCGCCTAACGGGTCTTCAGCTTC

EcoRI_KOZAK_Keima_OMP25_F: GGAATTCGCCACCATGGTGAGTGTGATCGCTAA
NotI_Stop_keimaOMP25_R:
AAGGAAAAAAGCGGCCGCTCAGAGCTGCTTTCGGTATCTCAC

The resulting PCR products was then digested with EcoRI and NotI, gel purified, and ligated into pMXS-IP linearized with these two enzymes and transformed into HB101 bacteria. Ampicillin-resistant colonies were screened by EcoRI and NotI digestion and verified by Sanger sequencing.

To make the expression vectors pMXS-IP-DEPP1-FLAG-APEX, or pMXS-IP-DEPP1 DTsnare -FLAG-APEX, the DEPP1-fusion cDNA was synthesized as a dsDNA gBlock by Integrated DNA Technologies (IDT). The synthetic dsDNA fragment was then PCR amplified using primers that introduced a 5'EcoRI and 3'NotI site.

EcoRI_KOZAK_DEPP_F: GGAATTCGCCACCATGAGGTCCCGGCTTCTG
EcorI_Kozak_DeltaTsnare_F: GGAATTCGCCACCATGAGGCCACCCCACAGGCC
Not1_Stop_APEX_R: AAGGAAAAAAGCGGCCGCTTACGCCGCggcatcagc

The resulting PCR product was digested with EcoRI and NotI, gel purified, ligated into pMXS-IP linearized with these two enzymes and transformed into XL-10 gold ultracompetent cells. Ampicillin-resistant colonies were screened by EcoRI and NotI digestion and verified by Sanger sequencing.

To make the expression vectors pMXS-IP-mRFP-PEX26, the mRFP-PEX26 cDNA was synthesized as a dsDNA gBlock by Integrated DNA Technologies (IDT). The synthetic dsDNA fragment was then PCR amplified using primers that introduced a 5'EcoRI and 3'NotI site.

EcoRI_kozak_mRFPpex26_F: GGAATTCGCCACCatggcctcctccgaggac
NOT_mRFPpex26_R: AAGGAAAAAAGCGGCCGCtcagtcacggatgcggag

To make expression vectors pMXS-IP DEPP1-mCherry, pMXS-IP DEPP1-mNeongreen, or dTsnare truncation forms, the DEPP1-fusion cDNA was synthesized as a dsDNA gBlock by Integrated DNA Technologies (IDT). The synthetic dsDNA fragment was then PCR amplified using the following primers to introduce a 5'EcoRI and 3'NotI site.

EcoRI_KOZAK_DEPP_F: GGAATTCGCCACCATGAGGTCCCGGCTTCTG
EcorI_Kozak_DeltaTsnare_F: GGAATTCGCCACCATGAGGCCACCCCACAGGCC
Not_STOP_mCHERRY_R: AAGGAAAAAAGCGGCCGCctacttgtacagctcgtcca

CRISPR/Cas9 Plasmid Generation

The LentiCRISPR_v2-puromycin or -blasticidin vectors were used to express sgRNAs with Cas9. LentiCRISPR_v2 was digested with BsmBI, gel purified, and ligated with annealed oligonucleotides. The px459 sgRNA expression vector was digested with BbsI, gel purified, and ligated with annealed oligonucleotides.

Sense and antisense oligonucleotides corresponding to the desired sgRNA were mixed at equimolar ratios (0.25 nanomoles of each sense and antisense oligonucleotide) and annealed by heating to 100 °C in annealing buffer (1X T4 Ligase buffer, T4 PNK) followed by slow cooling to 30 °C over 3 hours. The annealed oligonucleotides were then diluted 1:200 in dH2O and ligated into the digested CRISPR vectors by incubation with T4 DNA ligase for 30 minutes at room temperature. The ligation reaction was transformed into XL-10 Gold ultracompetent cells and ampicillin-resistant colonies were verified by Sanger sequencing.

The following sgRNAs were used:
sgBeclin1F: caccgGAAACCAGGAGAGACCCAGG
sgBeclin1R: aaacCCTGGGTCTCTCCTGGTTTCc
sgARNTF: caccgTGGGGAACCTCACTTCGTGG
sgARNTR: aaacCCACGAAGTGAGGTTCCCCAc
sgDEPP1gHREF: caccgcacgtccacaccgcggtgaa
sgDEPP1gHRER: aaacttcaccgcggtgtggacgtgc
DEPP1g1F: caccgGATGGACAGCAGCAAGCCCA
DEPP1g1R: aaacTGGGCTTGCTGCTGTCCATCc

Lentivirus Generation and Infection

To make the lentiviruses, 1.3 x 10^6 HEK293FT cells were seeded in a 6-cm plate in DMEM supplemented with 10% FBS. Twenty-four hours later the cells were transfected with 1 µg of the desired lentivirus encoding plasmid together with of the packaging plasmids 0.5 µg psPAX2 and 0.5 µg pMD2.G using XtremeGene9 (Sigma) transfection reagent. Twelve hours after transfection, the medium was aspirated and replaced with 4 mL of

fresh medium. Thirty-six hour later the virus-containing supernatants were collected and passed through a 0.45-μm filter to eliminate contaminating mammalian cells.

For lentiviral infections, 1 x 10^6 cells target cells were combined with 250 μl of virus in 2 mL total volume of medium supplemented with 8 mg/mL polybrene and plated in 6-well plates. The plates were then immediately spun in an Eppendorf 581R centrifuge at 2,200 r.p.m. for 45 minutes at 37 °C. Twelve hours after infection, the virus-containing media was aspirated and replaced with fresh DMEM+10% FBS supplemented with Pen/Strep. Twenty-four hours post infection, the infected cells were trypsinized and replated in media that contained 2 μg/mL puromycin or 10 μg/mL blasticidin as appropriate for that virus.

For lentiviral infections involving mouse neonatal cardiomyocytes, cells were transduced with virus one day after isolation using low glucose DMEM + 7.5% FBS supplemented with polybrene (8 mg/ml) and Pen/Strep.

Baculovirus Infection

For experiments involving CellLight Peroxiosme-GFP, BacMam 2.0 Baculovirus (Invitrogen) or CellLight Lysosome-GFP, BacMam 2.0 Baculovirus (Invitrogen), 1.5 x 10^5 target cells were transduced in suspension with 20 μL baculovirus in a 3.5-cm plate. Twelve hours after baculovirus transduction, the media was replaced with fresh DMEM+10% FBS supplemented with Pen/Strep and grown for 36 hours. At the time of imaging, the media was aspirated and replaced with 2 mL phenol-red free medium (FluoroBriteDMEM, Thermo Fisher).

Adeno-associated virus 9

Human DEPP1 or DEPP1 ΔTsnare cDNA was cloned into the AAV9-cTNT vector using the NheI and NotI restriction sites with the following primers:

NheI_Kozak_DEPP1F: gctagcGCCACCATGAGGTCCCGGCTTCTGCT
NotI_DEPP1R: AAGGAAAAAAGCGGCCGCTCAGAGTTCATGGATCACC

AAV9 was packaged in 293T cells at 50% confluence with AAV9:Rep-Cap and pHelper (Cell Biolabs) and purified and concentrated from the supernatant of forty 15-cm dishes (4 x 10^6 cells per plate) per each AAV9 virus using the AAV purification Mega kit (Cell Biolabs). Concentrated AAV9 was then quantified using the AAV Rapid Quantitation kit (Cell Biolabs).

AAV9-cTNT-DEPP1, AAV9-cTNT- DEPP1 ΔTsnare, or the corresponding EV were delivered via tail vein injection into *CAG-RFP-EGFP-LC3* mice (age ~10-11 weeks, male only) at a dose of 3.5 x 10^{11} particles per mouse and were analyzed seven days post injection.

Immunoblot Analysis

Cells grown in 15-cm tissue culture dishes were washed once on ice with ice cold 1x phosphate-buffered saline (PBS) and then 1 mL ice cold PBS was added to each plate. The cells were then detached by scraping, transferred to a 1.5 mL Eppendorf tube, and pelleted at 1000 x g for 1 minute at 4 °C. The PBS was then aspirated, and the cell pellets were resuspended in lysis buffer (40 mM Hepes pH 7.4, 150 mM NaCl, 1.5 mM $MgCl_2$, 1% Triton-X 100, Complete Mini EDTA-free Protease Inhibitor (Sigma), and Phosstop Phosphatase Inhibitor (Sigma)) and lysed by gentle rocking for 20 minutes at 4 □C. For experiments involving Keima-fusion proteins, cell pellets were resuspended in RIPA buffer and lysed by gentle rocking for 20 minutes at 4°C. The lysate was then clarified by centrifugation at 17,000 x g for 10 minutes at 4°C and transferred to a new Eppendorf tube. The protein concentration of the whole cell extract was measured using the Bradford Assay and then normalized to 2 mg/mL. After normalization, the whole cell extract was denatured by the addition of 2.2% SDS, 11% glycerol, 100 mM DTT, and bromophenol blue. Samples were resolved by SDS-polyacrylamide gel electrophoresis using 4-20% Tris-Glycine gels (Novex) and wet transferred onto nitrocellulose membranes at 45 V for 2 hours. Membranes were blocked by incubation in 5% milk/tris-buffered saline + 0.1% Tween 20 (TBS-T) with gentle rocking for 1 hour at room temperature. The membranes were washed with TBS-T prior to overnight incubation with primary antibody diluted in TBS-T+5% BSA with gentle rocking at 4°C. After primary antibody incubation, membranes were washed three times with TBS-T and then incubated with horseradish peroxidase (HRP)-conjugated secondary antibody (1:5000) in 5% milk/TBS-T with gentle rocking for 1 hour at room temperature. The membranes were washed three times with TBS-T and bound antibodies were then detected with enhanced chemiluminescence western blotting reagents (Thermo Fisher Scientific, no. WBKLS0500) or Super-Signal West Pico (Thermo Fisher Scientific, no. PI34078).

Immunohistochemistry

All tissues were fixed with buffered 10% formalin solution (SF93-20; Fisher). Mouse Heart tissues were perfusion fixed. For hematoxylin and eosin and trichrome staining, tissues were embedded in paraffin prior to sectioning. Paraffin-embedded human tissue sections were immunostained for Rabbit anti-SDHA (CST #11998) and Rabbit anti-PEX11B (Invitrogen #PA5-37011) using the Cell Signaling Immunohistochemistry Paraffin Protocol for SignalStain Boost Detection. Antigen retrieval was performed by heating slides in citrate buffer in a microwave until boiling was initiated followed by 10 minutes at sub-boiling temperatures (95-98°C). Slides were then left at room temperature for 30 minutes to cool prior to being peroxide blocked with 3% hydrogen peroxide for 10 minutes followed by 1 hr in Tris Buffered Saline with 1% Tween 20 and 5% Normal Goat Serum (blocking solution). Following blocking, slides were incubated with indicated primary antibodies. Staining was developed using a DAB substrate kit (CST #8059) and mounted using SignalStain Mounting Medium (#14177). Photomicrographs were obtained with a Leica DM4B microscope and a DMC4500 camera (20X objective lens). The technician performing immunohistochemistry was blinded to sample identifiers and treatments.

hIPSC-Cardiomyocyte Differentiation

The hiPS-cardiomyocytes were generated using a small-molecule mediated differentiation protocol that modulates Wnt signaling[42]. hIPS cells were grown in 6-well plates pre-coated with Matrigel extracellular matrix in mTeSR1 (StemCell Technologies) pluripotent stem cell growth medium containing 10 μM rho kinase inhibitor Y-27632 (Selleck). Following 24 hour after plating, media was replaced with fresh mTeSR1 without Y-27632 and hIPS cells were grown to 80% confluency with daily replacement of mTeSR1. Upon reaching 80% confluency, mTeSR1 media was aspirated and 2 mL RPMI 1640 medium containing B27 supplement without insulin (StemCell Technologies) and 12 μM CHIR99021 (Selleck) was added to each well. Following 24 hours of CHIR99021 treatment (day 1 of differentiation), media was aspirated and replaced with fresh RPMI 1640 medium (with B27 supplement without insulin) and grown for 48 hours. After 48 hours (day 3 of differentiation), RPMI 1640 media was aspirated and replaced with fresh RPMI 1640 (with B27 supplement without insulin) containing 2 μM Wnt-C59 (Selleck) and grown for 48 hours. After 48 hours (day 5 of differentiation), RPMI 1640 media was aspirated and replaced with fresh RPMI 1640 medium (with B27 supplement without insulin) and grown for 48 hours (day 7 of differentiation). Cells began beating at approximately day 7 post-differentiation. Once beating began, cardiomyocytes were metabolically selected from other differentiated cells by glucose deprivation. RPMI 1640 media was aspirated and replaced with fresh RPMI 1640 media without glucose (with B27 supplement with insulin) for 48 hours.

Gene Editing in Human iPS-Cardiomyocytes

PGP1 hiPSCs were electroporated with 2 μg plasmid expressing Cas9 (PX459 v2 from Addgene) and 2 μg plasmid expressing guide RNA using a stem cell nucleofector kit. Electroporated hIPSCs plated into 6-well plates and selected by growth in MTeSR1 containing puromycin overnight followed by 24 hours in mTeSR1 without puromycin. Puromycin-resistant hIPSCs were then replated into 6-well plates by serial dilution where a 6-well plate receives 1:2, 1:4, 1:16, 1:32, and 1:64 dilutions of cells to obtain a cell distribution where individual hiPSCs grow into a monoclonal colony. Individual colonies were then expanded and differentiated into cardiomyocytes.

Radioactive Fatty Acid Oxidation (FAO) Assay

Radiolabeled fatty acids (^{14}C-labeled palmitate and ^{14}C-labeled lignoceric acid) were purchased from American Radiolabeled Chemicals solubilized in ethanol. To prepare ^{14}C-labeled fatty acid for FAO assay, 1-2 mCi C^{14}-labeled palmitate and lignoceric acid were dried under nitrogen in an Eppendorf tube to remove ethanol and resolubilized in a BSA-fatty acid mixture (7%BSA, unlabeled 2.5 mM palmitate or lignoceric acid) in dH_2O. Following solubilization, the radioactive BSA-fatty acid mixture was heated at 37°C for 30 minutes with frequent vortexing and stored overnight at 37°C.

Mouse neonatal cardiomyocytes were grown in 6-well plates at a density of 200,000 cells per well and grown to confluency. At time of experiment, the cells were washed, and the media was replaced with DMEM without serum containing 1 mM

unlabeled carnitine and the radioactive BSA-fatty acid mixture at a final media concentration of 0.3%BSA/100 mM fatty acid/0.4 mCi/mL ^{14}C-fatty acid. The 6-well plate was then sealed with parafilm for 3 hours. To measure radioactive FAO, a Whatman paper ($^{14}CO_2$-trap) circle corresponding to the size of the inside of an Eppendorf tube cap was cut. Following, 20 µL of 1 M NaOH was absorbed onto the $^{14}CO_2$-trap and then the $^{14}CO_2$-trap was placed inside the cap of an Eppendorf tube containing 200 µL of 1M perchloric acid. Following the 3-hour incubation, the parafilm was removed from the 6-well plate and the entire reaction was transferred into the Eppendorf tubes containing perchloric acid and $^{14}CO_2$-trap. The Eppendorf was then closed and incubated for 1 hour at room temperature. Following 1 hour, the filter paper disc was removed using tweezers and placed into scintillation vials and counted for ^{14}C-radioactive signal.

Subcellular Fractionation

Cells were plated in 10-cm plates at a density of 4 x 10^5 cells per plate and grown to 80% confluence. At the time of collection, the cells were washed twice with ice cold PBS and once with ice cold Hepes-sucrose buffer (HSB) (250 mM sucrose, 20 mM Hepes-KOH pH 7.4) containing 1 mM EDTA and protease inhibitors. Next, 400 µL of HSB containing 100 mg/mL digitonin (Wako Chemicals) was added to each plate and scraped into a 1.5 mL Eppendorf tube and incubated on ice for 10 minutes. Cells were divided into two different 1.5 mL Eppendorf tubes (180 µL/tube) for whole cell extract and a cytosolic/membrane fraction. The whole cell extract was solubilized by the addition of 36 µL of 6x SDS-PAGE sample buffer. The cytosolic/membrane fraction was centrifuged at 20,000 x g for 15 minutes at 4 °C. After centrifugation, 150 µL of the supernatant (cytosolic fraction) was transferred into a new 1.5 mL Eppendorf tube and solubilized by the addition of 33 µL 6x SDS-PAGE sample buffer. The remaining pellet was washed once in 50 µL HSB and then centrifuged at 20,000 x g for 15 minutes at 4°C. After centrifugation, the HSB was aspirated and 200 µL of 1x SDS-PAGE sample buffer was added to the membrane pellet and sonicated. All samples were boiled for 5 minutes prior to western blotting.

Chromatin Immunoprecipitation (ChIP) PCR

To assess HIF1α bound to the DEPP1 promoter in cardiomyocytes, HIF1α was chromatin immunoprecipitated using HIF-1α XP anti-rabbit (CST #36169) and ChIP-IT Express Enzymatic Kit (Active Motif).

To prepare chromatin, AC16 human cardiomyocytes were plated in 15-cm plates at a density of 8 x 10^5 cells per plate and grown until 80% confluence. At time of cell harvest, media was aspirated and 20 mL of fixation solution (540 µL of 37% formaldehyde was added to 20 mL DMEM) was added to each plate and cells were incubated at room temperature gently shaking for 10 minutes. Fixation solution was then removed, and cells were washed in ice cold PBS followed by the addition of 10 mL Glycine Stop solution and placed at room temperature for 5 minutes. The glycine Stop solution was then aspirated after which the cells were washed once in ice cold PBS followed by the addition of 1 mL ice-cold lysis buffer. The cells were left on ice for 30 minutes and then dounce

homogenized in a 2 mL dounce (10 strokes). An enzymatic shearing solution was prepared by diluting enzyme mix ($2x10^4$ U/ml) 1:100 into 50/50 glycerol dH_2O. The homogenate was transferred to a 1.5 mL Eppendorf tube and then centrifuged for 10 minutes at 5,000 rpm (2,400 RCF) at 4°C. Following centrifugation, the supernatant was aspirated, and the pellet was resuspended in 350 µL Digestion Buffer and placed at 37°C for 5 minutes followed by the addition of 17 µL of the Enzymatic shearing solution and the addition of 7 µL 0.5 M EDTA for 10 minutes on ice. After 10 minutes, the samples were centrifuged at 15,000 rpm for 10 minutes at 4°C and the supernatant containing chromatin was placed in a new 1.5 mL Eppendorf tube.

Following chromatin isolation, a 100 µL ChIP reaction was prepared in a 1.5 mL Eppendorf tube by combining 25 µL Protein G Magnetic Beads, 10 µL ChIP Buffer, 25 µg Chromatin, 2 µg HIF-1α XP anti-rabbit antibody, and dH_2O, which was then incubated for 4 hours at 4°C. The Eppendorf tubes were then placed on a magnetic rack to pellet the beads. The supernatant was aspirated, and the beads were washed 3 times in 1 mL ChIP Buffer. The bound chromatin was then eluted by the addition of 50 µL of Elution Buffer followed by rocking for 15 minutes at room temperature. The elution was then mixed with 50 µL Reverse Cross linking Buffer and the tubes were placed back on the magnetic rack. The supernatant containing chromatin was then transferred to a new 1.5 mL Eppendorf tube and placed at 95°C for 15 minutes followed by the addition of 2 µL Proteinase K for 1 hour at 37°C. After 1 hour, 2 µL Proteinase K Stop solution was added. At this stage, processed chromatin was utilized for End-point PCR utilizing the following program and primers:

94 °C - 3minutes

35 cycles (94 °C -20 seconds, 59 °C -30 seconds, 72 °C -30 seconds)

10 °C hold

deppCHIPF1: cacagcgcagaacacagtg
deppCHIPR1: GTCCACACCGCGGTGAAG

Mice

The *VHL*$^{fl/fl}$ and aMHC-Cre mice were imported from Jackson Laboratory and were previously described. DEPP1$^{-/-}$ mice were generated by utilizing two guide RNAs (sgRNAs) targeting the mouse DEPP1 gene using CRISPR-Cas9 injection into C57/BL6 mouse zygotes. In brief, to generate mDEPP1 sgRNA, a gblock with a built-in T7 priming sequence and the guide/scaffold sequence was synthesized from IDT using the following sequence:

mDEPP1g1 T7:
cgctgTTAATACGACTCACTATAGGGCCTCAGTGCTGGACAAGGTCGTTTTAGAGCTAGAAAtagcaagttaaaataaggctagtccgttatcaacttgaaaaagtggcaccgagtcggtgcTTTT

mDEPP1g2 T7:
cgctgTTAATACGACTCACTATAGGGGGAGAGCAGGCAGATGGGAGGTTTTAGAGCTAGAAAtagcaagttaaaataaggctagtccgttatcaacttgaaaaagtggcaccgagtcggtgcTTTT

The lyophilized gblock was reconstituted in 10 µL PCR-grade water to make a 20 ng/µL stock. RNA was synthesized in vitro from the gblock using MEGAshortscript T7 Kit (LifeTech) using 8 µL of the reconstituted gblock as a template. To capture the RNA, 50 µL Agencourt RNAClean XP beads (A63987) were added to the synthesized RNA and incubated at room temperature for 10 minutes. The beads were then collected using 96-well plate magnet and washed 3 times in 80% EtOH. After the final EtOH wash, the ethanol was aspirated, and the beads were left to dry and resuspended in HyClone Molecular Biology-Grade Water (GE Healthcare Life Sciences). The beads were then placed back on the magnet and the supernatant was collected. The RNA concentration was quantified by nanodrop and then immediately frozen at -80°C until injection.

Zygote injections were performed at the HMS Transgenic Facility. All procedures were performed according to National Institutes of Health guidelines and approved by the Committee on Animal Care at HMS. Female 8-10 week old C57/BL6 mice were superovulated by IP injection of 5 IU of pregnant mare serum gonadotropin (367222-1000IU; EMD Millipore) followed 46–48 hours later by 5 IU human chorionic gonadotropin (80051–032; VWR). Superovulated female mice were then mated to stud males. Fertilized pronuclear stage embryos (zygotes) were collected ~20 hours after injection of human chorionic gonadotropin. Cytoplasmic injections were performed using a Piezo actuator (PMM-150FU; Prime Tech) and a flat-tip microinjection pipette with an internal diameter of 8 µm (Origio). The injection mix was prepared immediately before the procedure and included the following components at the final concentrations indicated: 100 ng/µl Cas9 mRNA (Sigma Aldrich), and 50 ng/µl sgRNA. Immediately after the completion of the injection, zygotes were transferred into the oviducts of pseudopregnant females at 0.5 dpc.

For genotyping *Depp1*$^{-/-}$ animals, tails snips were collected into tubes with 500 µl QuickExtract buffer (Epicentre). To obtain PCR-ready genomic DNA, the tubes were incubated at 65°C for 10 minutes, followed by a quick vortex and a 2-minute incubation at 98 °C. The insoluble material was removed by centrifugation at 13,000 x g for 10 minutes at 4 °C. To genotype by PCR, 5 µl of supernatant was used as a PCR template and the following primers were used

mDEPP1Fwd: 5'- tcgccactggtttcctcttg -3'
mDEPP1Rev: 5'- GAGTTCATGGATCACTGGGAGG-3'

PCR was done with Q5 High Fidelity 2X Master Mix (New England Biolabs) at 98 °C for 30 seconds, 30 cycles of 98 °C for 10 seconds, 64 °C for 30 seconds, and 72 °C for 30 seconds, and a final extension time of 72 °C for 2 minutes. PCR products were sequenced by Amplicon sequencing.

Isolation and Culture of Primary Mouse Neonatal Cardiomyocytes

Neonatal mice were sacrificed within the first 24 hours after birth. Beating hearts were removed and placed on ice in Hanks Balanced Salt Solution (HBSS) supplemented with sodium bicarbonate (1.6 mM). The great vessels were dissected away, and the hearts were minced with a razor blade. Minced hearts (~40 hearts) were placed in 15 mL conical tubes containing HBSS with trypsin (1 mg/mL) for 2 hours at 4 °C. The tubes were then centrifuged at 15000 x g for 10 minutes at 4 °C. The supernatant was collected, and the minced hearts were subjected to 5 digestion steps (3 minutes each) with Collagenase Type II (125 U/mL) in HBSS at 37 °C. After each 3 minutes digestion, the supernatant was removed and diluted in isolation media (low glucose DMEM + 15% FBS supplemented with Pen/Strep). The cells were filtered (100 µm) and subjected to a Percoll gradient centrifugation (2100 x g for 30 minutes). The pellet was then resuspended in isolation media and cultured overnight on fibronectin coated 10-cm plates.

Fluorescence Microscopy Analysis of APEX2 Biotin-Phenol Labeling in Cardiomyocytes

AC16 human or mouse neonatal cardiomyocytes stably expressing APEX-fusion proteins were plated on coverslips at 50% confluence. Cardiomyocytes were pre-incubated with 500 µM biotin-tyramide in cell culture media for 30 minutes in a 5% CO_2 atmosphere at 37 °C. After the indicated treatments, H_2O_2 was added to a final concentration of 1 mM for 1 minute at room temperature. The reaction was then quenched by washing the coverslips three times with 2 mL quencher solution (10 mM sodium ascorbate, 10 mM sodium azide, and 5 mM Trolox in DPBS) followed by two washes with PBS. Following quenching, biotin-labeled cells were fixed with 4% paraformaldehyde in PBS for 15 minutes at room temperature. Coverslips were then washed three times with PBS and permeabilized with 0.05% TritonX-100 in PBS for 5 minutes at room temperature. Coverslips were then washed three times with PBS and then blocked with Odyssey Blocking buffer for 1 hour at room temperature. To detect biotinylated proteins, coverslips were incubated with streptavidin-Alexa Fluor 568 or 488 IgG (1:400 dilution) for 45 minutes at room temperature in the dark prior to DAPI staining and slide mounting.

Lipidomics

Nonpolar lipids were extracted from mouse neonatal cardiomyocytes grown in 6-well plates seeded at a density of 2×10^5 cells per well. At the time of extraction, the cells were washed twice in ice cold PBS and harvested into 100 µL PBS in an Eppendorf tube. The cells were then pelleted by centrifugation at 1000 x g for 2 minutes at 4 °C after which the PBS was aspirated. The cell pellets were then resuspended in ice cold chloroform:methanol (2:1 ratio) at a final volume 20 times the volume of the biological sample (100 µL sample in 2 mL of solvent mixture) for lipid extraction. The samples were then agitated for 30 minutes on an orbital shaker at 4 °C followed by the addition of 0.2 volume parts of water (400 µL for 2 mL sample) and vortexed for 1 minute. The samples were then placed on a flat surface for 10 minutes followed by low-speed centrifugation (1000 x g for 1 minute) to separate phases. The lower phase containing the nonpolar lipids was dried in a SpeedVac rotary evaporator with no heat. Lipid samples were

resuspended in 35 µl of 50% isopropanol/50% MeOH. 10 µl of samples was injected for reversed-phase (C_{18}) LC–MS/MS with a hybrid QExactive Plus Orbitrap mass spectrometer (Thermo Fisher Scientific) coupled to an Agilent 1100 HPLC in DDA mode with positive/negative-ion polarity switching (top 8 in both modes). The lipidomics data were analyzed with LipidSearch 4.1.9 software. The software identifies intact lipid molecules based on their molecular weight and fragmentation pattern by using an internal library of predicted fragment ions per lipid class, the spectra are then aligned based on retention time, and MS1 peak areas are quantified across sample conditions.

Immunofluorescence Staining

For immunofluorescence assays, mouse neonatal cardiomyocytes were seeded and cultured on coverslips in 6-well plates under the desired conditions. To fix the cells, the media was aspirated, and the cells were washed twice with cold 1X PBS and fixed using 4% paraformaldehyde in 1X PBS or ice cold 100% methanol for 15 minutes at room temperature. Paraformaldehyde-fixed cells were then permeabilized by incubation in 0.05% TritonX-100 in 1X PBS for 5 minutes at room temperature. The coverslips were blocked with Odyssey Blocking Buffer (Li-Cor) for 1 hour at room temperature. The cells were subsequently stained sequentially with primary and secondary antibody diluted in Odyssey blocking buffer for 1 hour each at room temperature, being washed thrice after both primary and secondary antibody staining. The cells were then counterstained with DAPI and mounted onto glass slides using mounting medium and imaged within 48 hours. Anti-mouse Alexa Fluor 488 and Anti-rabbit Alexa Fluor 568 secondary antibodies (Invitrogen) were used at 1:400 dilution.

Confocal Microscopy

Live cell confocal microscopy was performed using a Zeiss LSM 880 confocal microscope equipped with Airyscan (Fast) detector using a 63 x Plan-Apochromat oil objective, NA 1.4. In brief, 2×10^5 cells were plated onto 35 mm-glass bottom dishes (No. 1.5, 14 mm glass diameter, MatTek) then incubated in phenol-red free medium (FluoroBriteDMEM, Thermo Fisher) containing 10% fetal bovine serum for 24 hours prior to drug treatment or oxygen deprivation. Where indicated, cells were incubated with 1 µM Hoechst 33342 15 minutes prior to imaging. At time of imaging, cells were imaged in FluoroBrite DMEM without 10% fetal bovine serum. Series optical sections were collected with a step-size of 0.22 microns. Z series were displayed as maximum z-projections, and gamma, brightness, and contrast were adjusted for each image equally using FiJi software. At least five to six image frames per condition were analyzed without exclusion.

For imaging experiments involving Keima, live cell confocal microscopy was performed using a Zeiss LSM 900 confocal microscope using a 63 x Plan-Apochromat oil objective, NA 1.4. In brief, 2×10^5 cells were plated onto 35 mm-glass bottom dishes (No. 1.5, 14 mm glass diameter, MatTek) then incubated in phenol-red free medium (FluoroBriteDMEM, Thermo Fisher) containing 10% fetal bovine serum for 24 hours prior to drug treatment or oxygen deprivation. At time of imaging, cells were imaged in FluoroBrite DMEM without 10% fetal bovine serum. Pairs of images for ratiometric

analysis of Keima fluorescence were sequentially collected using 442 nm and 561 solid state lasers and emission with a 620 nm filter. Series optical sections were collected with a step-size of 0.22 microns. Z series were displayed as maximum z-projections, and gamma, brightness, and contrast were adjusted for each image equally using FiJi software. Areas expressing Keima-reporter were detected by the Otsu thresholding algorithm in Fiji. Using the 'Calculator Plus' tool in Fiji, the signal intensity in the 561 nm channel was divided by the signal intensity in the 488 nm channel and normalization to the control sample.

Seahorse analysis of oxygen consumption rate (OCR)

Cardiomyocytes (10,000 cells/well) were plated in XF-96 plates. The next morning, the cells were washed once with 1xPBS and incubated overnight in serum-free DMEM. The cells were then washed once and incubated for 1 hour in XF assay medium (DMEM pH 7.4 with 10 mM glucose, 2 mM L-glutamine, 1 mM pyruvate) in a non-CO_2 incubator per manufacturer's instructions (Seahorse Agilent). Real-time measurements of oxygen consumption rate (OCR) were performed using an XF-96 Extracellular Flux Analyzer (Agilent). Three or more consecutive measurements were obtained under basal conditions and after the sequential addition of 1.5 µM oligomycin to inhibit mitochondrial ATP synthase; 1 µM FCCP (fluoro-carbonyl cyanide phenylhydrazone), a protonophore that uncouples ATP synthesis from oxygen consumption by the electron-transport chain; and 500 nM rotenone plus antimycin A, which inhibits the electron transport chain.

Mitochondrial Permeability Transition Pore Assay

The mitochondrial permeability transition pore opening was measured using the Image-iT LIVE mitochondrial transition pore assay kit (I35103, Invitrogen) following the protocol provided by the manufacturer with the following modifications. Human AC16 cardiomyocytes (100,000 cells) were plated in 35 mm-glass bottom dishes (No. 1.5, 14 mm glass diameter, MatTek). The following day the cells were washed with Hanks' Balanced Salt Solution (HBSS)(Invitrogen) supplemented with HEPES (10 mM), L-glutamine (2 mM) and succinate (100 µM) and then incubated with 1 mL HBSS containing 1 µM Calcin AM, 200 nM, MitoTracker Red, 1 µM Hoechst 33342, and 1 mM $CoCl_2$ for 15 minutes at 37°C. After incubation, the cells were washed once in HBSS followed by the addition of 2 mL HBSS prior to imaging. Where indicated, the cells were treated with 500 nM ionomycin or 500 nM cyclosporin A for 15 minutes prior to imaging. Cells were then imaged using a Zeiss LSM900 confocal microscope. At least 10 image frames per condition were analyzed without exclusion using FiJi software.

CellROX Green ROS Quantification

Mouse neonatal cardiomyocytes were plated in DMEM/F12 medium with 10% FBS and 1% pen/strep in 6-cm dishes at a density of 2.5 x 10^5 cells per plate prior to ROS quantification and grown for 24 hours. The media was then removed and replaced with DMEM containing no serum supplemented with CellRox Green (5 µM) for 30 minutes. The cells were then washed once with DMEM containing no serum followed by the

addition 3 mL phenol-red free medium (FluoroBriteDMEM, Thermo Fisher) prior to imaging. Cells were then imaged using a Zeiss and 10 image frames per condition were analyzed without exclusion using FiJi software.

TUNEL apoptosis assay

Paraffin sections (5 μm) of mouse hearts fixed in 10% phosphate-buffered formalin were analyzed by TUNEL (Promega, G3250) according to manufacturer's instructions. Deparaffinized heart sections were fixed in 4% formaldehyde for 15 minutes and then immersed in PBS twice for 5 minutes. The fixed tissues were then permeabilized using 100 μL proteinase K (20 μg/ml) at room temperature for 10 minutes. After permeabilization, the tissues were immersed in PBS for 5 minutes and fixed a second time in 4% formaldehyde at room temperature for 5 minutes followed by PBS for 5 minutes. The tissue sections were then incubated with 100 μL of equilibration buffer and placed at room temperature for 10 minutes. Following equilibration, 50 μL rTdT buffer containing 45 μL equilibration buffer, 5 μL nucleotide mix, and 1 μL rTDT enzyme was added to each tissue section and the slides were incubated at 37°C for 1 hour in a humidified chamber protected from light. After 1 hour, the tissue sections were immersed in 2X SSC buffer for 15 minutes at room temperature followed by three washes with PBS for 5 minutes each to remove the unincorporated fluorescein-12-dUTP. The sections were then stained with propidium iodide for 15 minutes at room temperature in the dark followed by three washes with deionized water for 5 minutes at room temperature. The excess water was removed by blotting with kimwipes before mounting the tissue sections on glass coverslips. The tissue sections were analyzed using a Zeiss LSM 900 confocal microscope using a 20X objective. At least 10 fields were randomly selected without exclusion.

STED Microscopy

STED super resolution microscopy was performed using a Abberior STEDYCON equipped with 4 channels with capabilities for simultaneous detection as a point scanning confocal and 2 STED channels. Imaging was done within 48 hours after fixing and staining as described in immunofluorescence using Star RED and Star ORANGE secondary antibodies. STED images were deconvolved using Huygens software and gamma, brightness, and contrast were adjusted for each image equally using FiJi software.

Electron Microscopy

Mouse neonatal cardiomyocytes were grown to 60% confluence in 10-cm plates. The cells were rapidly fixed in 1.25% paraformaldehyde, 2.5% glutaraldehyde, 0.03% picric acid followed by osmication and uranyl acetate staining, dehydration in alcohols and embedding in Taab 812 Resin. Sections were cut with a Leica ultracut microtome, picked up on formvar/carbon-coated copper slot grids, and stained with 0.2% Lead Citrate. Sections were then imaged under a Phillips Tecnai BioTwin Spirit transmission electron microscope.

Human Heart Biopsy Samples

Hearts were obtained under a protocol approved by the University of Utah's institutional review board (IRB).

Echocardiography

Murine transthoracic echocardiography was performed on conscious mice using a Vevo 3100 high resolution microultrasound system (Visualsonics, Inc., Toronto, Canada), as previously described[1]. Mice were acclimated to the procedure at least three independent times before data collection. Echocardiograghic images were acquired with the mouse held in a prone position with the transducer facing upward onto the chest wall.

Blood Pressure Measurement

Systemic blood pressure was measured using a tail cuff with a CODA noninvasive blood pressure system (Kent Scientific). Awake mice were acclimated to the system for 10 minutes on a warm plate and measurements were done without the use of anesthesia. At least six blood pressure measurements were obtained for each mouse and measurements for each genotype was then averaged.

Statistical Analysis

GraphPad Prism version 9 and 10 was used for statistical analysis included in the main and supplementary figures. Statistical significance was calculated using unpaired, two-tailed Student's *t* test or one-way ANOVA with Sidak multiple comparisons test for post hoc pairwise comparisons. When two independent factors were used in the experimental design, statistical significance was calculated using a two-way ANOVA with Sidak multiple comparisons test. Data was evaluated for normality before parametric statistics were applied using the Shaprio-Wilk test. For groups of unequal variances, data was reevaluated by *t* test or ANOVA with Welch correction. For comparisons for two groups with non-normal distribution, the Mann-Whitney test was used. For comparisons of more than two groups with non-normal distribution, the Kruskal-Wallis test was used. Survival curve analysis was performed using the Log-rank test. *P* values were considered statistically significant if the *P* value was <0.05. For all figures, * indicates $p < 0.05$ unless otherwise indicated. Error bars represent SD unless otherwise indicated. No statistical methods were used to pre-determine the sample size. All experimental results represent observations from at least 3 biological replicates, except for the RNA-Seq and lipidomics, which were measured from 2 biological replicates.

Figure S1

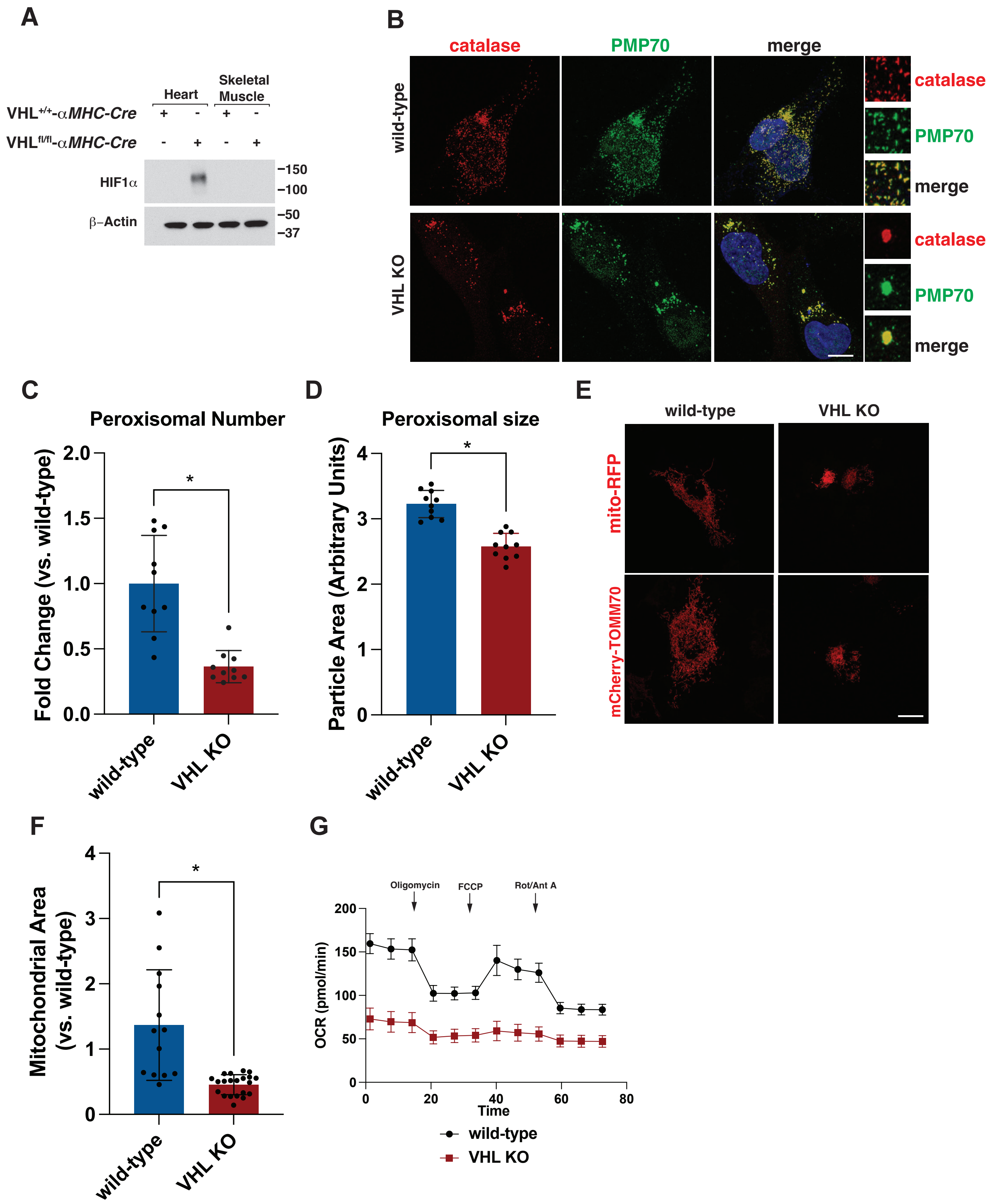

A
Heart
Skeletal Muscle
VHL+/+-αMHC-Cre
VHLfl/fl-αMHC-Cre
HIF1α
β-Actin
150
100
50
37
B
catalase
PMP70
merge
wild-type
VHL KO
C
Peroxisomal Number
Fold Change (vs. wild-type)
2.0
1.5
1.0
0.5
0.0
wild-type
VHL KO
D
Peroxisomal size
Particle Area (Arbitrary Units)
4
3
2
1
0
E
wild-type
VHL KO
mito-RFP
mCherry-TOMM70
F
Mitochondrial Area (vs. wild-type)
G
Oligomycin
FCCP
Rot/Ant A
OCR (pmol/min)
200
150
100
50
0
20
40
60
80
Time
wild-type
VHL KO

Figure S2

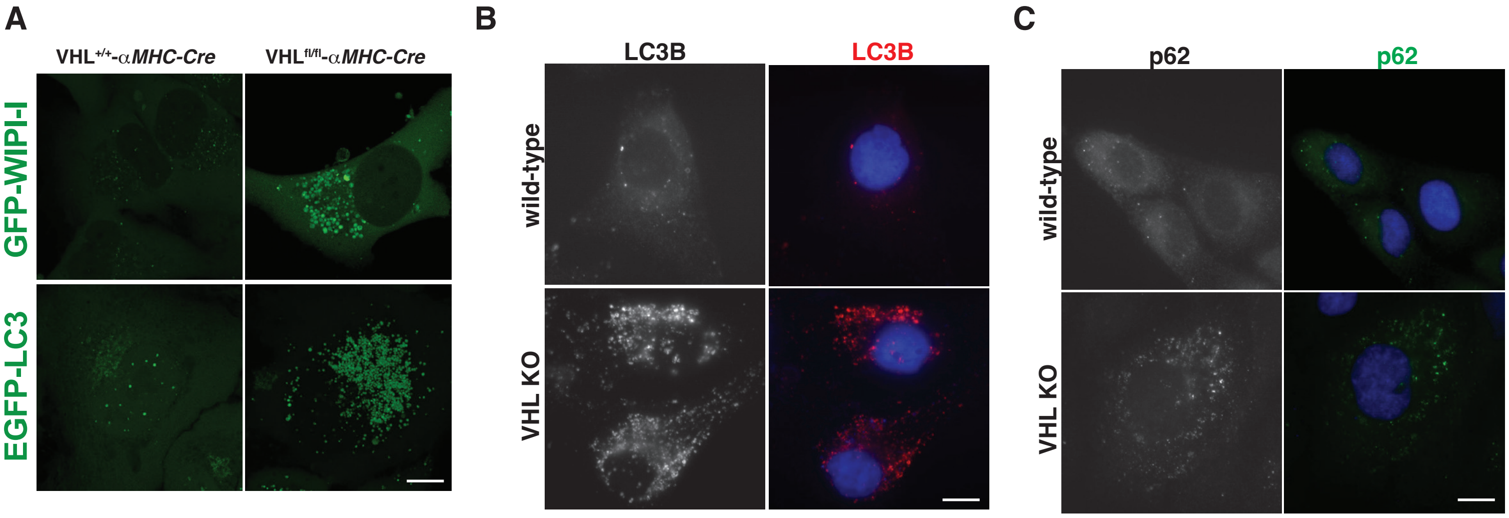
A
VHL+/+-αMHC-Cre
VHLfl/fl-αMHC-Cre
GFP-WIPI-I
EGFP-LC3
B
LC3B
LC3B
wild-type
VHL KO
C
p62
p62
wild-type
VHL KO

Figure S3

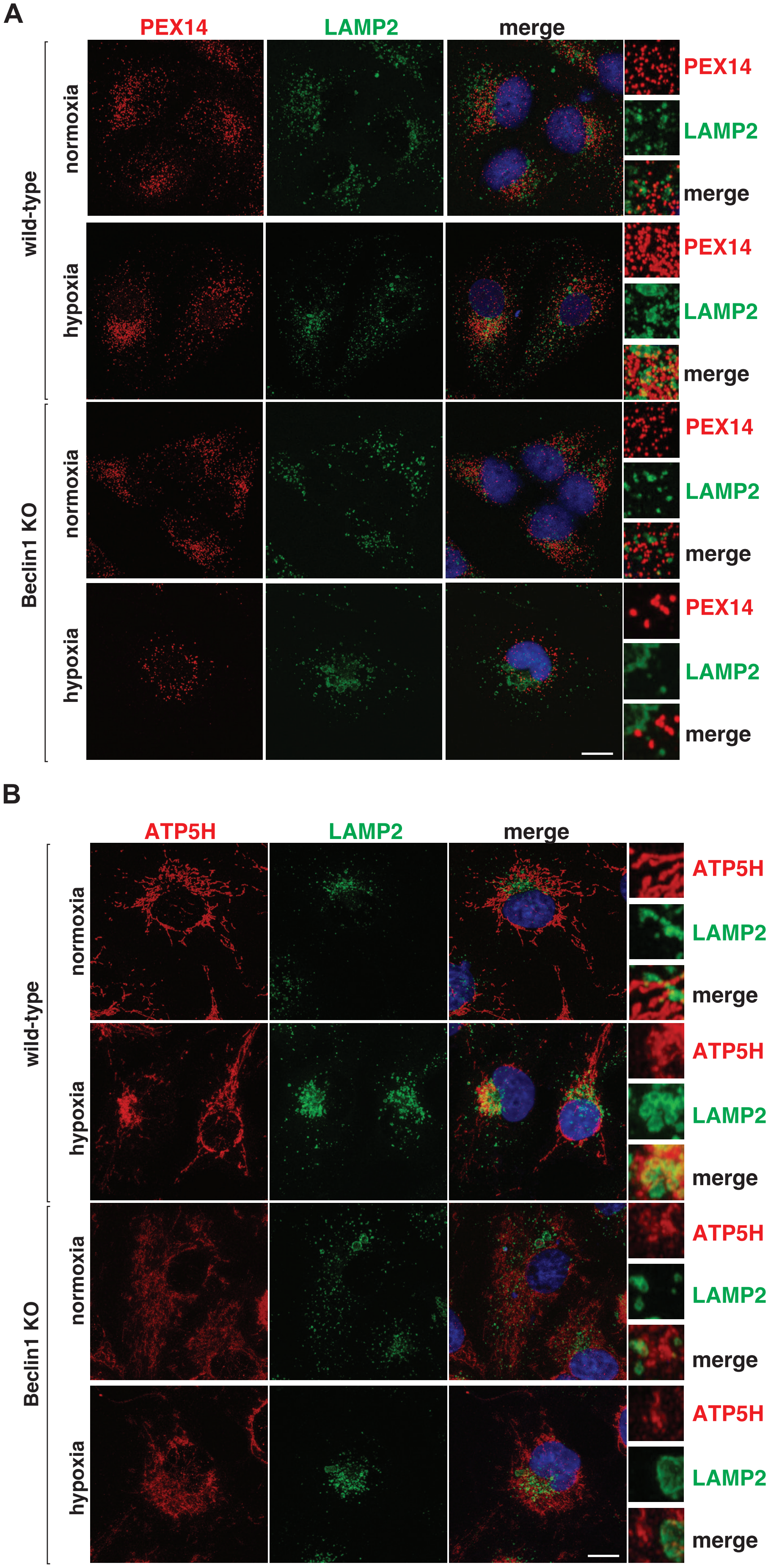
A
PEX14
LAMP2
merge
wild-type
normoxia
hypoxia
Beclin1 KO
normoxia
hypoxia
PEX14
LAMP2
merge
B
ATP5H
LAMP2
merge
wild-type
normoxia
hypoxia
Beclin1 KO
normoxia
hypoxia
ATP5H
LAMP2
merge

Figure S4

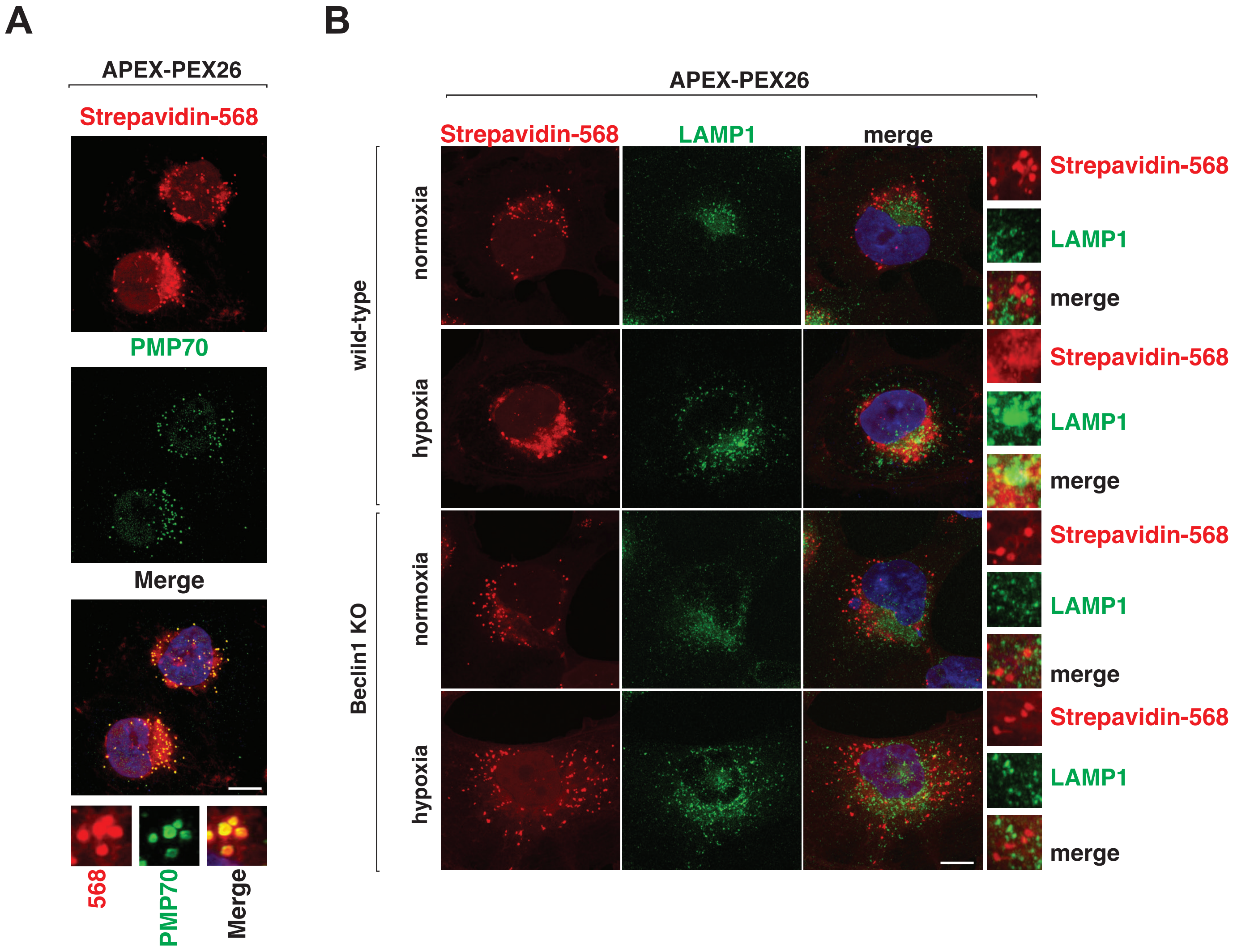
A
APEX-PEX26
Strepavidin-568
PMP70
Merge
568
PMP70
Merge
B
APEX-PEX26
Strepavidin-568
LAMP1
merge
wild-type
normoxia
hypoxia
Beclin1 KO
normoxia
hypoxia
Strepavidin-568
LAMP1
merge
Strepavidin-568
LAMP1
merge
Strepavidin-568
LAMP1
merge
Strepavidin-568
LAMP1
merge

Supplementary Figure 5

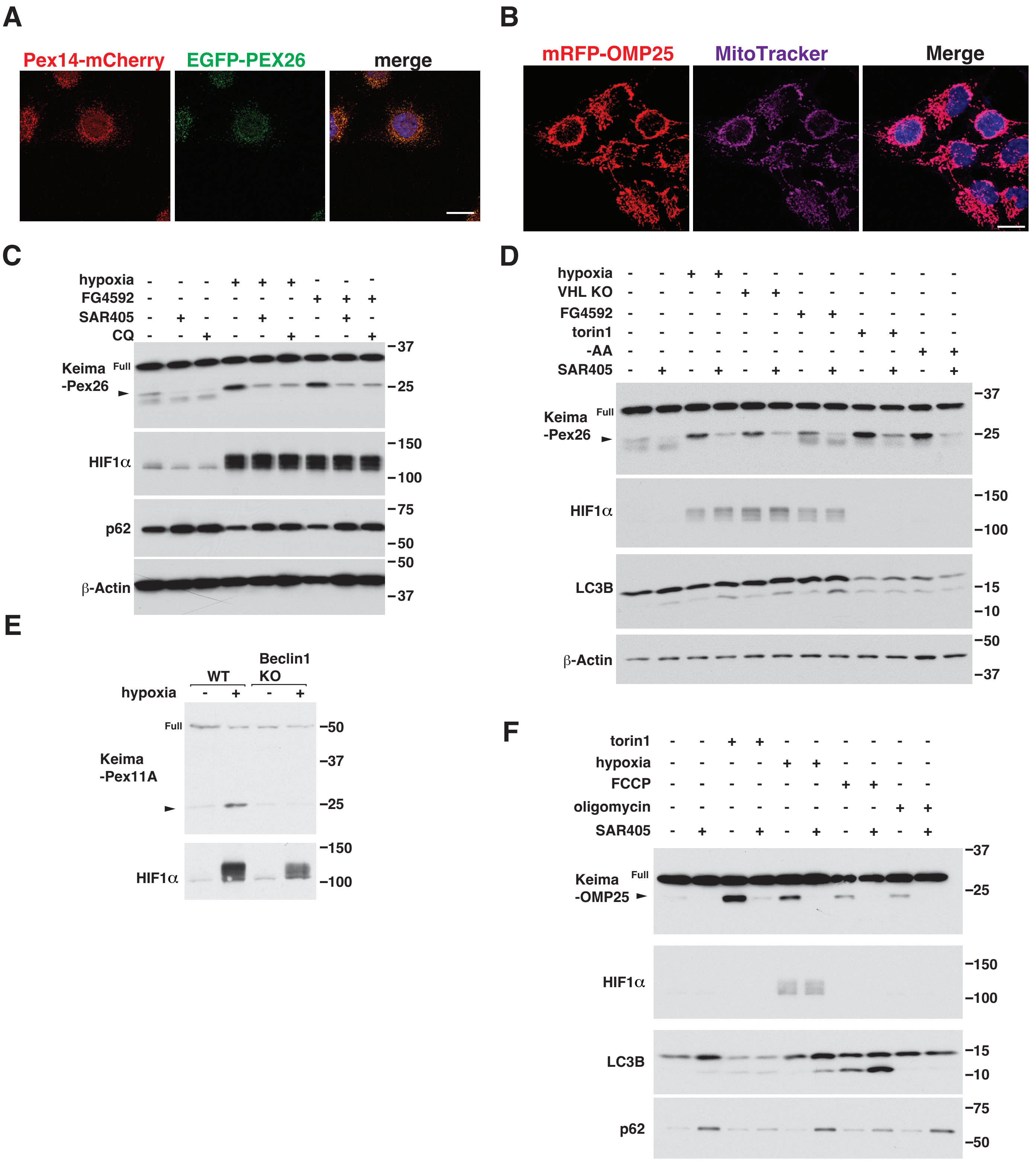

Figure S6

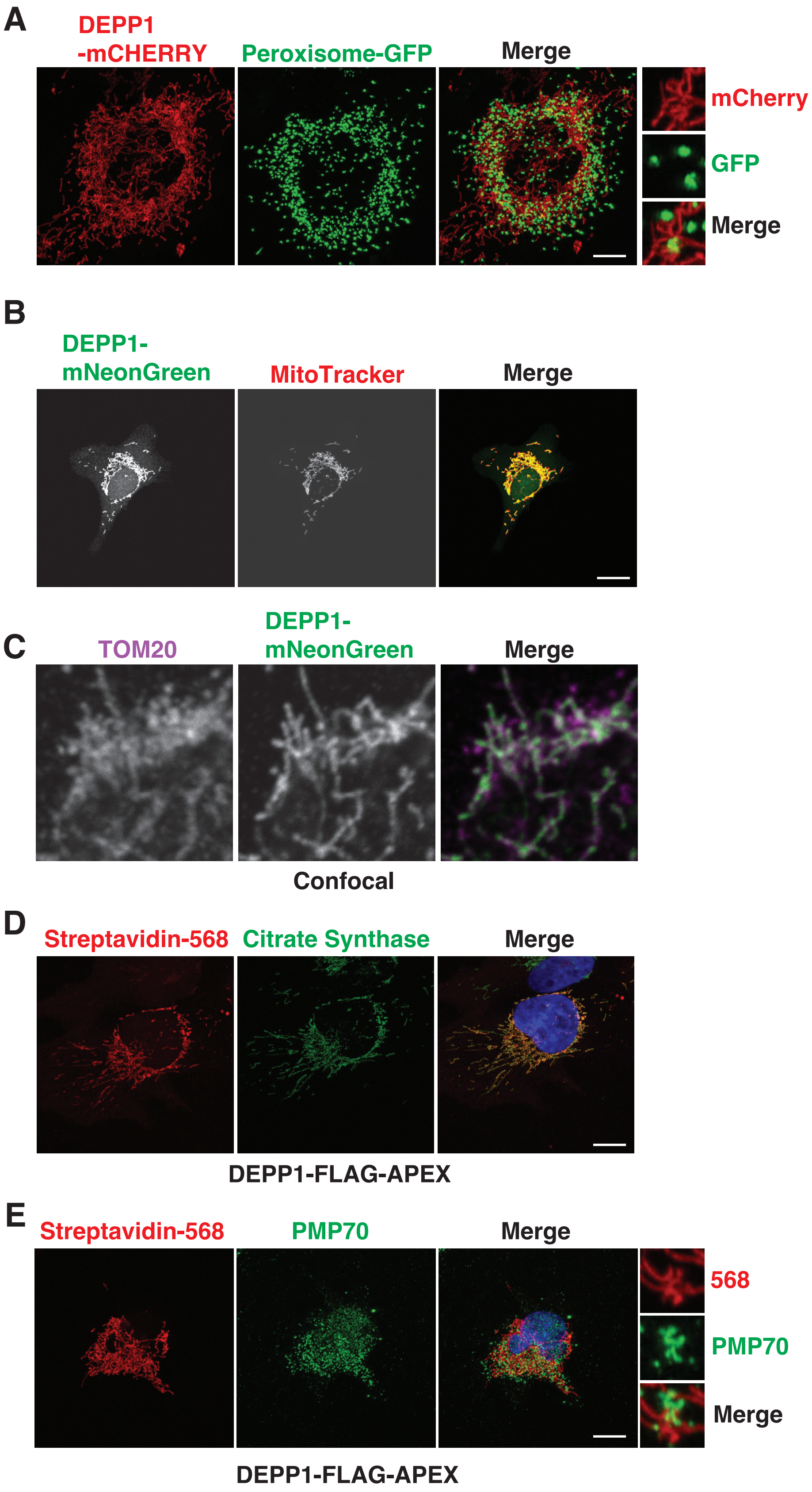
A
DEPP1
-mCHERRY
Peroxisome-GFP
Merge
mCherry
GFP
Merge
B
DEPP1-
mNeonGreen
MitoTracker
Merge
C
TOM20
DEPP1-
mNeonGreen
Merge
Confocal
D
Streptavidin-568
Citrate Synthase
Merge
DEPP1-FLAG-APEX
E
Streptavidin-568
PMP70
Merge
568
PMP70
Merge
DEPP1-FLAG-APEX

Figure S7

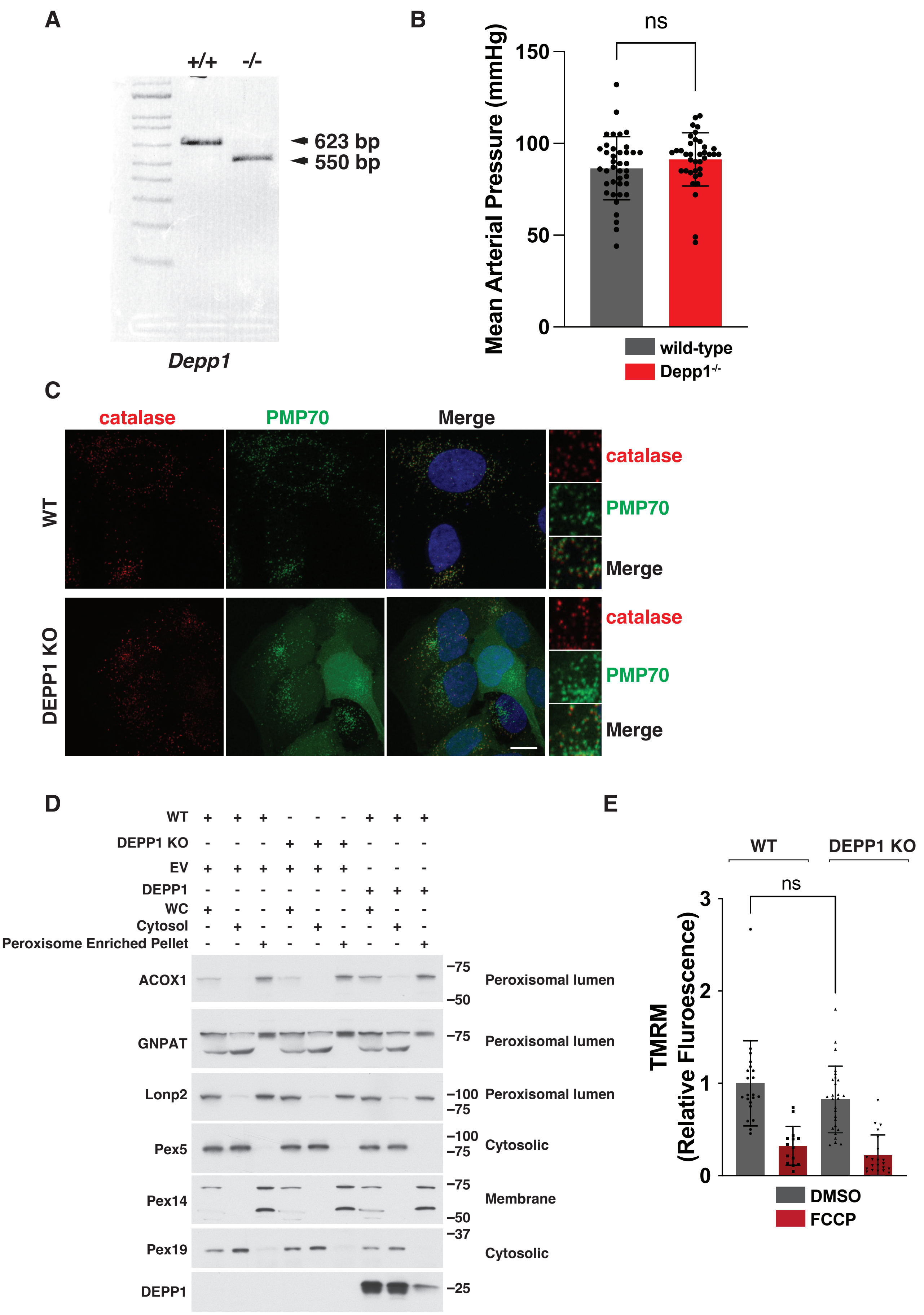

A
+/+
-/-
623 bp
550 bp
Depp1
B
ns
Mean Arterial Pressure (mmHg)
150
100
50
0
wild-type
Depp1-/-
C
catalase
PMP70
Merge
WT
DEPP1 KO
D
WT
DEPP1 KO
EV
DEPP1
WC
Cytosol
Peroxisome Enriched Pellet
ACOX1
GNPAT
Lonp2
Pex5
Pex14
Pex19
DEPP1
Peroxisomal lumen
Peroxisomal lumen
Peroxisomal lumen
Cytosolic
Membrane
Cytosolic
–75
–50
–100
–37
–25
E
WT
DEPP1 KO
ns
TMRM (Relative Fluroescence)
3
2
1
0
DMSO
FCCP

Figure S8

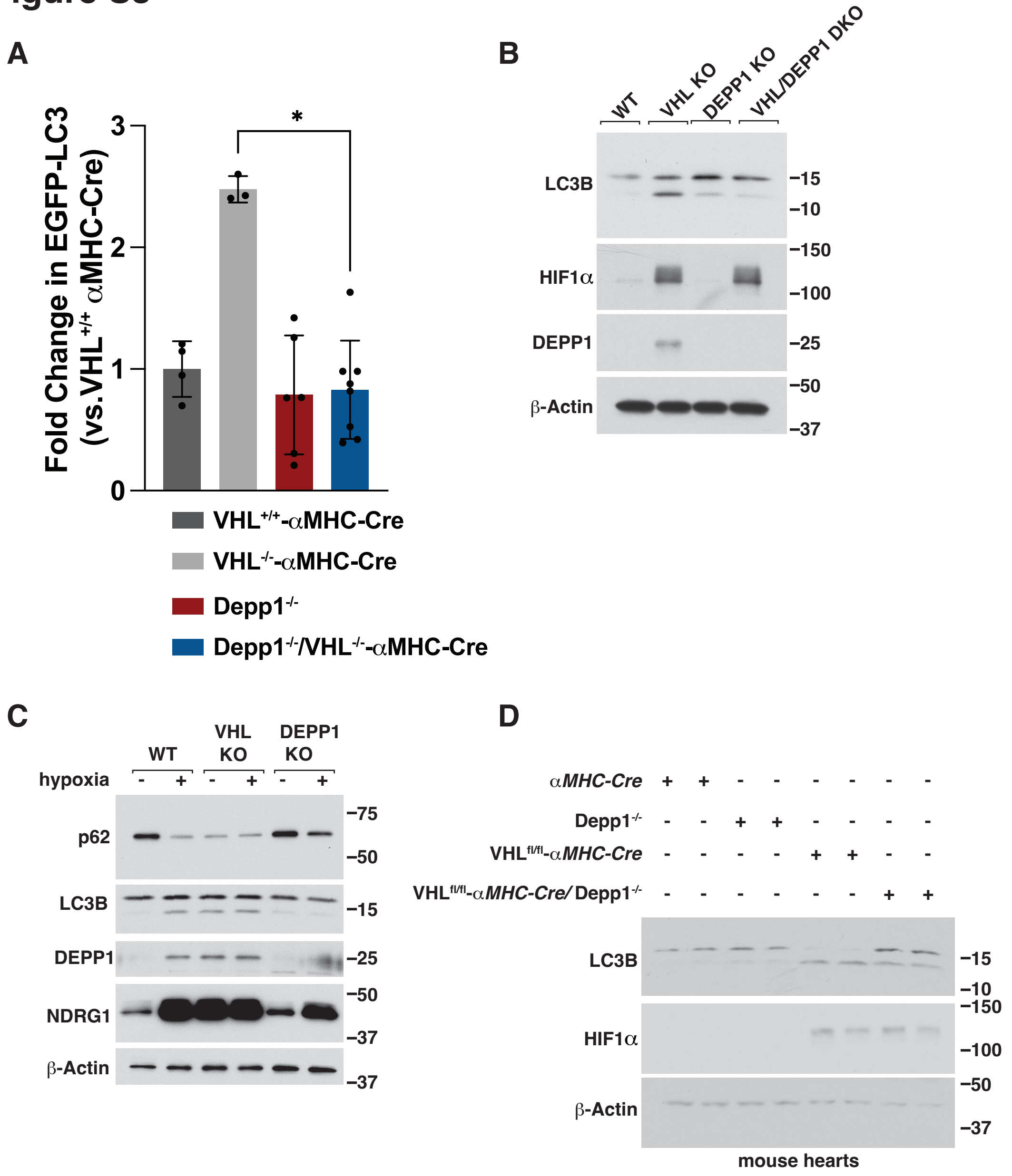
A
Fold Change in EGFP-LC3 (vs.VHL+/+ αMHC-Cre)
*
VHL+/+-αMHC-Cre
VHL-/--αMHC-Cre
Depp1-/-
Depp1-/-/VHL-/--αMHC-Cre
B
WT
VHL KO
DEPP1 KO
VHL/DEPP1 DKO
LC3B
HIF1α
DEPP1
β-Actin
C
WT
VHL KO
DEPP1 KO
hypoxia
p62
LC3B
DEPP1
NDRG1
β-Actin
D
αMHC-Cre
Depp1-/-
VHLfl/fl-αMHC-Cre
VHLfl/fl-αMHC-Cre/ Depp1-/-
LC3B
HIF1α
β-Actin
mouse hearts

Figure S9

A

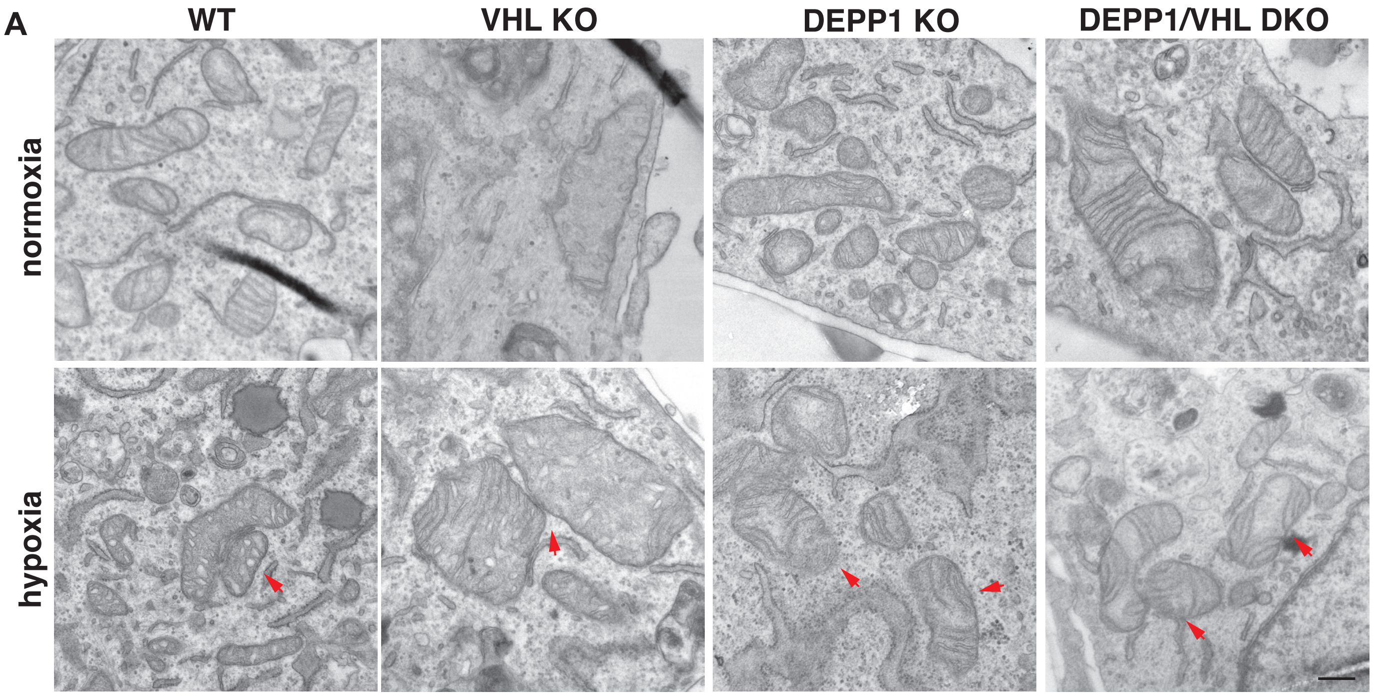

WT
VHL KO
DEPP1 KO
DEPP1/VHL DKO
normoxia
hypoxia

B

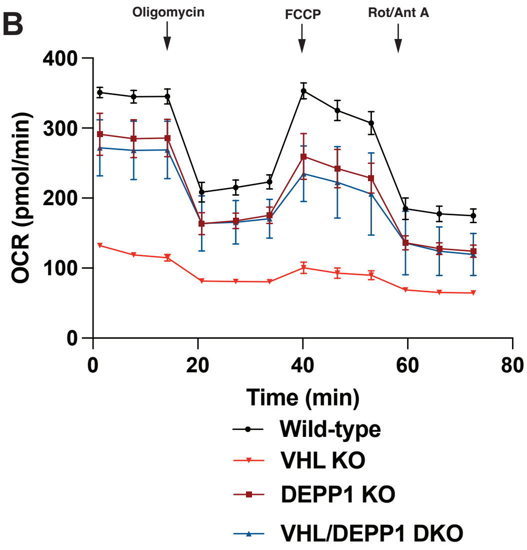

Oligomycin
FCCP
Rot/Ant A
OCR (pmol/min)
400
300
200
100
0
0
20
40
60
80
Time (min)
Wild-type
VHL KO
DEPP1 KO
VHL/DEPP1 DKO

Figure S10

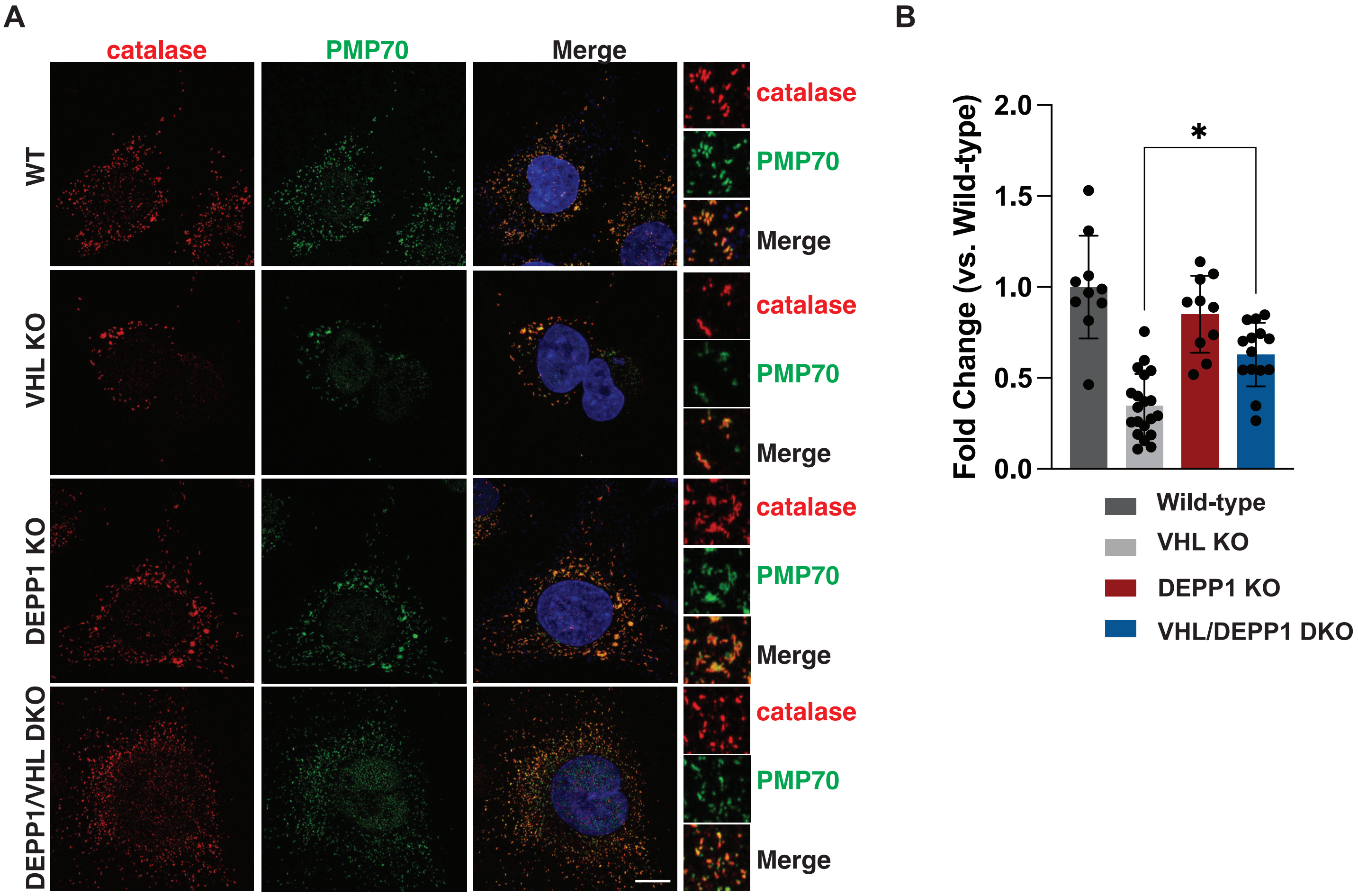

A
catalase
PMP70
Merge
WT
VHL KO
DEPP1 KO
DEPP1/VHL DKO
catalase
PMP70
Merge
B
Fold Change (vs. Wild-type)
2.0
1.5
1.0
0.5
0.0
*
Wild-type
VHL KO
DEPP1 KO
VHL/DEPP1 DKO

Figure S11

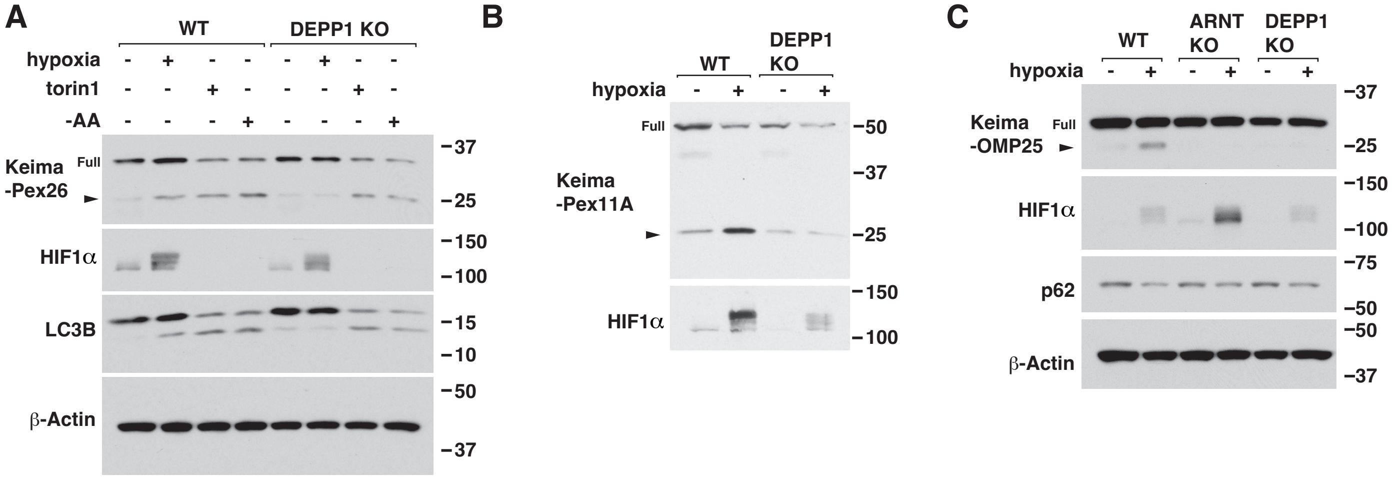
A
WT
DEPP1 KO
hypoxia
torin1
-AA
Keima
-Pex26
Full
HIF1α
LC3B
β-Actin
37
25
150
100
15
10
50
37
B
WT
DEPP1 KO
hypoxia
Full
Keima
-Pex11A
HIF1α
50
37
25
150
100
C
WT
ARNT KO
DEPP1 KO
hypoxia
Keima
-OMP25
Full
HIF1α
p62
β-Actin
37
25
150
100
75
50
50
37

Figure S12

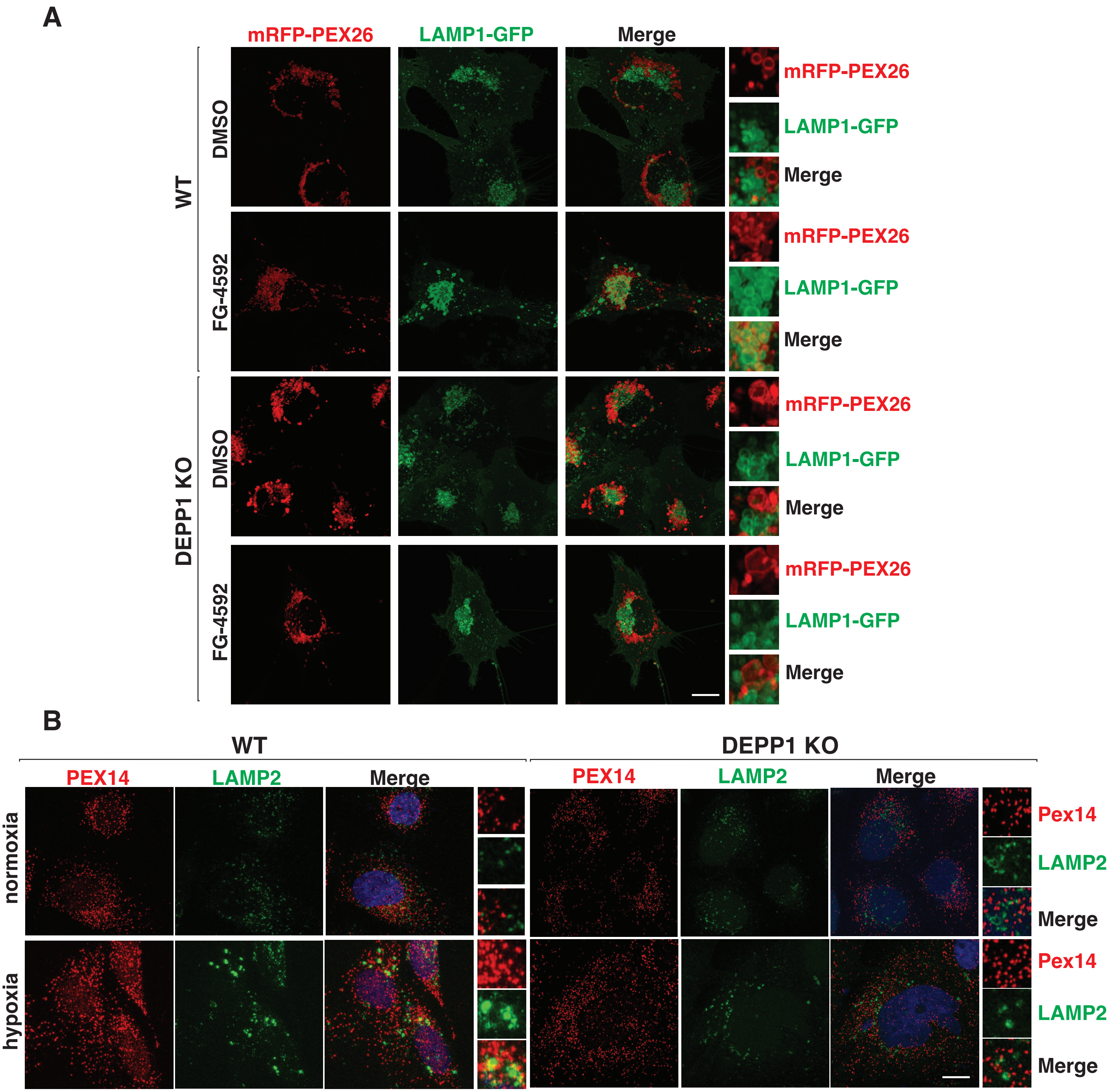

A
mRFP-PEX26
LAMP1-GFP
Merge
WT
DMSO
FG-4592
DEPP1 KO
DMSO
FG-4592
mRFP-PEX26
LAMP1-GFP
Merge
B
WT
DEPP1 KO
PEX14
LAMP2
Merge
normoxia
hypoxia
Pex14
LAMP2
Merge

Figure S13

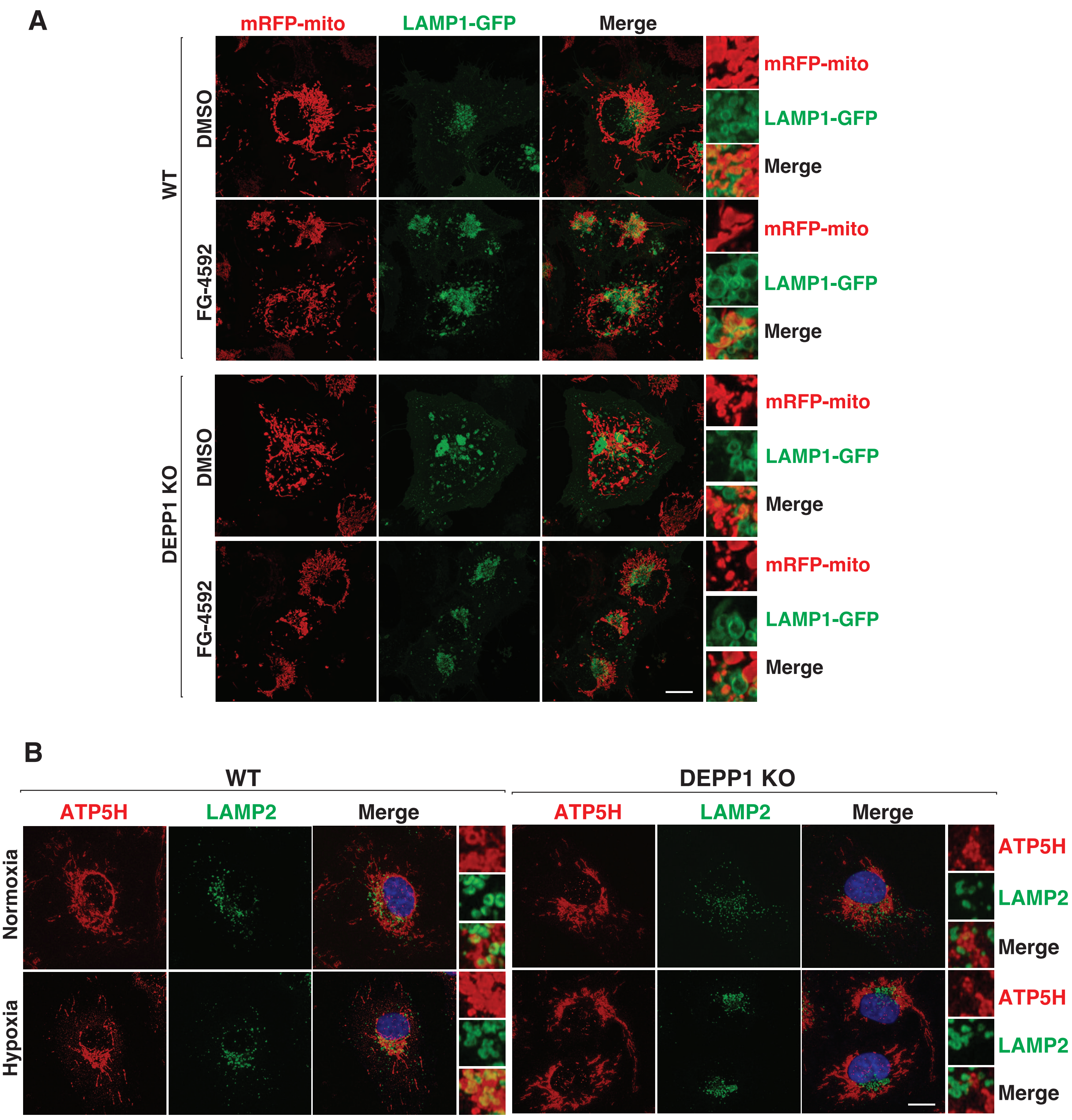
A
mRFP-mito
LAMP1-GFP
Merge
WT
DMSO
FG-4592
DEPP1 KO
mRFP-mito
LAMP1-GFP
Merge
B
WT
DEPP1 KO
ATP5H
LAMP2
Merge
Normoxia
Hypoxia

Figure S14

A

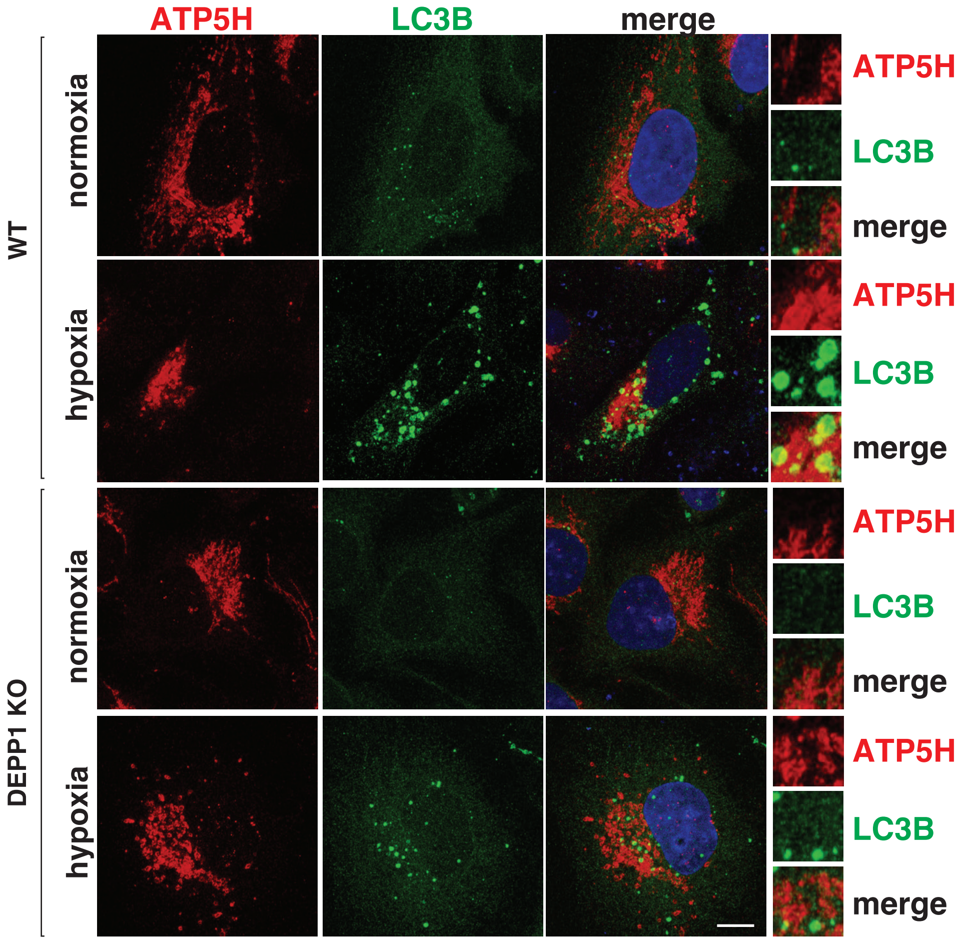

ATP5H
LC3B
merge
WT
normoxia
hypoxia
DEPP1 KO
normoxia
hypoxia
ATP5H
LC3B
merge
ATP5H
LC3B
merge
ATP5H
LC3B
merge
ATP5H
LC3B
merge

Figure S15

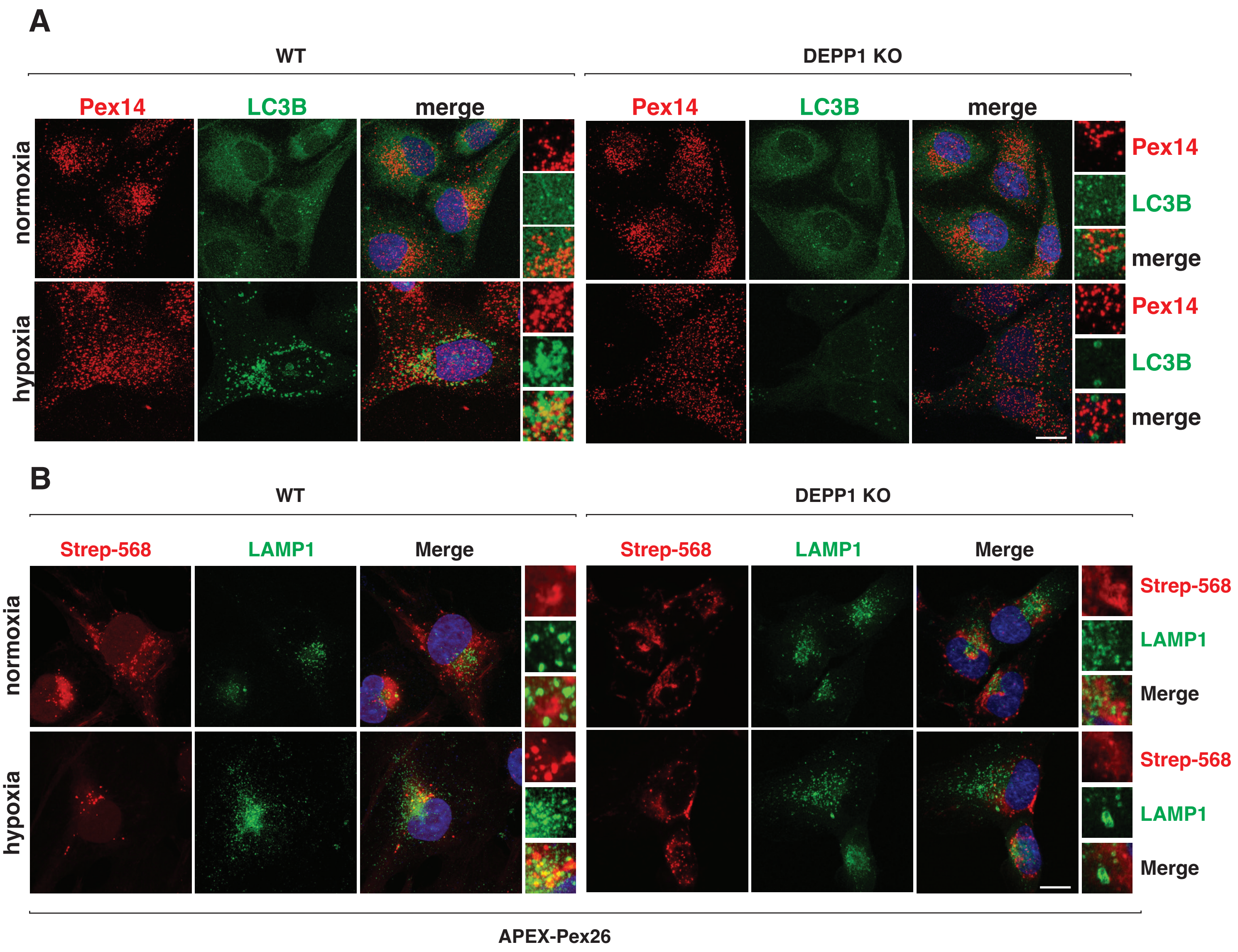

A
WT
DEPP1 KO
Pex14
LC3B
merge
normoxia
hypoxia
Pex14
LC3B
merge
B
WT
DEPP1 KO
Strep-568
LAMP1
Merge
normoxia
hypoxia
Strep-568
LAMP1
Merge
APEX-Pex26

Figure S16

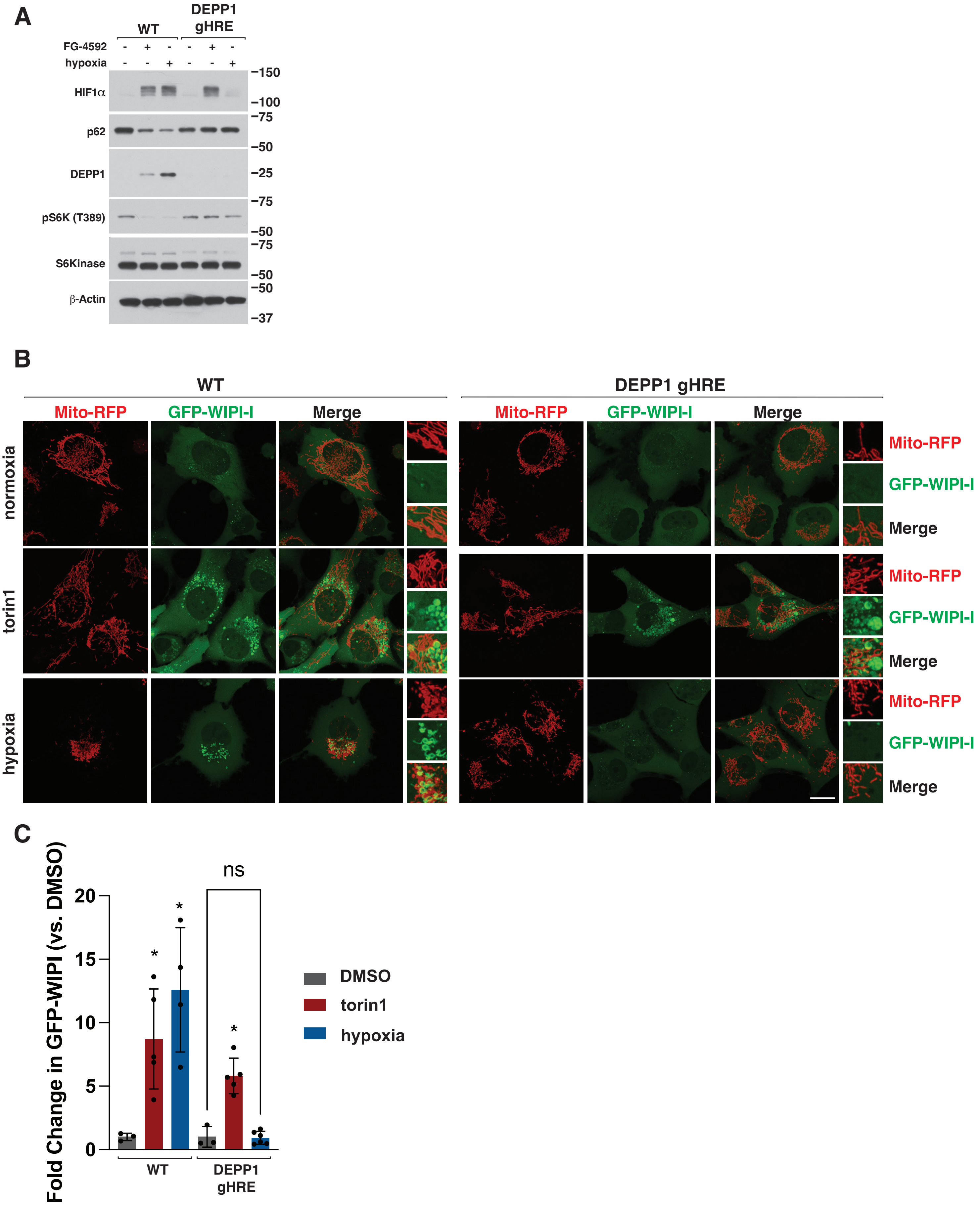
A
WT
DEPP1 gHRE
FG-4592
hypoxia
HIF1α
p62
DEPP1
pS6K (T389)
S6Kinase
β-Actin
150
100
75
50
25
75
50
75
50
50
37
B
WT
DEPP1 gHRE
Mito-RFP
GFP-WIPI-I
Merge
normoxia
torin1
hypoxia
C
Fold Change in GFP-WIPI (vs. DMSO)
ns
20
15
10
5
0
WT
DEPP1 gHRE
DMSO
torin1
hypoxia

Figure S17

A

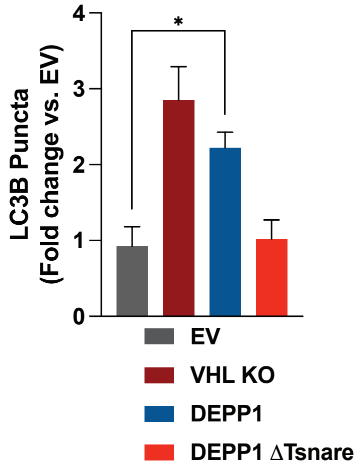

B

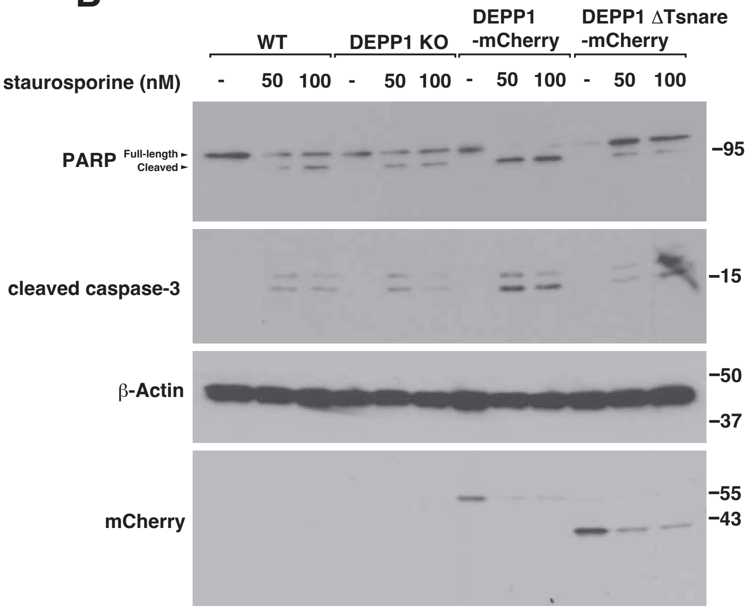

Figure S18

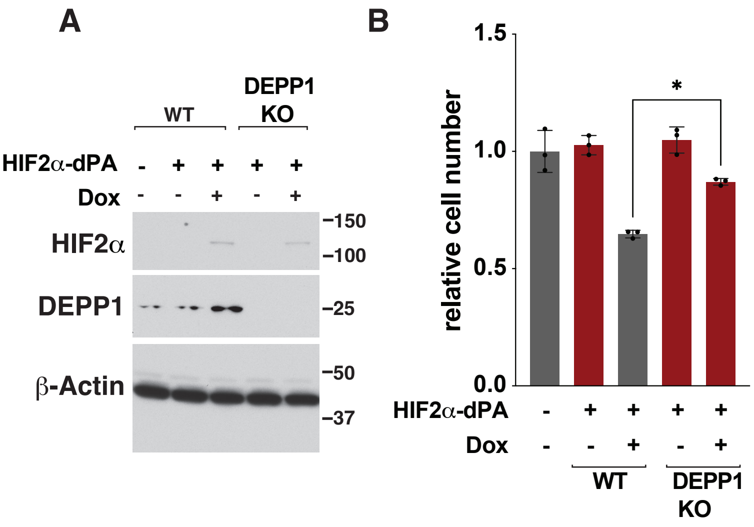

A
B
WT
DEPP1 KO
HIF2α-dPA
Dox
HIF2α
DEPP1
β-Actin
–150
–100
–25
–50
–37
1.5
1.0
0.5
0.0
relative cell number
*
HIF2α-dPA
Dox
WT
DEPP1 KO

Figure S19

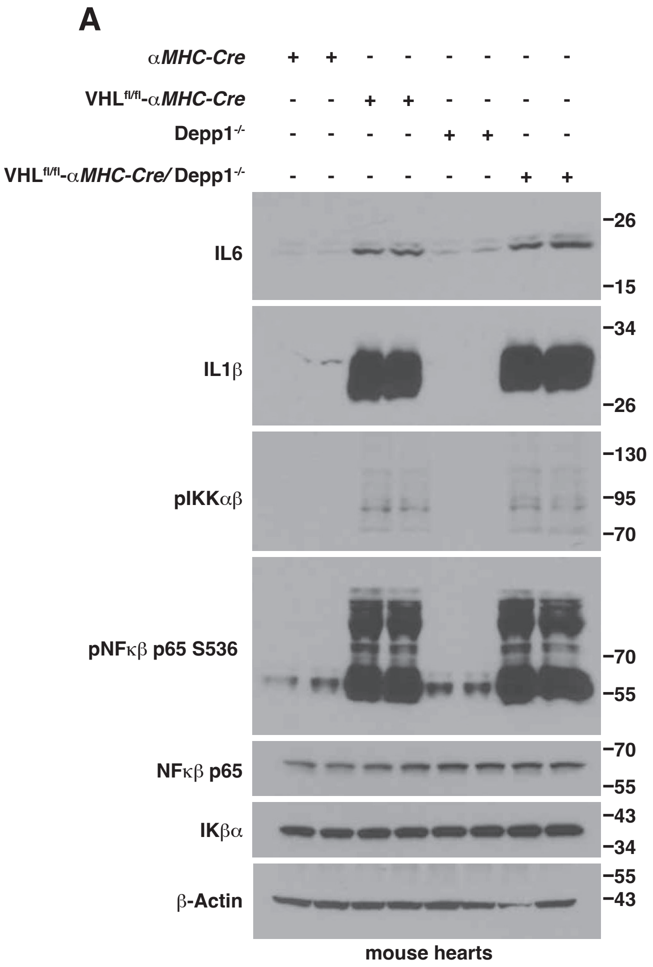
A
αMHC-Cre
VHLfl/fl-αMHC-Cre
Depp1-/-
VHLfl/fl-αMHC-Cre/ Depp1-/-
IL6
IL1β
pIKKαβ
pNFκβ p65 S536
NFκβ p65
IKβα
β-Actin
mouse hearts
26
15
34
26
130
95
70
70
55
70
55
43
34
55
43

Figure S20

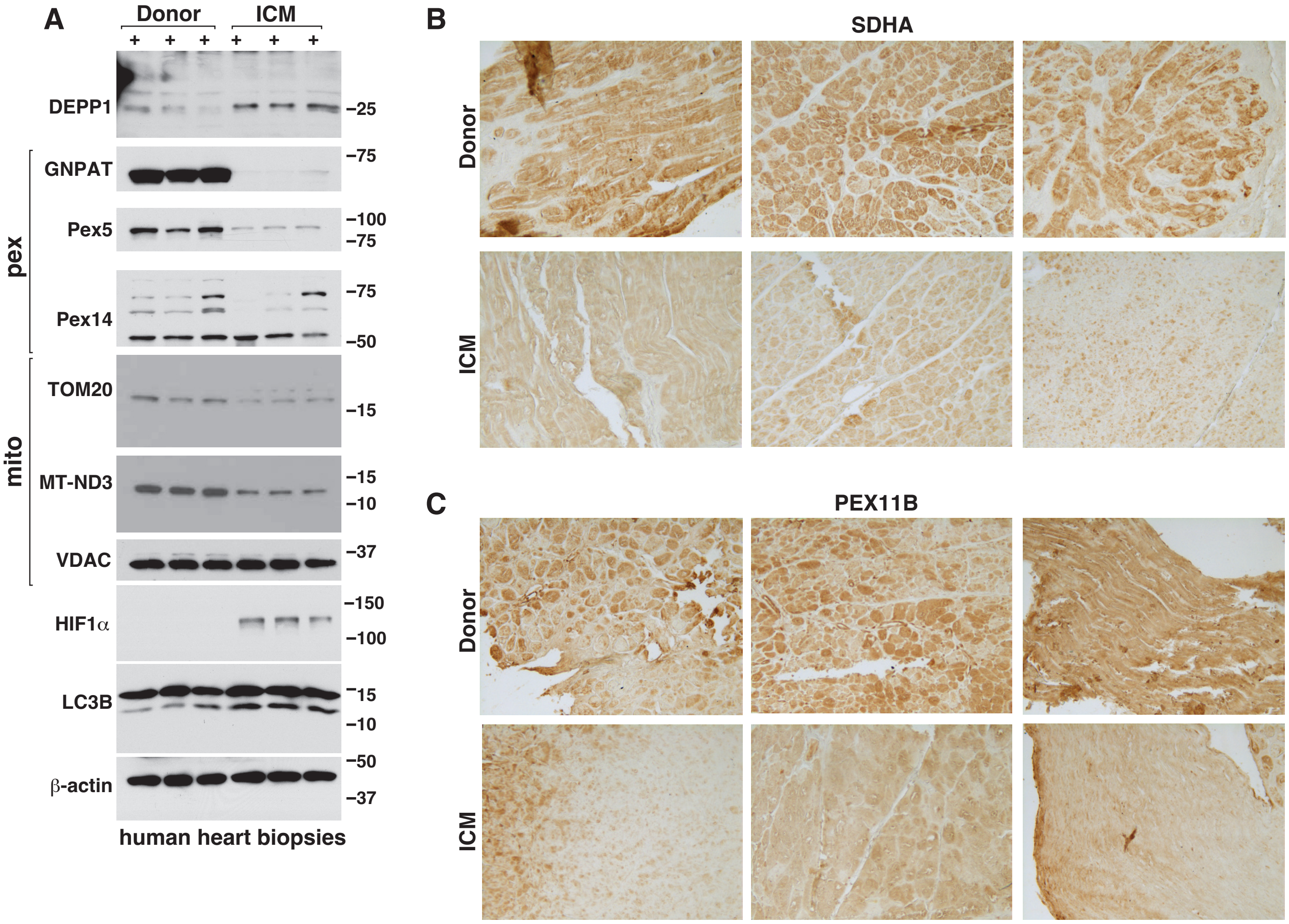

A
Donor
ICM
+ + + + + +
DEPP1
–25
pex
GNPAT
–75
Pex5
–100
–75
Pex14
–75
–50
mito
TOM20
–15
MT-ND3
–15
–10
VDAC
–37
HIF1α
–150
–100
LC3B
–15
–10
β-actin
–50
–37
human heart biopsies
B
SDHA
Donor
ICM
C
PEX11B
Donor
ICM

Supplementary Figure 1: VHL loss disrupts mitochondrial and peroxisomal morphology. (A) Immunoblot analysis of heart and skeletal muscle lysates from mice with the indicated genotypes (male, age 5 weeks). **(B)** Confocal microscopy of wild-type and *VHL* knockout mouse neonatal cardiomyocytes. Scale bar indicates 10 □m. **(C)** Peroxisomal number, as determined by quantification of PMP70 immunofluorescence using Fiji analysis software, in wild-type and *VHL* knockout mouse neonatal cardiomyocytes. Data shows mean-/+SD, n=10, *$p<0.05$ by student's t-test. **(D)** Peroxisomal size, as determined by PMP70 particle area using Fiji analysis software, in wild-type and *VHL* knockout mouse neonatal cardiomyocytes. Data shows mean -/+SD, n=10, *$p<0.05$ by student's t-test. **(E)** Live cell confocal microscopy of wild-type and *VHL* knockout mouse neonatal cardiomyocytes stably expressing mito-RFP or mCherry-TOM70. Scale bar indicates 10 μm. **(F)** Mitochondrial area, as determined by quantification of tetramethylrhodamine methyl ester (TMRM) staining using live cell confocal microscopy and Fiji analysis software, in wild-type and *VHL* knockout mouse neonatal cardiomyocytes. Data shows mean -/+SD, n=10, *$p<0.05$ by student's t-test. **(G)** Seahorse analysis of oxygen consumption rate (OCR) in wild-type and *VHL* knockout mouse neonatal cardiomyocytes, n>3.

Supplementary Figure 2: VHL loss induces autophagy in cardiomyocytes. (A) Live cell confocal microscopy of cardiomyocytes derived from hearts of mice with the indicated genotypes and then engineered to transiently express GFP-WIPI-I or EGFP-LC3. **(B-C)** Fluorescent microscopy of wild-type and VHL knockout AC16 human cardiomyocytes. Scale bar indicates 10 μm.

Supplementary Figure 3: Hypoxia induces Beclin1-dependent peroxisome and mitochondria co-localization with LAMP1-positive lysosomes. (A-B) Confocal microscopy of wild-type and Beclin1 knockout AC16 human cardiomyocytes grown at hypoxia (1% O_2) or normoxia for 18 hours. Pex14 **(A)** is a marker for peroxisomes and ATP5H **(B)** is a marker of mitochondria. Scale bar indicates 10 μm.

Supplementary Figure 4: Hypoxia induces Beclin1-dependent Pex26 biotinylation of LAMP1-positive lysosomes. (A) AC16 human cardiomyocytes stably expressing APEX-Pex26 were treated with Biotin-Tryamide for 30 minutes followed by H_2O_2 for 1 minutes (to enable biotinylation) and visualized with Streptavidin-568. **(B)** Wild-type and Beclin1 knockout AC16 human cardiomyocytes stably expressing APEX-Pex26 grown at hypoxia (1% O_2) or normoxia for 18 hours. After 18 hours, cells were treated with Biotin-Tryamide for 30 minutes followed by H_2O_2 for 1 minute (to enable biotinylation) and visualized with Streptavidin-568. Scale bar indicates 10 μm.

Supplementary Figure 5: Validation of Keima-PEX and Keima-OMP25 reporters. (A-B) Live cell confocal microscopy of AC16 human cardiomyocytes stably expressing Pex14-mCherry and EGFP-Pex26 **(A)** or mRFP-OMP25 and treated with MitoTracker DeepRed **(B)**. Cells were counterstained with Hoechst 33342. Scale bar indicates 10 □m. **(C-D)** Immunoblot analysis of wild-type or VHL knockout AC16 human cardiomyocytes stably expressing Keima-Pex26. Where indicated, cells were treated with 250 nM torin1, amino acid deprivation (-AA), 100 μM FG-4592, hypoxia (1% O_2), 1 μM SAR-405, or 30

μM chloroquine (CQ) for 18 hours. Arrow indicates liberated Keima protein. **(E)** Immunoblot analysis of wild-type or Beclin1 knockout AC16 human cardiomyocytes stably expressing Keima-Pex11A. Where indicated, cells were grown at hypoxia (1% O_2) or normoxia for 18 hours. Arrow indicates liberated Keima protein. **(F)** Immunoblot analysis of AC16 human cardiomyocytes stably expressing Keima-OMP25. Where indicated, cells were treated with 250 nM torin1, hypoxia (1% O_2), 1 μM SAR-405, 1 μM Carbonyl cyanide (4-trifluoromethoxy) phenylhydrazone (FCCP), or 1 μM Oligomycin for 18 hours. Arrow indicates liberated Keima protein.

Supplementary Figure 6: DEPP1 localizes to mitochondria not peroxisomes in cardiomyocytes. (A) Live cell confocal microscopy of mouse neonatal cardiomyocytes stably expressing DEPP1-mCherry and GFP-PTS1. Scale bar indicates 10 μm. **(B)** Live cell confocal microscopy with Airyscan of DEPP1 knockout AC16 human cardiomyocytes stably expressing DEPP1-mNeonGreen and treated with MitoTracker Deep Red. Scale bar indicates 10 μm. **(C)** Confocal microscopy of mouse neonatal cardiomyocytes stably expressing DEPP1-mNeonGreen. **(D-E)** Confocal microscopy of DEPP1 knockout AC16 human cardiomyocytes stably expressing DEPP1-Flag-APEX. Cells were treated with Biotin-Tryamide for 30 minutes followed by H_2O_2 for 1 minutes (to enable biotinylation) and visualized with Streptavidin-568. Scale bar indicates 10 μm.

Supplementary Figure 7: DEPP1 loss does not affect peroxisome protein abundance or mitochondria membrane potential. (A) PCR analysis of *Depp1* locus using genomic DNA from wild-type and *Depp1*$^{-/-}$ mice. **(B)** Mean arterial pressure (MAP) in wild-type and *Depp1*$^{-/-}$ mice (n=3 per group, data shows mean-/+SD, *p<0.05 by student's t-test). **(C)** Confocal microscopy of wild-type and DEPP1 knockout U2OS cells. Scale bar indicates 10 μm. **(D)** Immunoblot analysis of subcellular fractionation in wild-type and DEPP1 knockout U2OS cells. Where indicated, the cells stably expressed exogenous DEPP1 or empty vector (EV). **(E)** Live cell fluorescence microscopy analysis of wild-type and *Depp1* knockout mouse neonatal cardiomyocytes stained with 100 nM tetramethylrhodamine methyl ester (TMRM) for 30 minutes. Where indicated, cells were treated with 1μM Carbonyl cyanide (4-trifluoromethoxy) phenylhydrazone (FCCP) for 10 minutes. Data shows mean -/+SD, n>5, non-significant (ns) by two-way ANOVA with Sidak multiple comparisons test.

Supplementary Figure 8: DEPP1 loss reduces autophagy induction under hypoxia or upon pVHL loss in isolated cardiomyocytes and mouse hearts. (A) Confocal microscopy analysis using Fiji analysis software of EGFP-LC3 puncta from data shown in Figure 4A. Cardiomyocytes were isolated from hearts of mice of indicated genotypes stably expressing EGFP-LC3. n>3, *p<0.05 by one-way ANOVA with Sidak multiple comparisons test. **(B)** Immunoblot analysis of wild-type, *VHL* knockout, *DEPP1* knockout, and *VHL/DEPP1* double knockout U2OS cells. **(C)** Immunoblot analysis of wild-type, VHL knockout and DEPP1 knockout U2OS cells. Where indicated, cells were grown at hypoxia (1% O_2) or normoxia for 18 hours. **(D)** Immunoblot analysis of hearts from mice with the indicated genotypes (n=2 per group, male age 5 weeks).

Supplementary Figure 9: DEPP1 loss reduces mitochondrial abnormalities in cells lacking VHL. (A) Electron micrographs of wild-type, VHL knockout, DEPP1 knockout, and VHL/DEPP1 double knockout AC16 human cardiomyocytes. Where indicated, cells were grown at hypoxia (1% O_2) or normoxia for 18 hours. Red arrows indicate mitochondria. Scale bar indicates 500 nm. **(B)** Seahorse analysis of oxygen consumption rate (OCR) in wild-type, VHL knockout, DEPP1 knockout, and VHL/DEPP1 double knockout AC16 human cardiomyocytes, n=3.

Supplementary Figure 10: DEPP1 loss increases peroxisomal abundance in cells lacking VHL. (A-B) Confocal microscopy **(A)** and quantification using Fiji analysis software **(B)** of wild-type, VHL knockout, DEPP1 knockout, and VHL/DEPP1 double knockout AC16 human cardiomyocytes. Data shows mean -/+SD, n=10, *$p<0.05$ by one-way ANOVA with Sidak multiple comparisons test. Scale bar indicates 10 µm.

Supplementary Figure 11: DEPP1 is necessary for hypoxia induced peroxisome and mitochondria autophagy. (A) Immunoblot analysis of wild-type or DEPP1 knockout AC16 human cardiomyocytes stably expressing Keima-Pex26 treated with 250 nM torin1, amino acid deprivation (-AA), or hypoxia (1% O_2) for 18 hours. **(B)** Immunoblot analysis of wild-type or DEPP1 knockout AC16 human cardiomyocytes stably expressing Keima-Pex11A grown at hypoxia (1% O_2) or normoxia for 18 hours. **(C)** Immunoblot analysis of wild-type, ARNT knockout, or DEPP1 knockout AC16 human cardiomyocytes stably expressing Keima-OMP25 grown at hypoxia (1% O_2) or normoxia for 18 hours.

Supplementary Figure 12: DEPP1 is necessary for HIFα-mediated peroxisome autophagy. (A) Live cell confocal microscopy of wild-type and *Depp1* knockout mouse neonatal cardiomyocytes stably expressing mRFP-Pex26 and LAMP1-GFP. Where indicated, cells were treated with 100 µM FG-4592 for 18 hours. **(B)** Confocal microscopy of wild-type and DEPP1 knockout AC16 human cardiomyocytes. Where indicated, cells were grown at hypoxia (1% O_2) or normoxia for 18 hours. Scale bar indicates 10 µm.

Supplementary Figure 13: DEPP1 is necessary for HIFα-mediated mitochondria autophagy. (A) Live cell confocal microscopy of wild-type and *Depp1* knockout mouse neonatal cardiomyocytes stably expressing mRFP-mito and LAMP1-GFP. Where indicated, cells were treated with 100 µM FG-4592 for 18 hours. **(B)** Confocal microscopy of wild-type and DEPP1 knockout AC16 human cardiomyocytes. Where indicated, cells were grown at hypoxia (1% O_2) or normoxia for 18 hours. Scale bar indicates 10 µm.

Supplementary Figure 14: DEPP1 is necessary for hypoxia induced LC3B co-localization with mitochondria. (A) Confocal microscopy of wild-type and DEPP1 knockout AC16 human cardiomyocytes. Where indicated, cells were grown at hypoxia (1% O_2) or normoxia for 18 hours. Scale bar indicates 10 µm.

Supplementary Figure 15: DEPP1 is necessary for hypoxia induced autophagy of peroxisomes. (A) Confocal microscopy of wild-type and DEPP1 knockout AC16 human cardiomyocytes. Where indicated, cells were grown at hypoxia (1% O_2) or normoxia for 18 hours. **(B)** Wild-type and DEPP1 knockout AC16 human cardiomyocytes stably

expressing APEX-Pex26 grown at hypoxia (1% O_2) or normoxia for 18 hours. After 18 hours, cells were treated with Biotin-Tryamide for 30 minutes followed by H_2O_2 for 1 minute (to enable biotinylation) and visualized with Streptavidin-568. Scale bar indicates 20 μm.

Supplementary Figure 16: Disruption of DEPP1 hypoxia response element (HRE) inhibits hypoxia-induced autophagy. (A) Immunoblot analysis of wild-type and DEPP1 gHRE U2OS cells. Where indicated, cells were treated with 100 μM FG-4592 or hypoxia (1% O_2) for 18 hours. **(B-C)** Live cell confocal microscopy **(B)** and quantification of GFP-WIPI-I puncta using Fiji analysis software **(C)** of wild-type and DEPP1 gHRE cardiomyocytes transiently expressing Mito-RFP and GFP-WIPI-I. Where indicated, cells were treated with 250 nM Torin1 or hypoxia (1% O_2) for 18 hours. *$p<0.05$ by one-way ANOVA with Sidak multiple comparisons test, n>3, non-significant (ns). Scale bar indicates 10 μm.

Supplementary Figure 17: DEPP1 is sufficient to induce LC3B lipidation and sensitizes cells to apoptosis. (A) Quantification using Fiji analysis software of fluorescence microscopy in Figure 5B of wild-type or *VHL* knockout mouse neonatal cardiomyocytes. Where indicated, the wild-type cardiomyocytes stably expressed wild-type or ΔTsnare DEPP1 or were transduced with an empty vector (EV). Data shown mean fold change versus EV -/+SD, n>3, *$p<0.05$ by one-way ANOVA with Sidak multiple comparisons test. **(B)** Immunoblot analysis of wild-type and DEPP1 knockout AC16 human cardiomyocytes. Where indicated, wild-type cardiomyocytes stably expressed wild-type or ΔTsnare DEPP1-mCherry. Cells were treated with the indicated dose (nM) of staurosporine or DMSO for 18 hours.

Supplementary Figure 18: DEPP1 loss increases cardiomyocyte survival following chronic HIFα activation (A-B) Immunoblot (**A**) and cell survival analysis (**B**) of wild-type or DEPP1 knockout human induced pluripotent stem cell (hIPS)-derived cardiomyocytes stably expressing doxycycline-inducible non-degradable HIF2a-dPA. Where indicated, cells were treated with doxycycline for 24 hours. Data shows mean -/+SD, n=3, *$p<0.05$ by two-way ANOVA with Sidak multiple comparisons test.

Supplementary Figure 19: DEPP1 loss does not affect NFκβ activation and pro-inflammatory cytokine abundance in hearts lacking pVHL. (A) Immunoblot analysis of hearts from mice with the indicated genotypes (n=2 per group, male mice 5 weeks of age).

Supplementary Figure 20: DEPP1 in human ischemic cardiomyopathy. (A-B) Immunoblot **(A)** and immunohistochemical (20X magnification) **(B)** analysis of human cardiac tissue biopsies from healthy donor and ischemic cardiomyopathy (ICM) patients (n=3 per group). SDHA is a marker for mitochondria **(B)** and PEX11B is a marker of peroxisomes **(C)**.
