# Supplementary material for: Induction of DEPP1 by HIF Mediates Multiple Hallmarks of Ischemic Cardiomyopathy
Source: Circulation. 2024 Jun 17;150(10):770–86. doi: 10.1161/CIRCULATIONAHA.123.066628 (PMC11361356; doi:10.1161/CIRCULATIONAHA.123.066628)

Full Unedited gel for Figure 1A

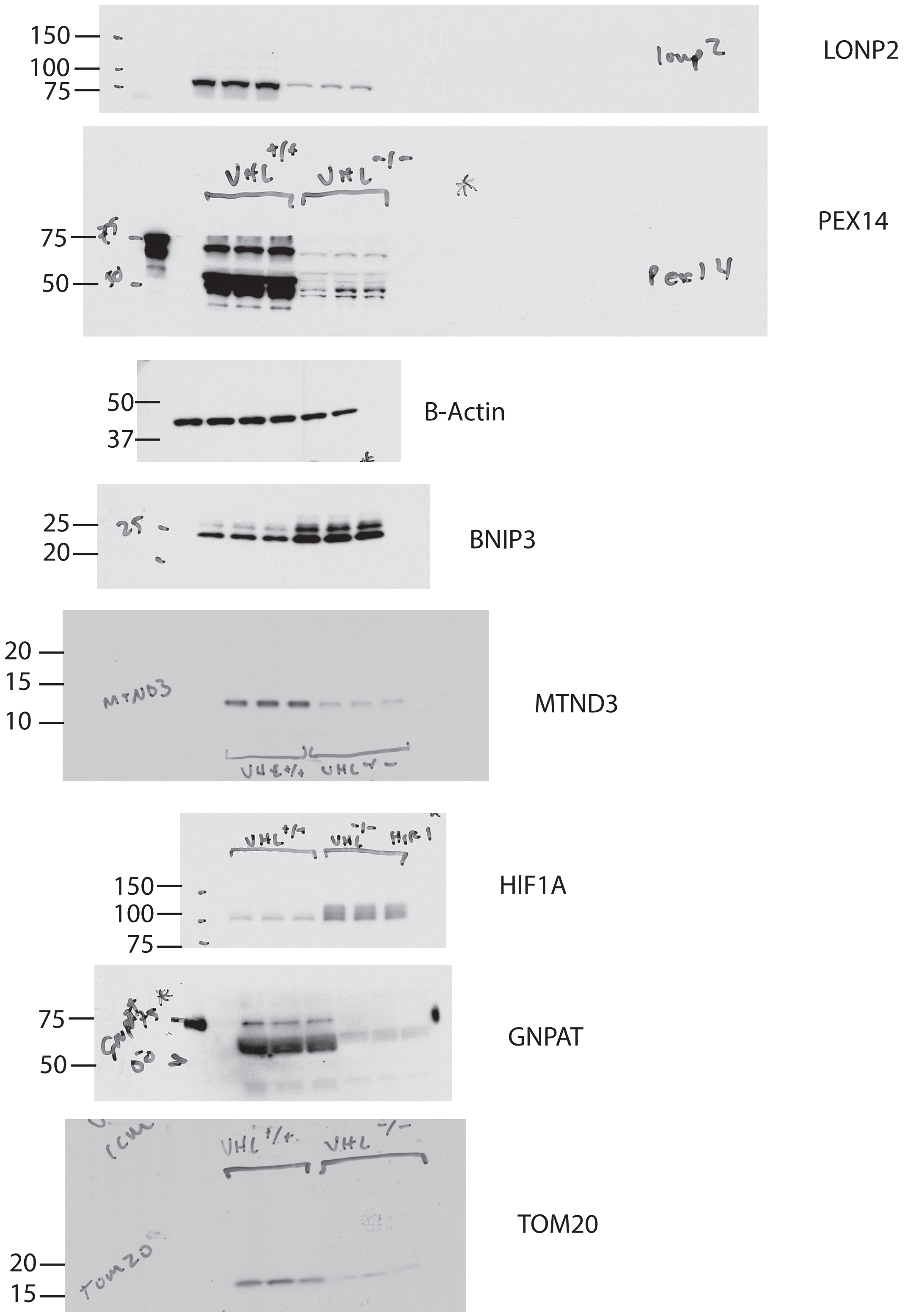

Full Unedited gel for Figure 1D

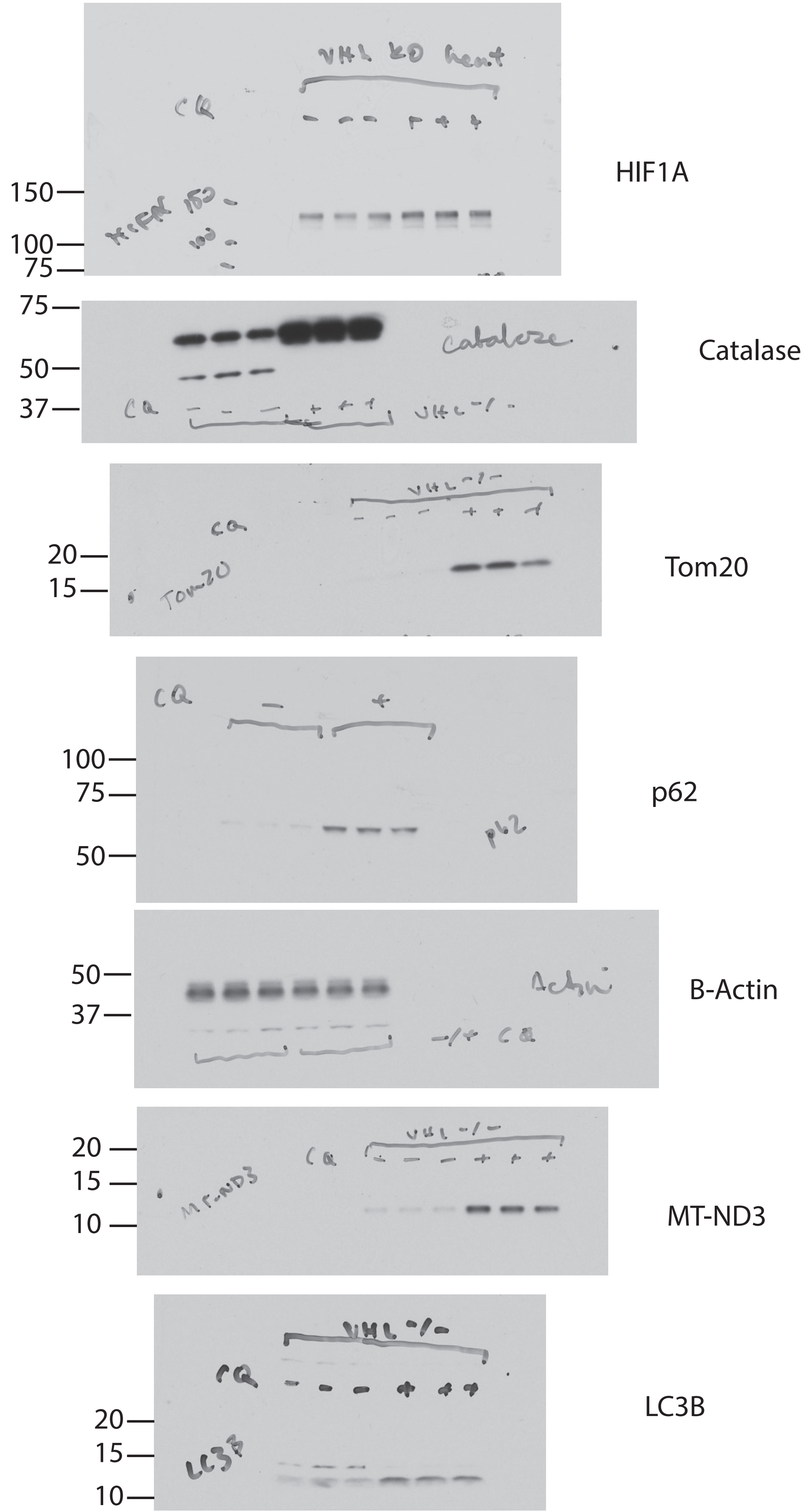

Full Unedited gel for Figure 1E

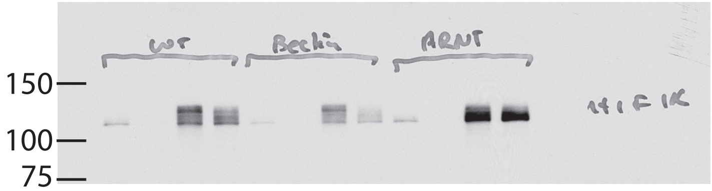

HIF1A

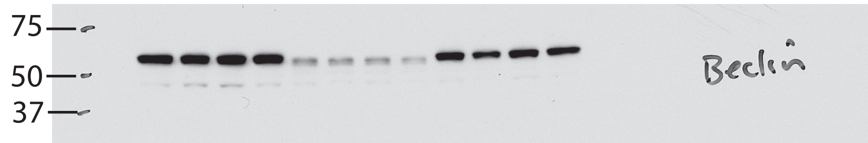

Beclin1

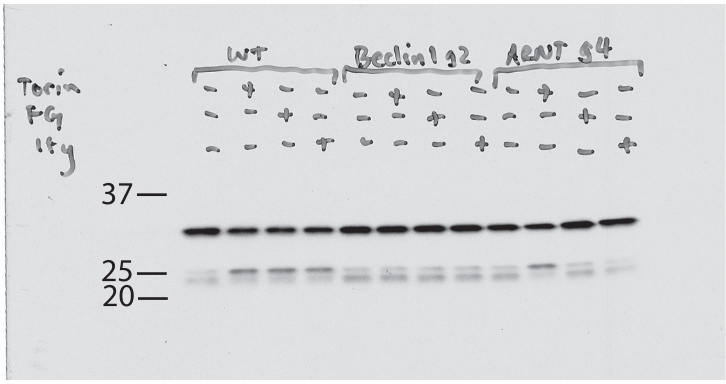

Keima

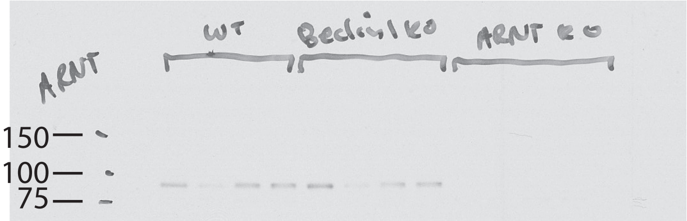

ARNT (exposure with ladder)

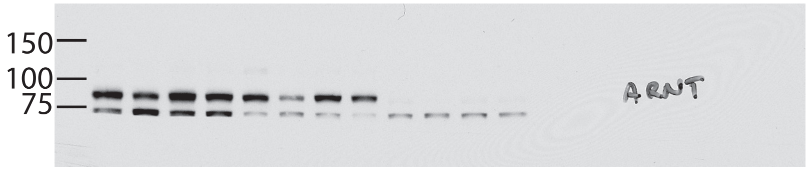

ARNT (exposure in Figure)

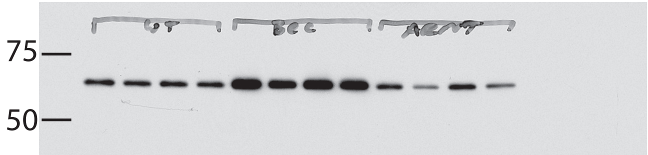

p62

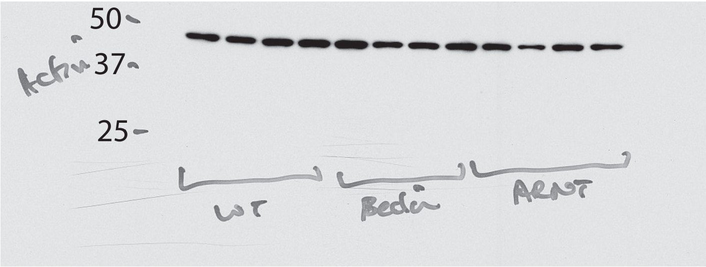

B-Actin

Full unedited gel for Figure 1F

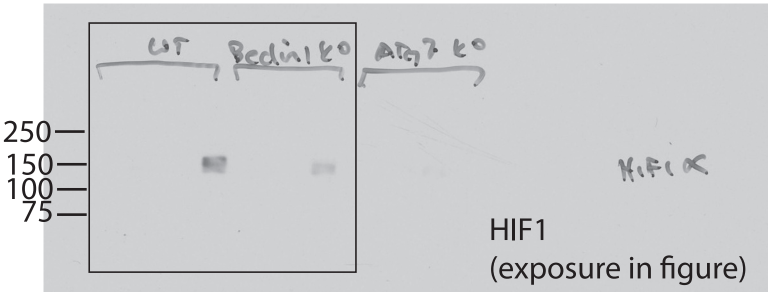

HIF1
(exposure in figure)

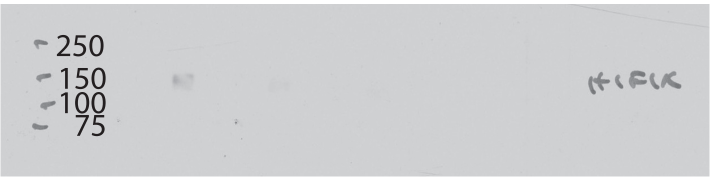

HIF1 (exposure with ladder)

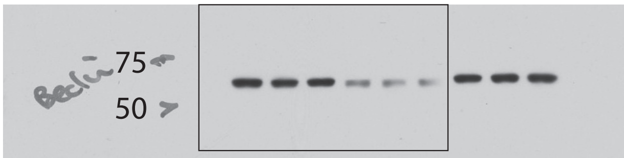

Beclin1

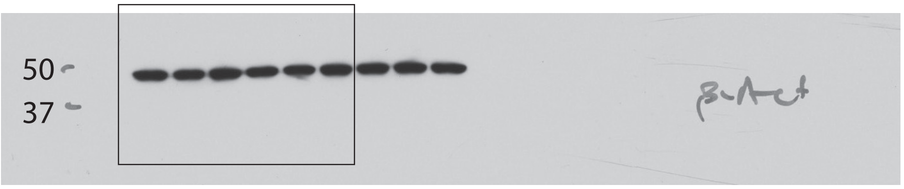

B-Actin

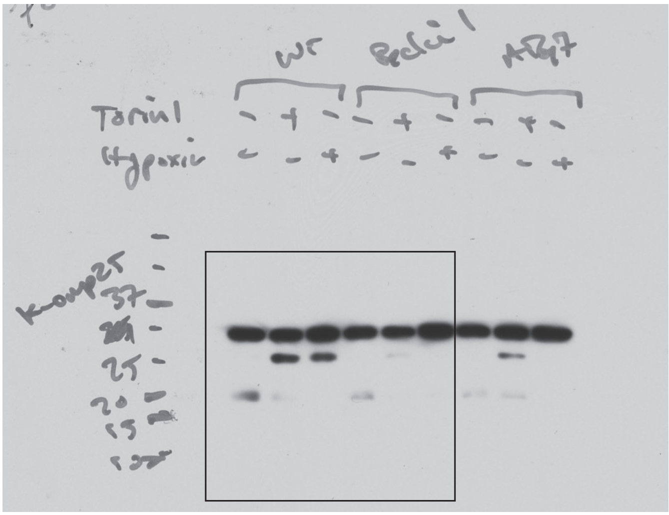

Keima

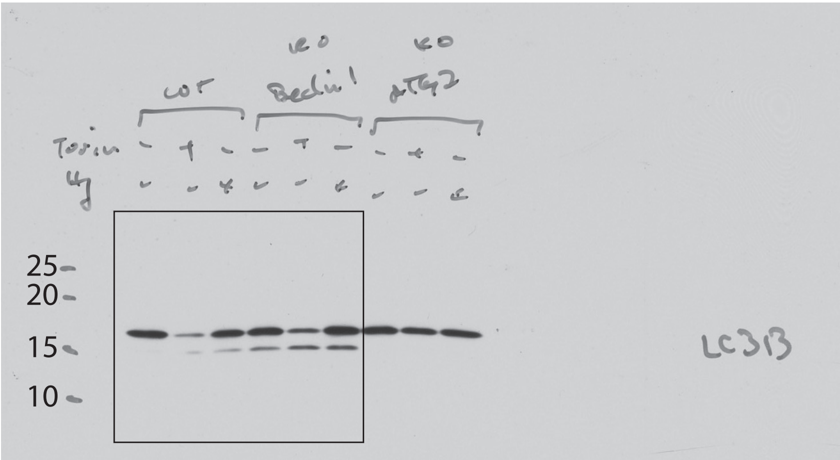

LC3B

Full unedited gel for Figure 1G

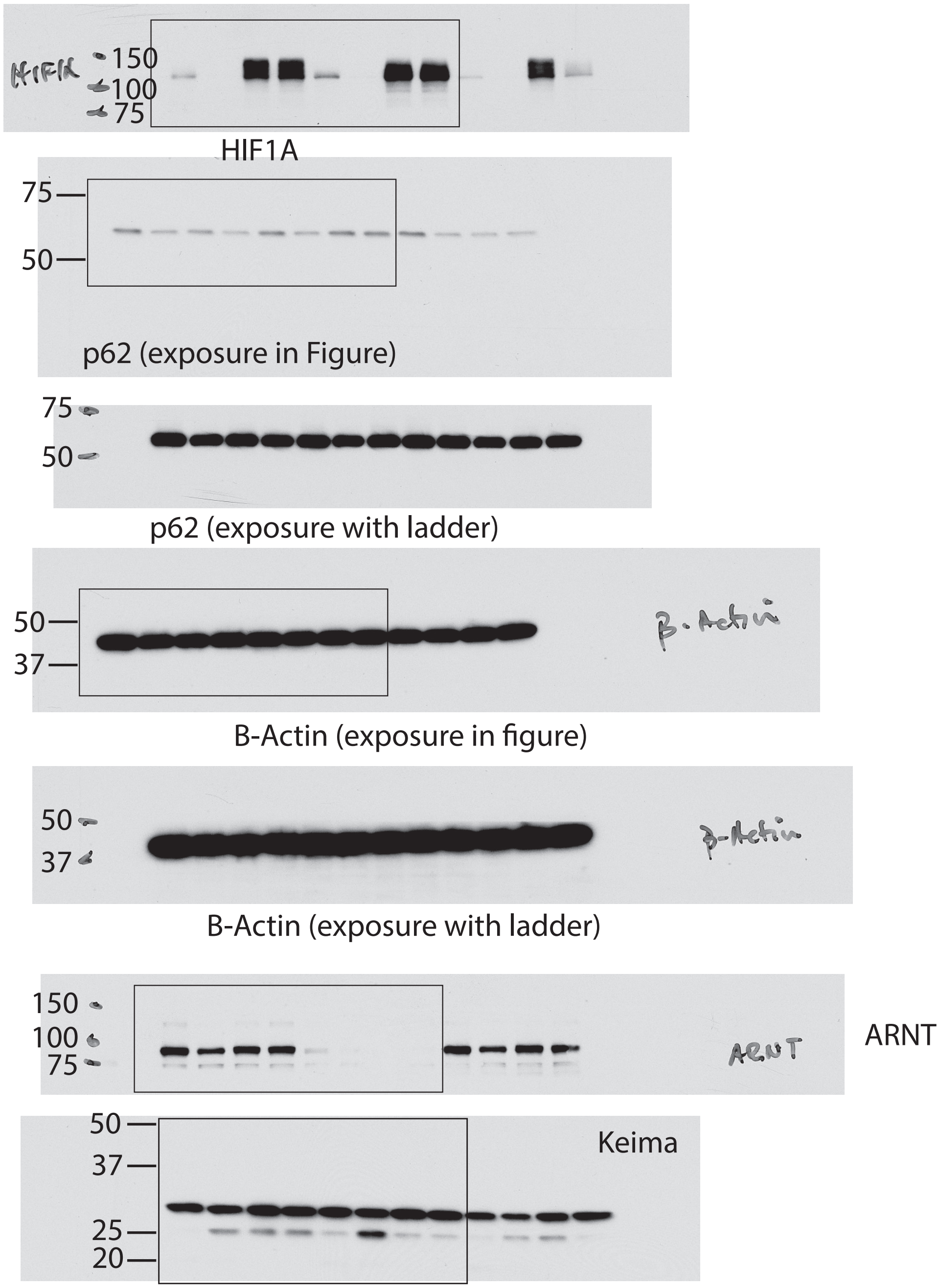

Full unedited gel for Figure 2D

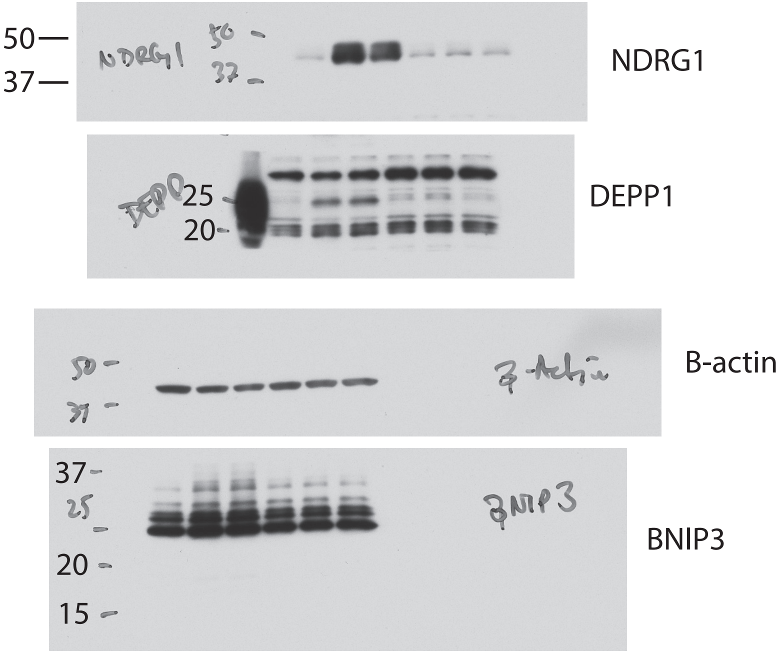

Full unedited gel for Figure 2G

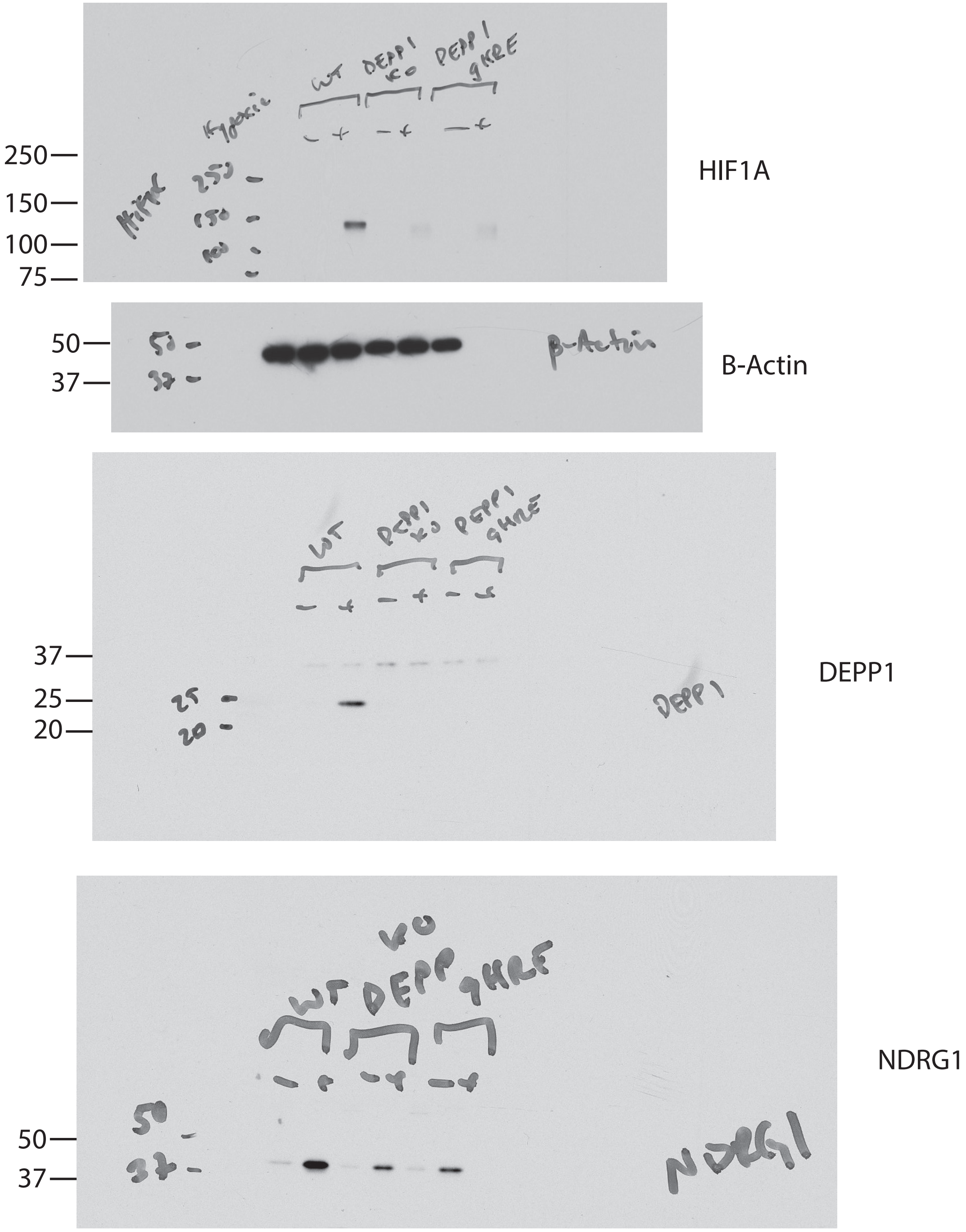

Full unedited gel for Figure 4C

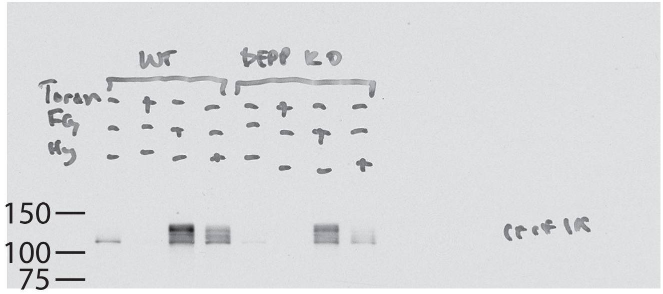

HIF1A

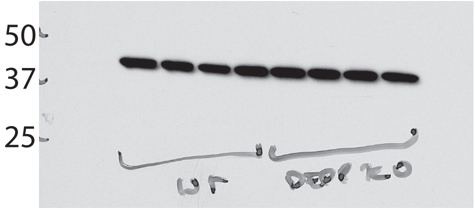

B-Actin

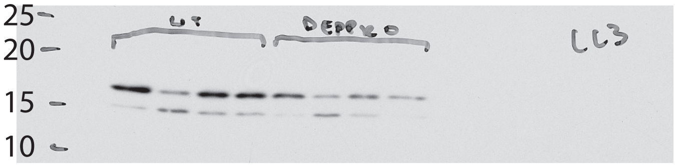

LC3B

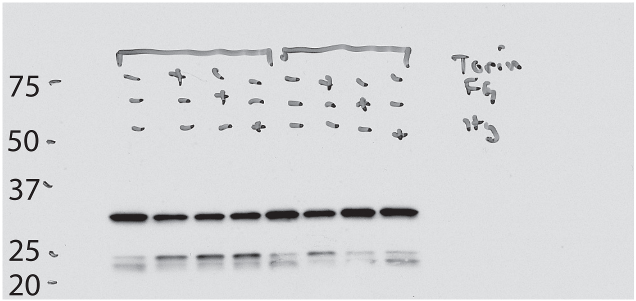

Keima

Full unedited gel for Figure 4D

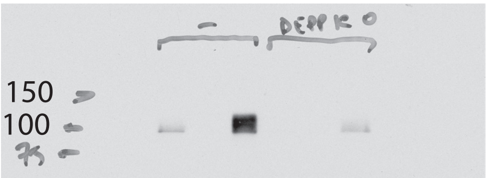

HIF1A

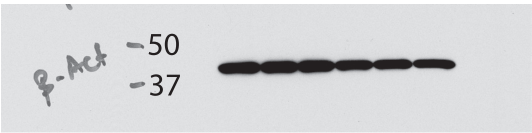

B-Actin

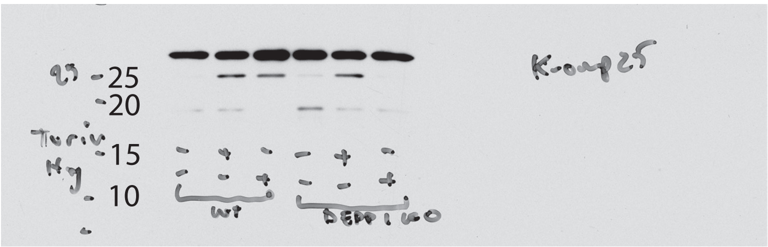

Keima

Full Unedited Gel for FIgure 4E

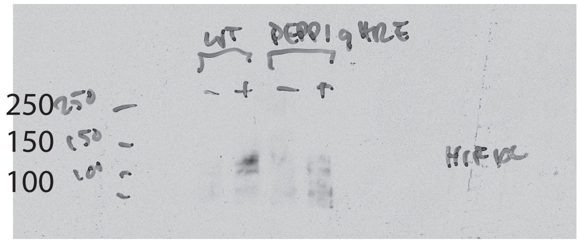

HIF1A

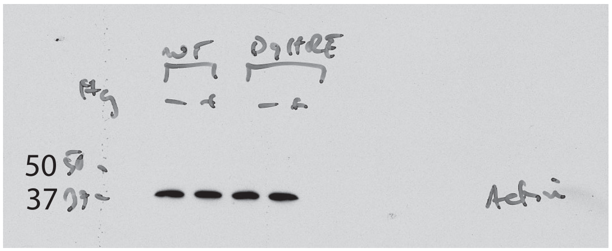

B-Actin

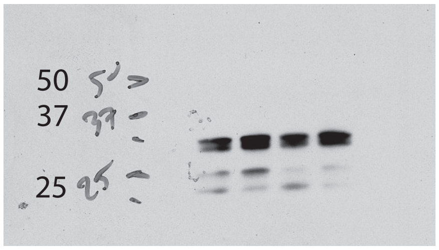

Keima

Full Unedited Gel for Figure 4F

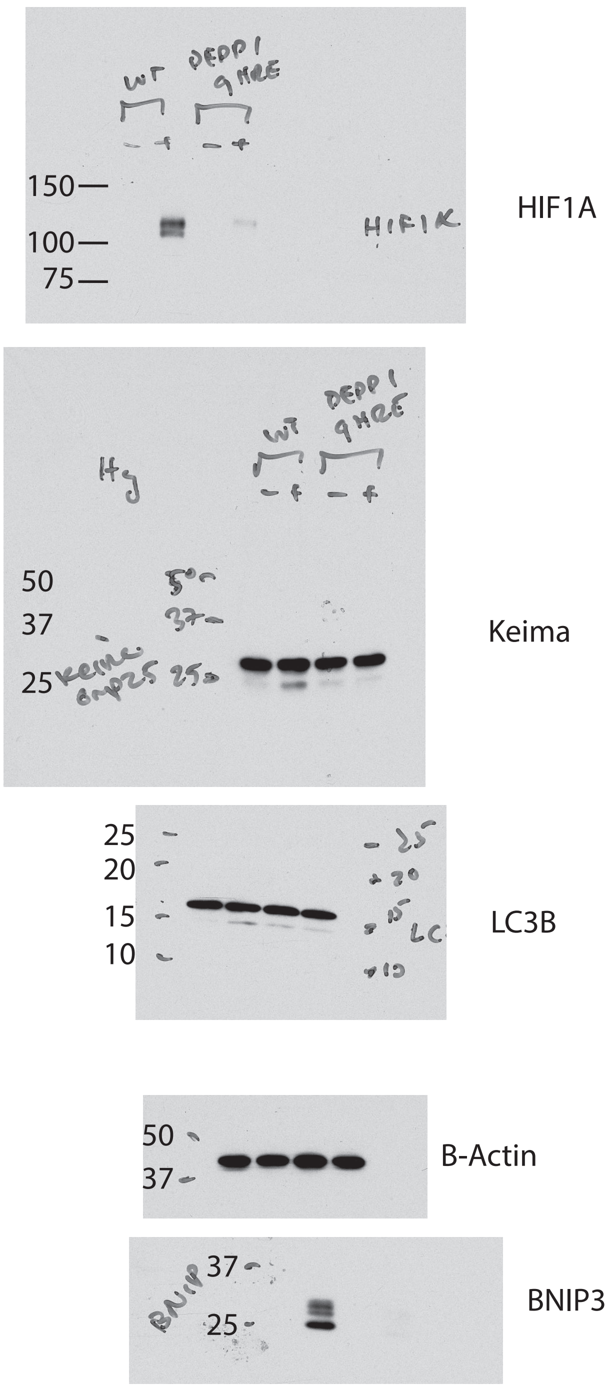

Full Unedited Gel for Figure 5A

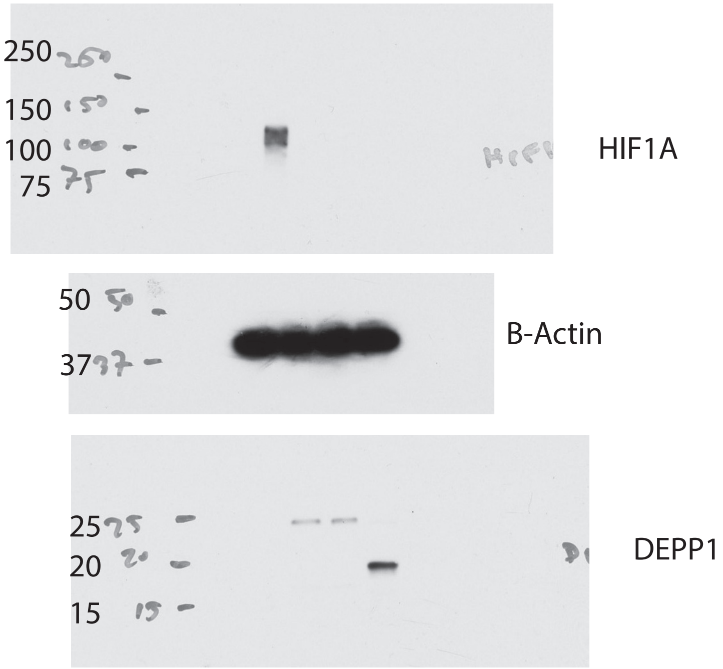

Full Unedited Gel for Figure 6A

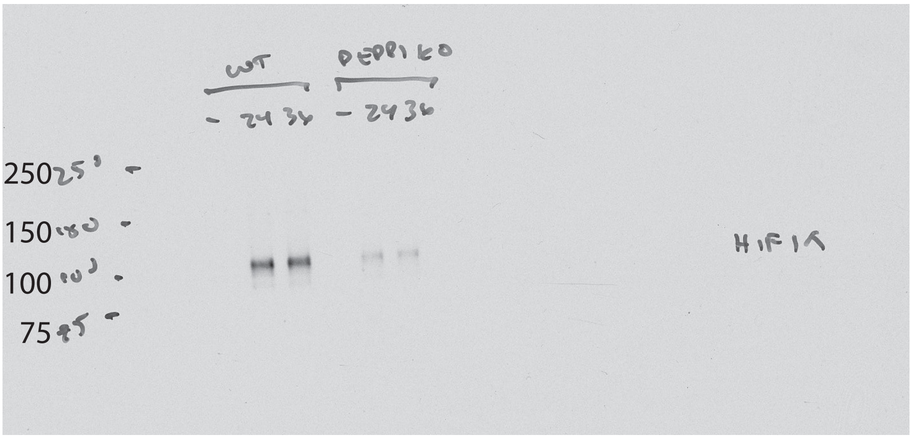

HIF1A

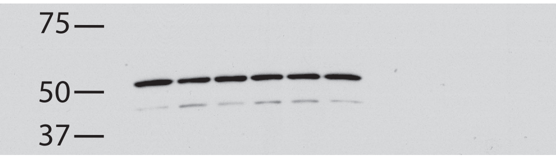

calreticulin

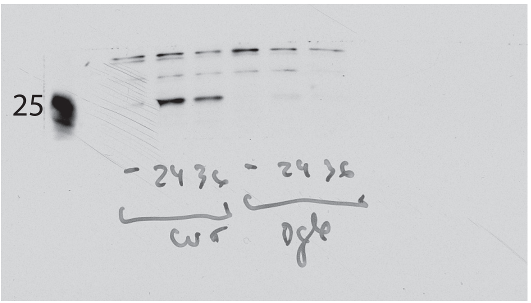

DEPP1

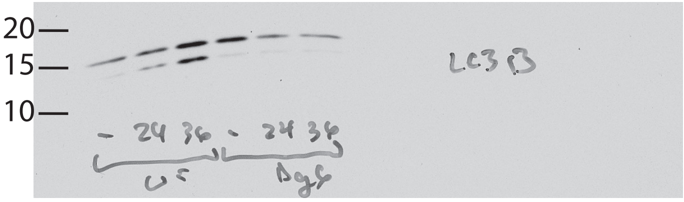

LC3B

Full Unedited Gel for Figure 6B

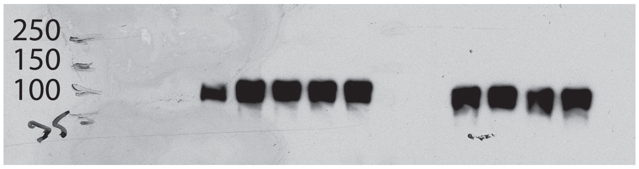

HIF1A

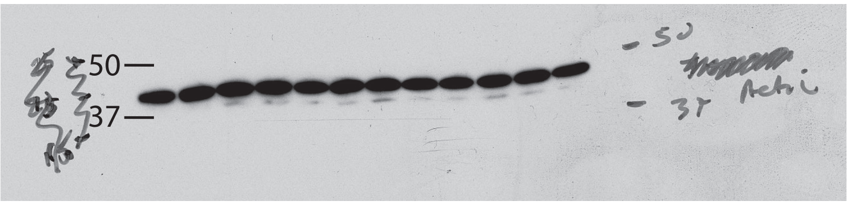

B-Actin

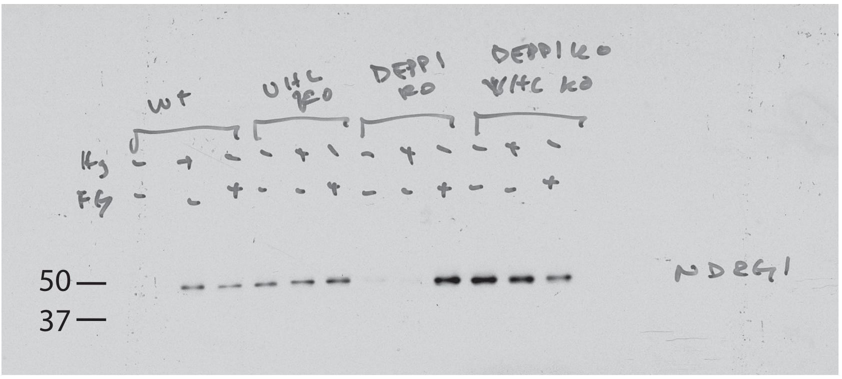

NDRG1

Full Unedited Gel for Figure 6C

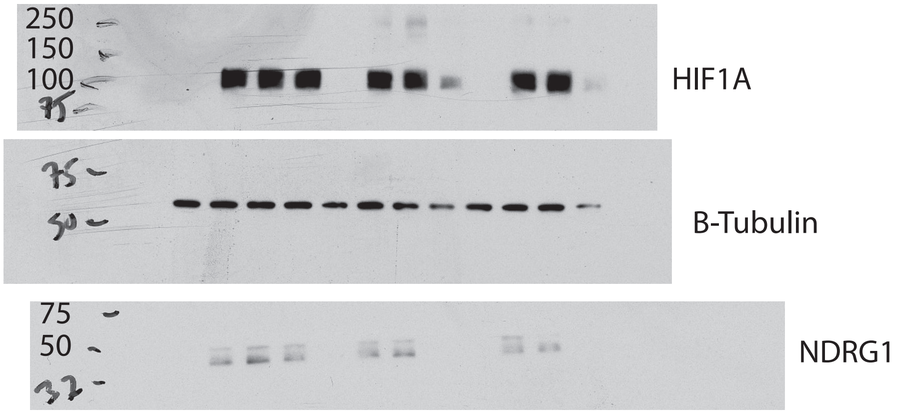

Full Unedited Gel for Figure 6E

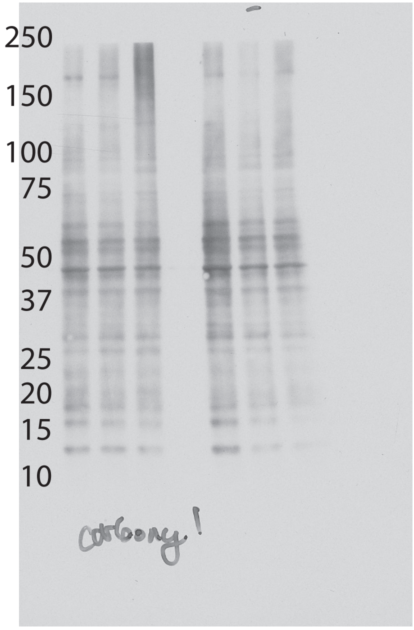

Carbonyl

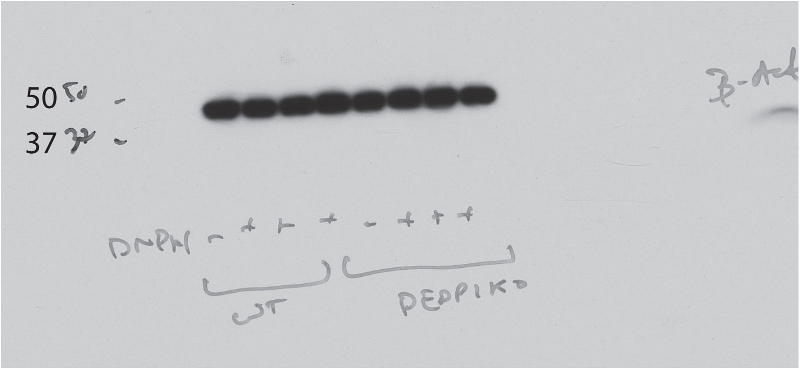

B-Actin

Full Unedited Gel for Figure S1A

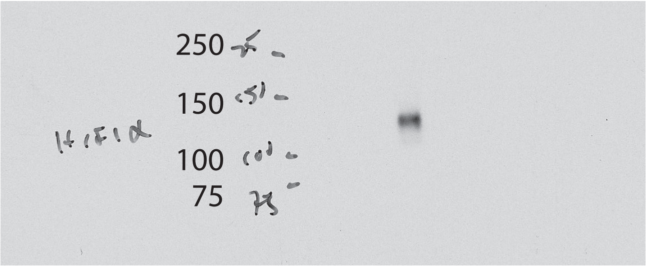

HIF1A

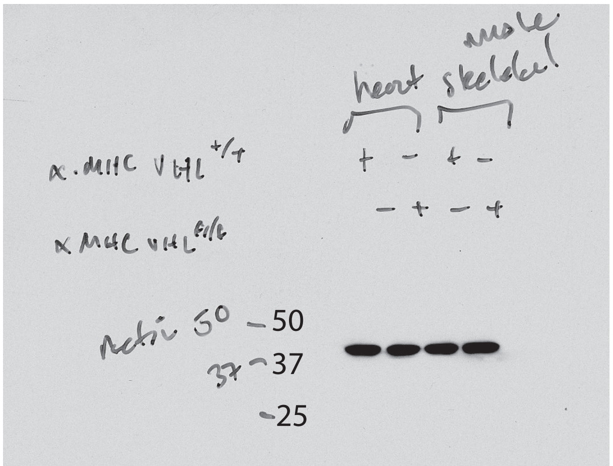

B-actin

Full Unedited Gel for Figure S5C

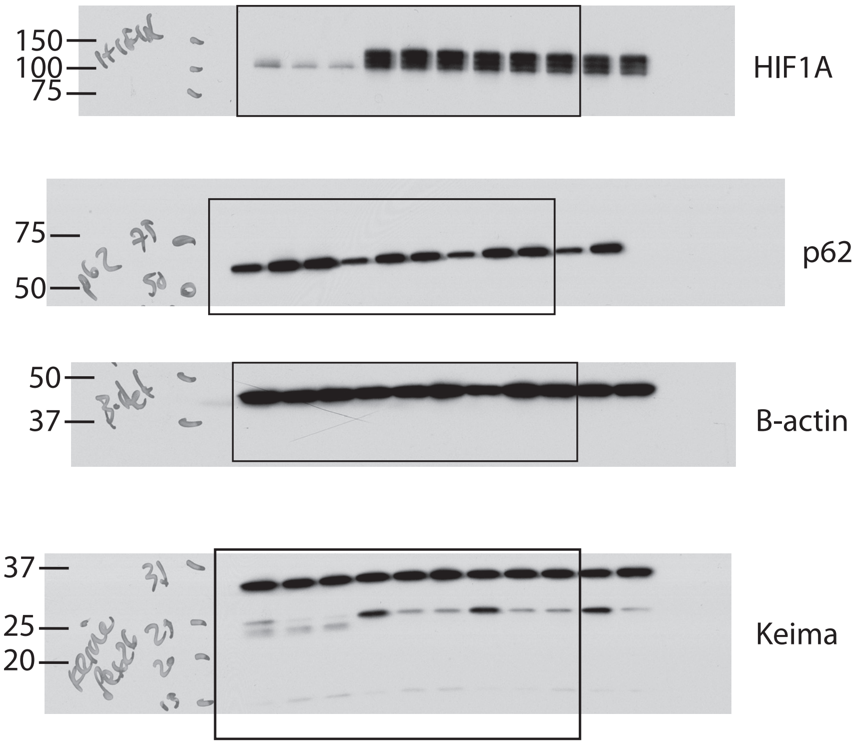

Full Unedited Gel for Figure S5D

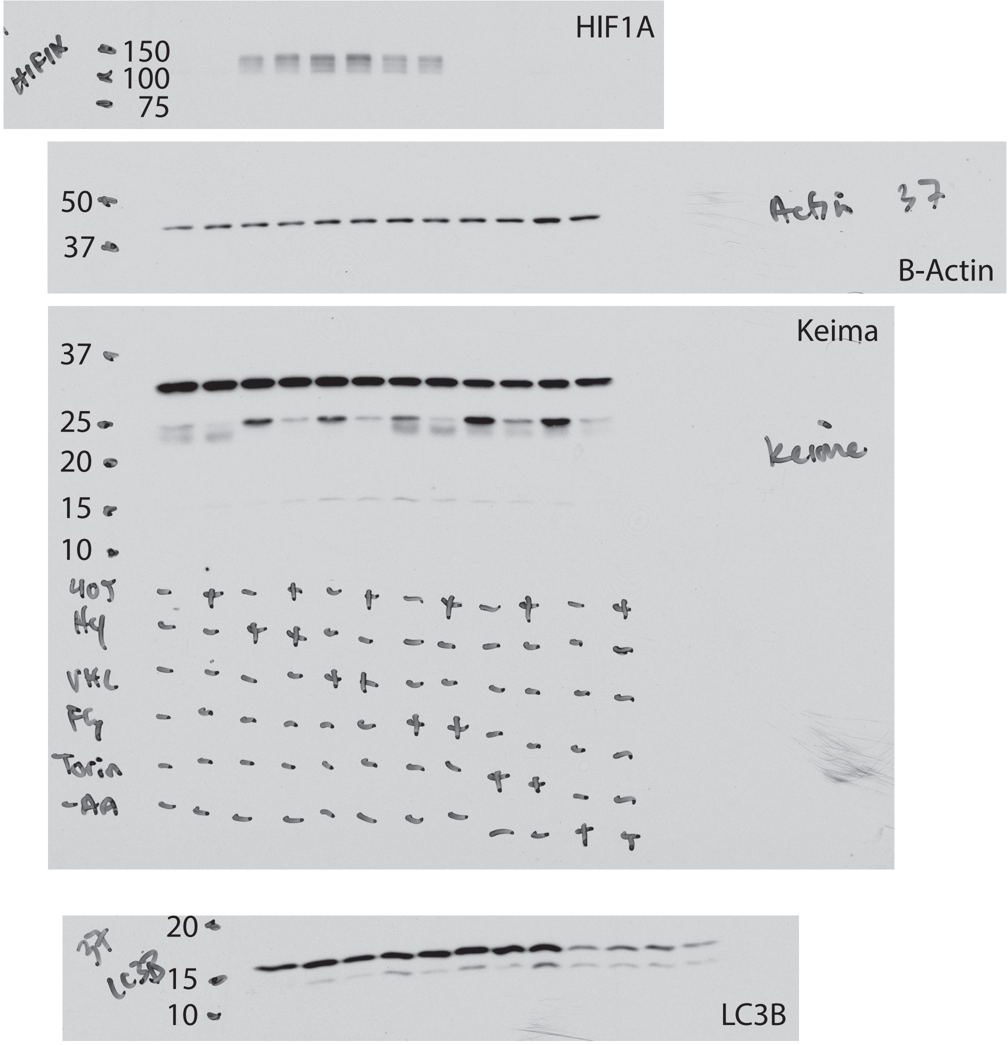

Full Unedited Gel for Figure S5E

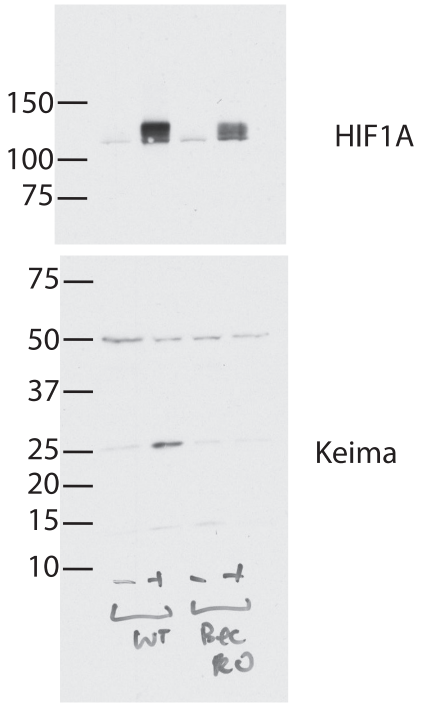

Full Unedited Gel for Figure S5F

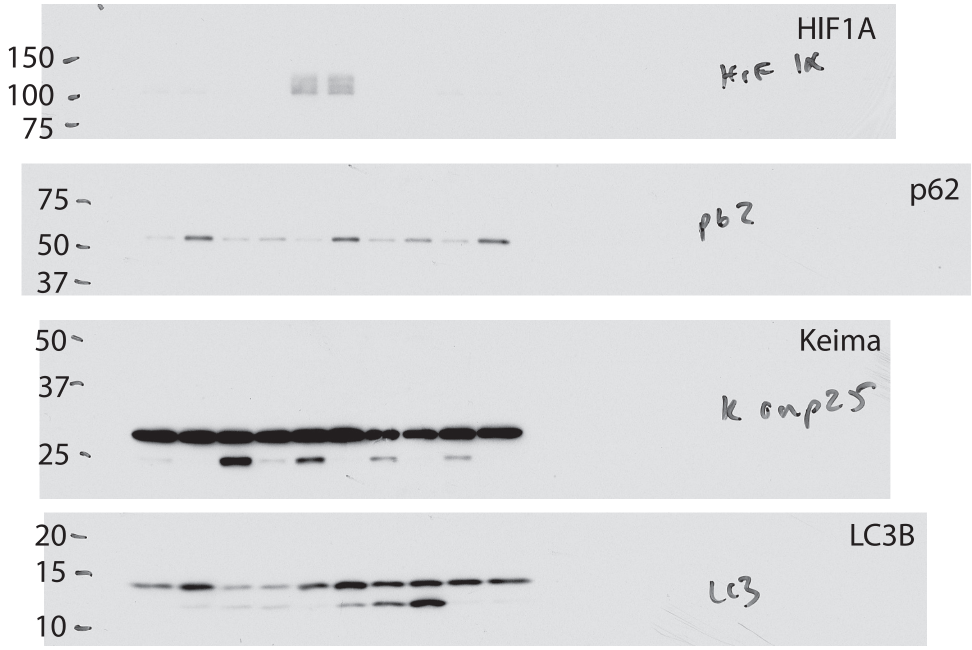

Full Unedited Gel for Figure S7D

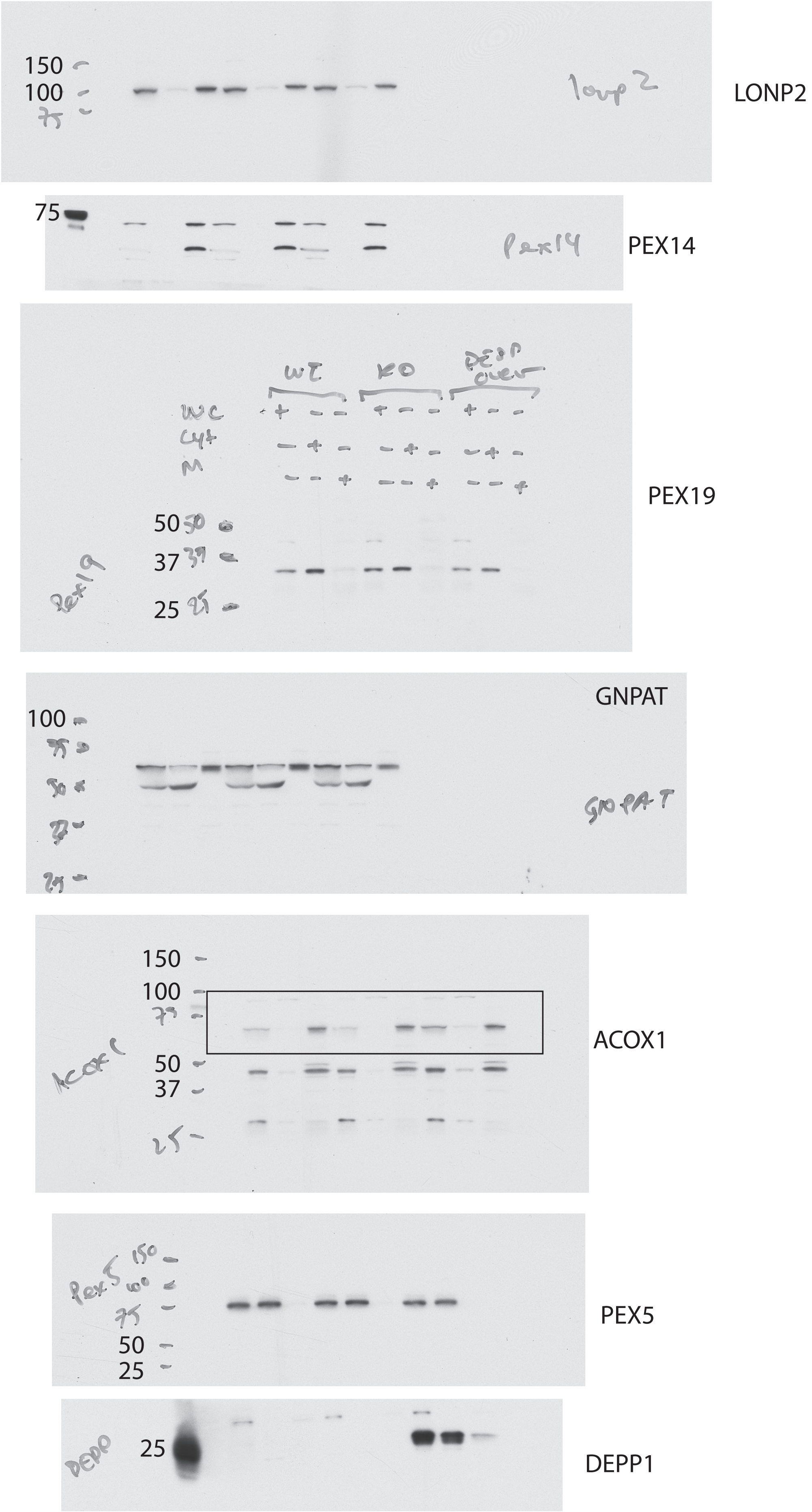

Full Unedited Gel for Figure S8B

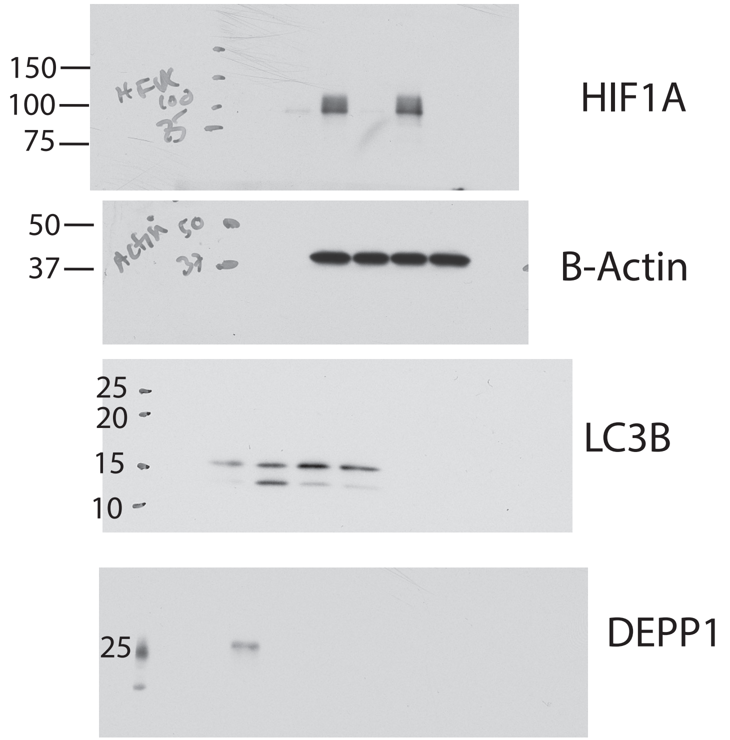

Full Unedited Gel for Figure S8C

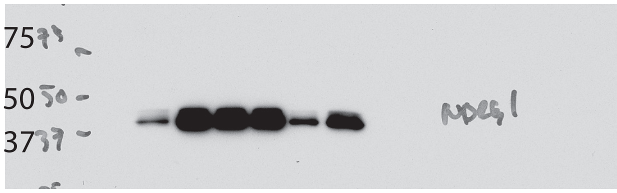

NDRG1

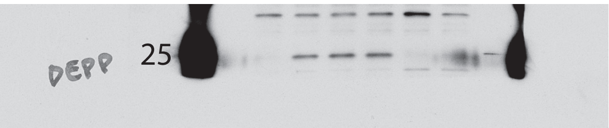

DEPP1

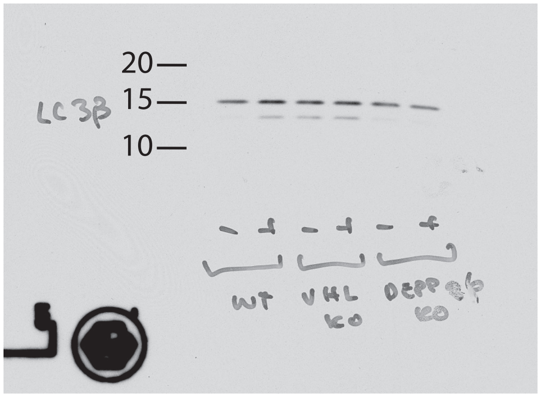

LC3B

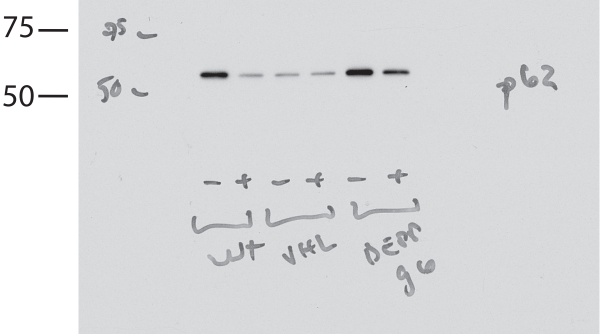

p62

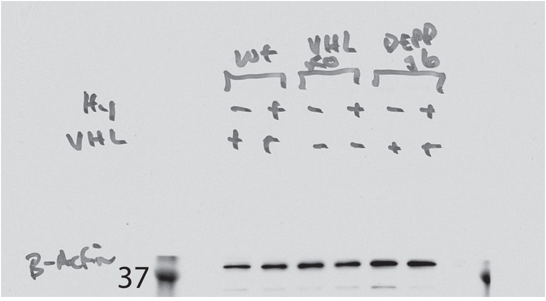

B-Actin

Full Unedited Gel for Figure S8D

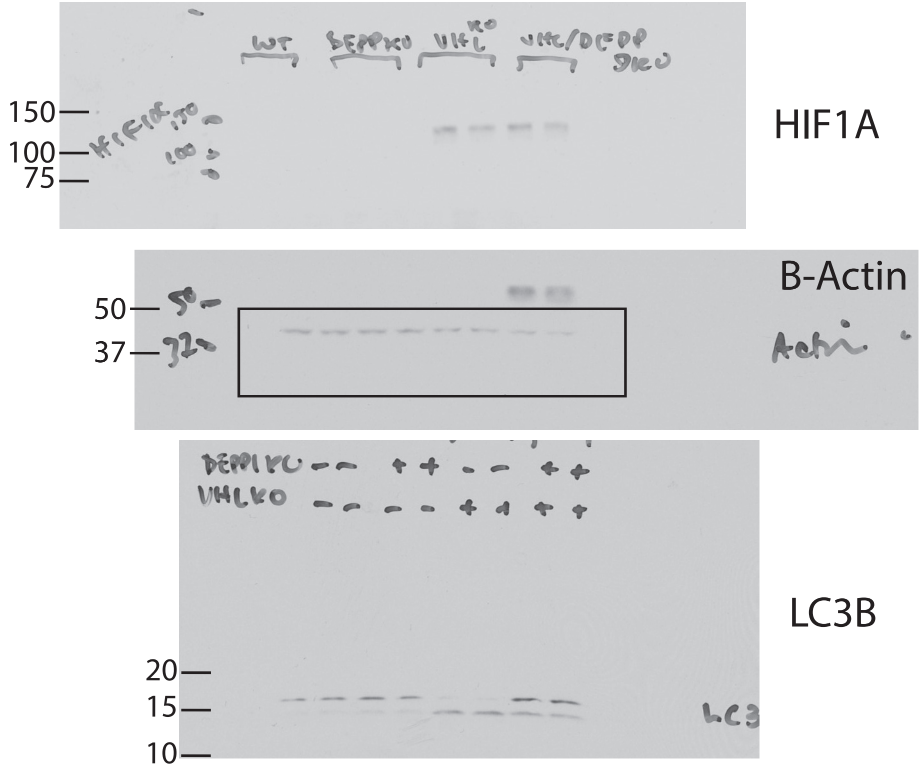

Full Unedited Gel for Figure S11A

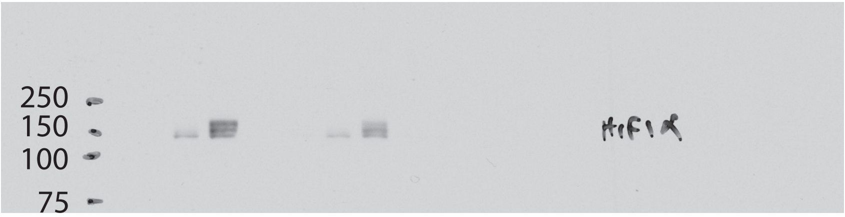

HIF1A

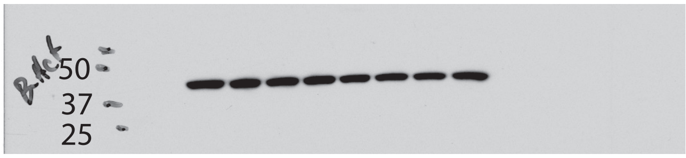

B-Actin

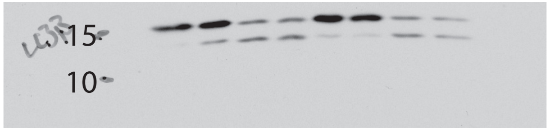

LC3B

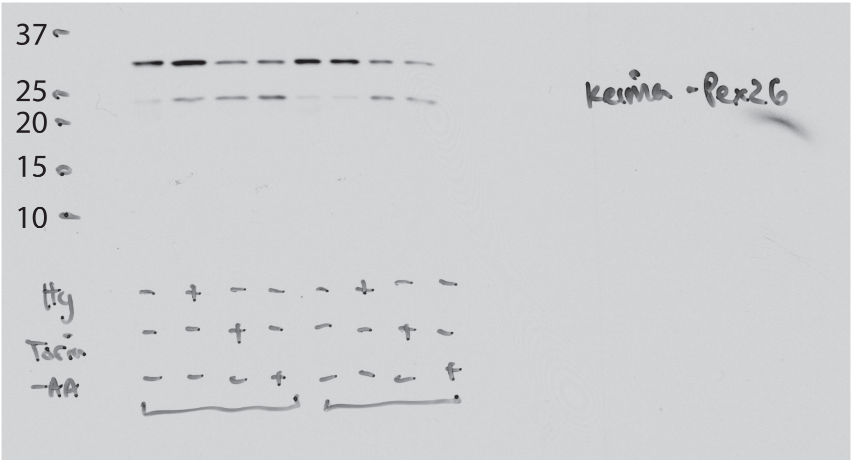

Keima

Full Unedited Gel for Figure S11B

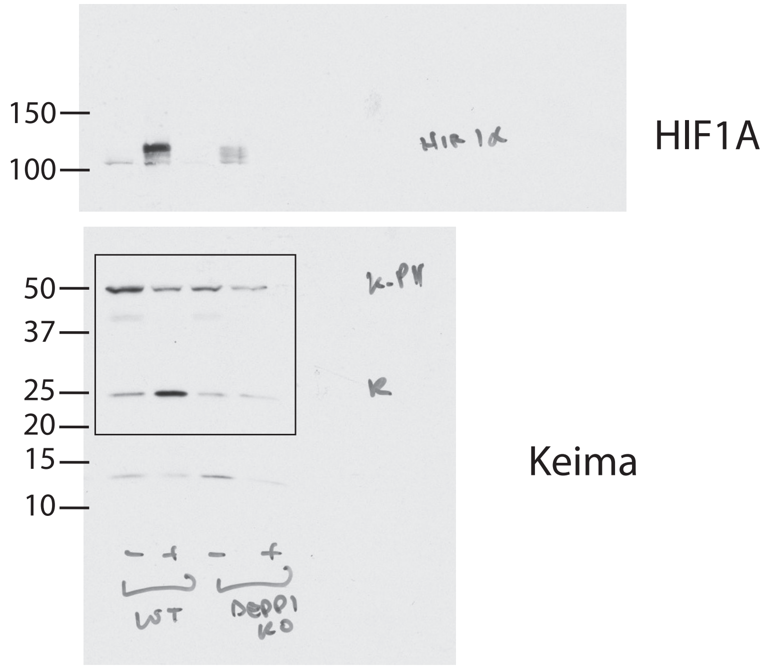

Full Unedited Gel for Figure S11C

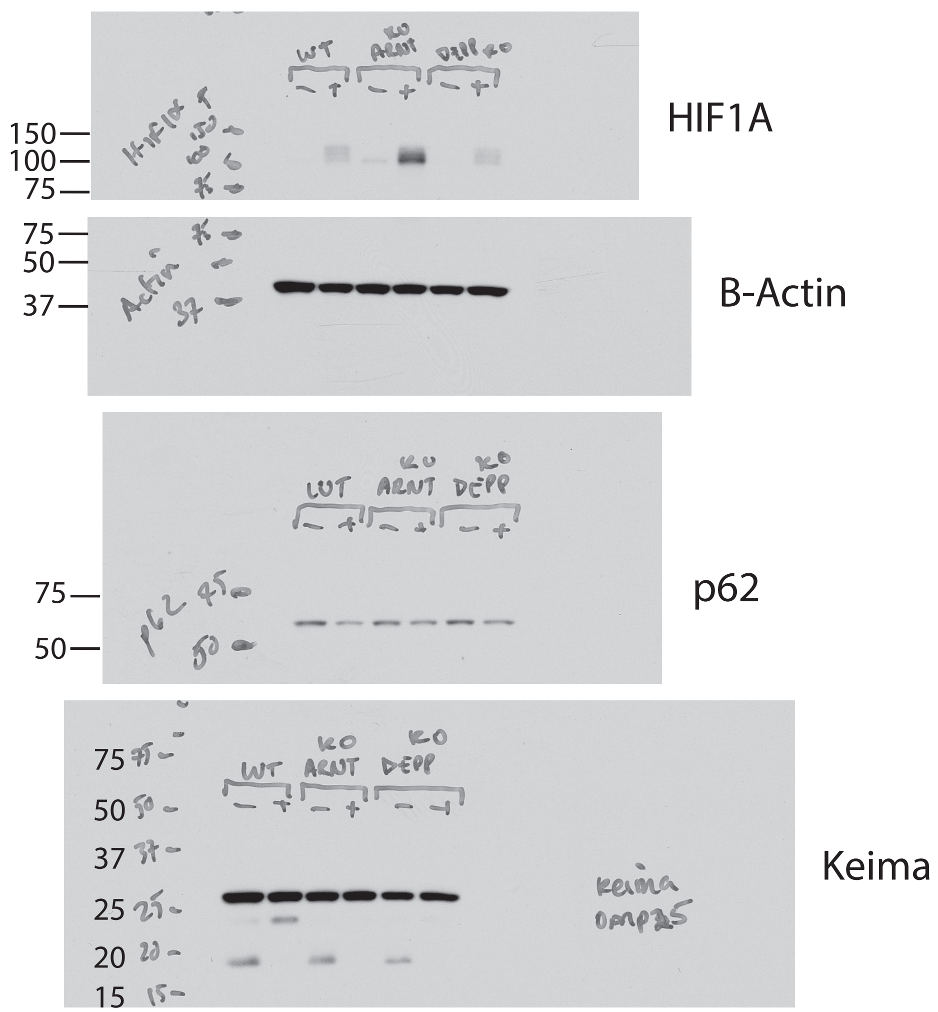

Full Unedited Gel for Figure S16A

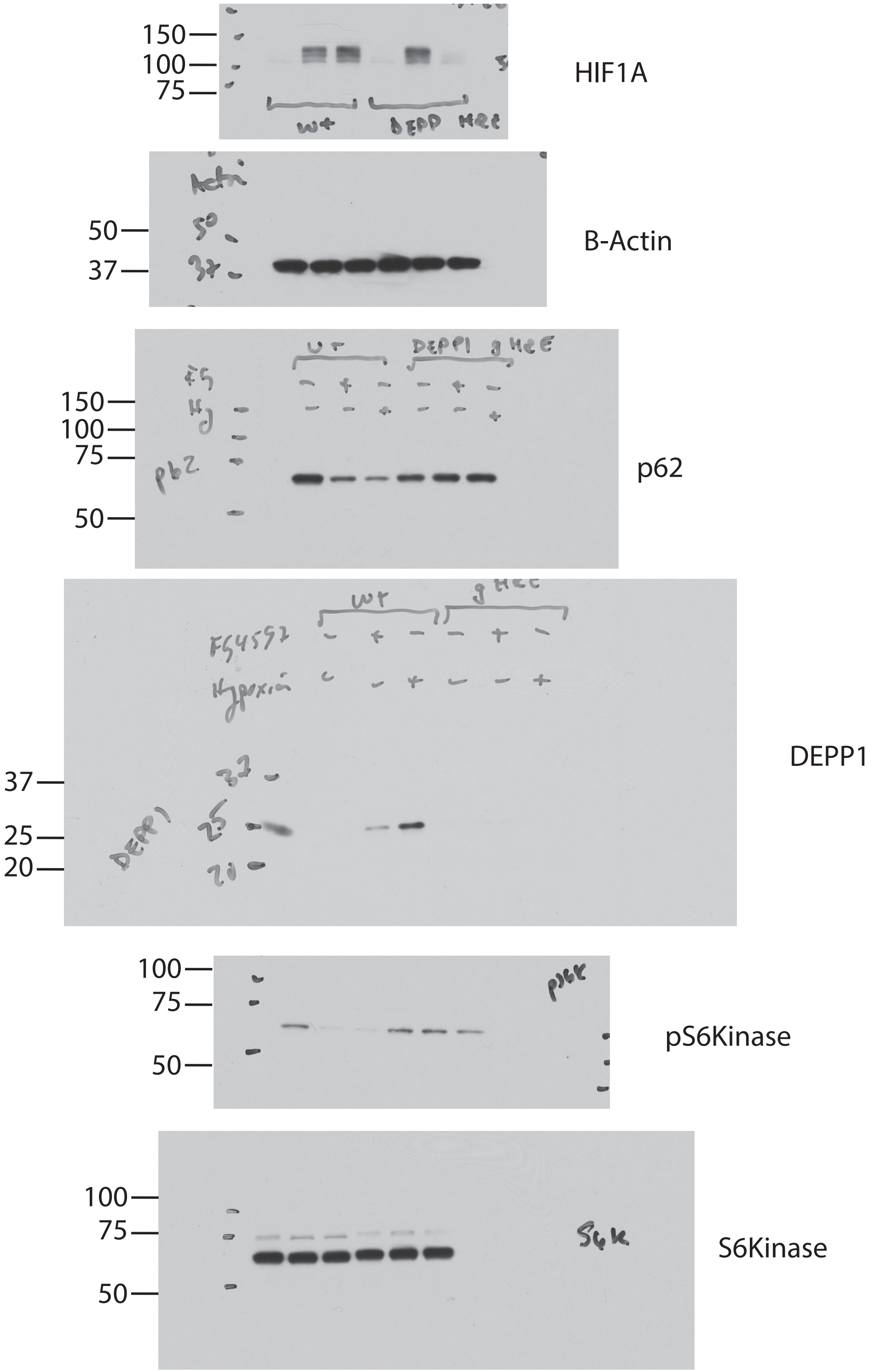

Full Unedited Gel for Figure S17B

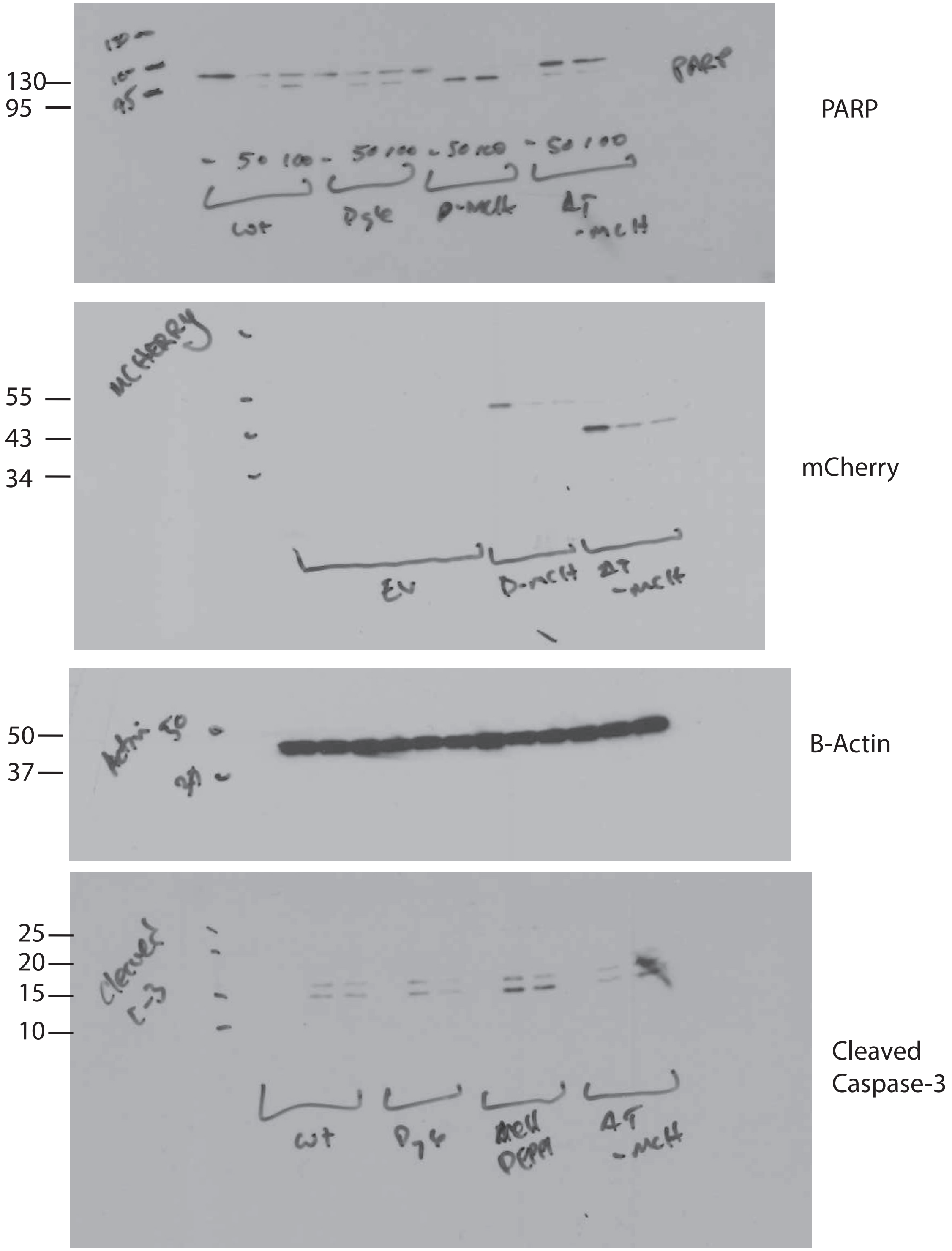

Full Unedited Gel for Figure S18A

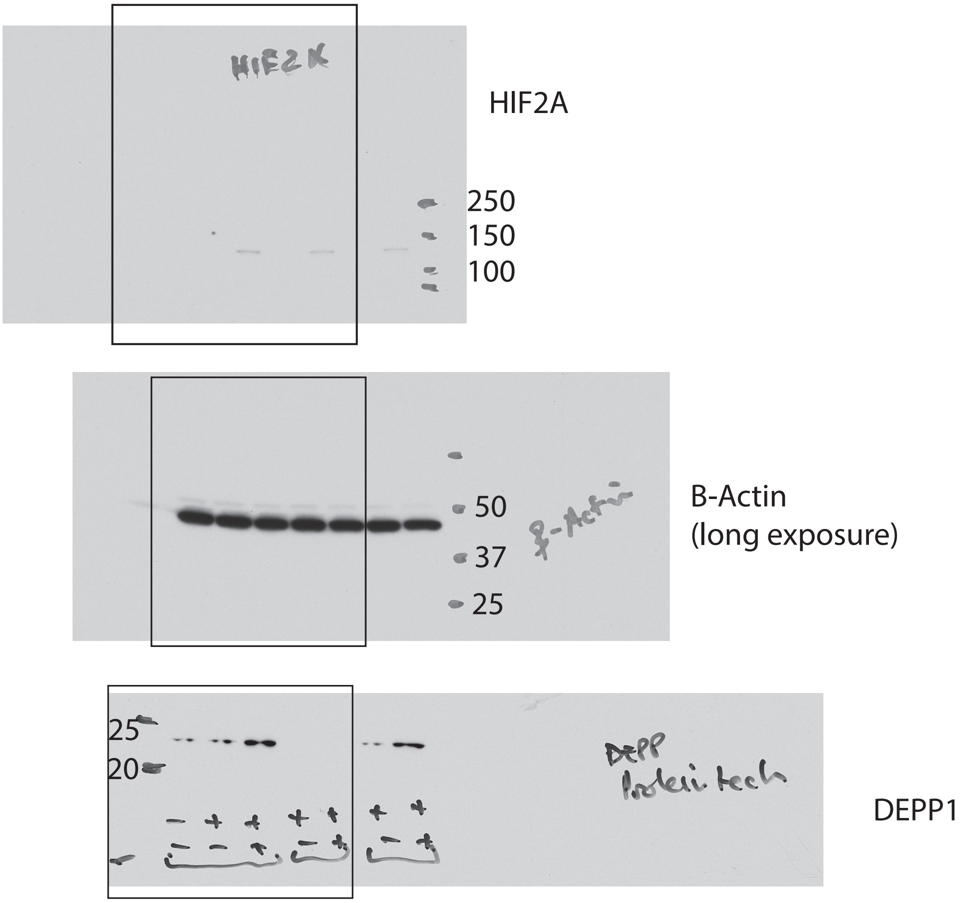

Full Unedited Gel for Figure S19A

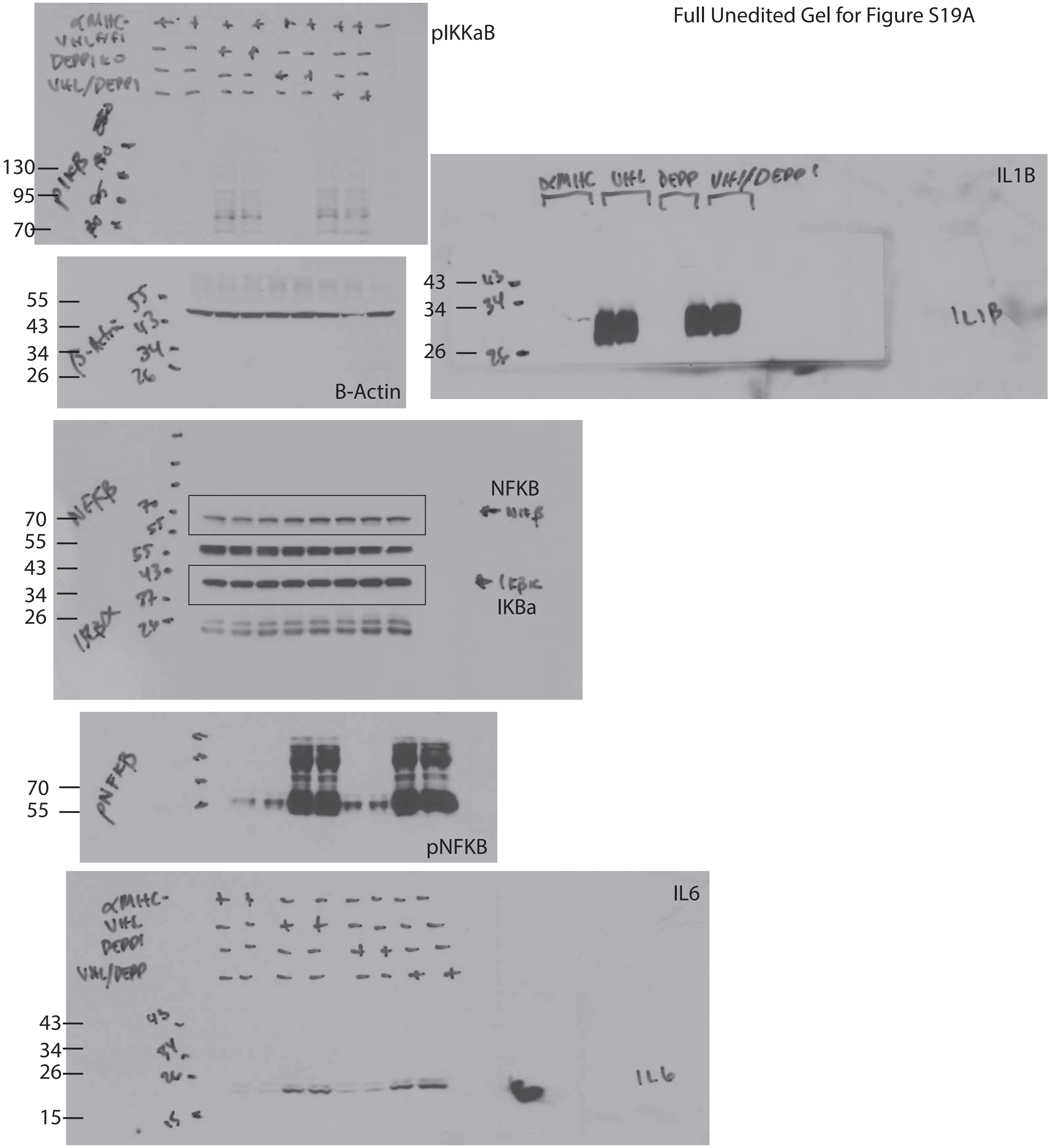

Full Unedited Gel for Figure S20A

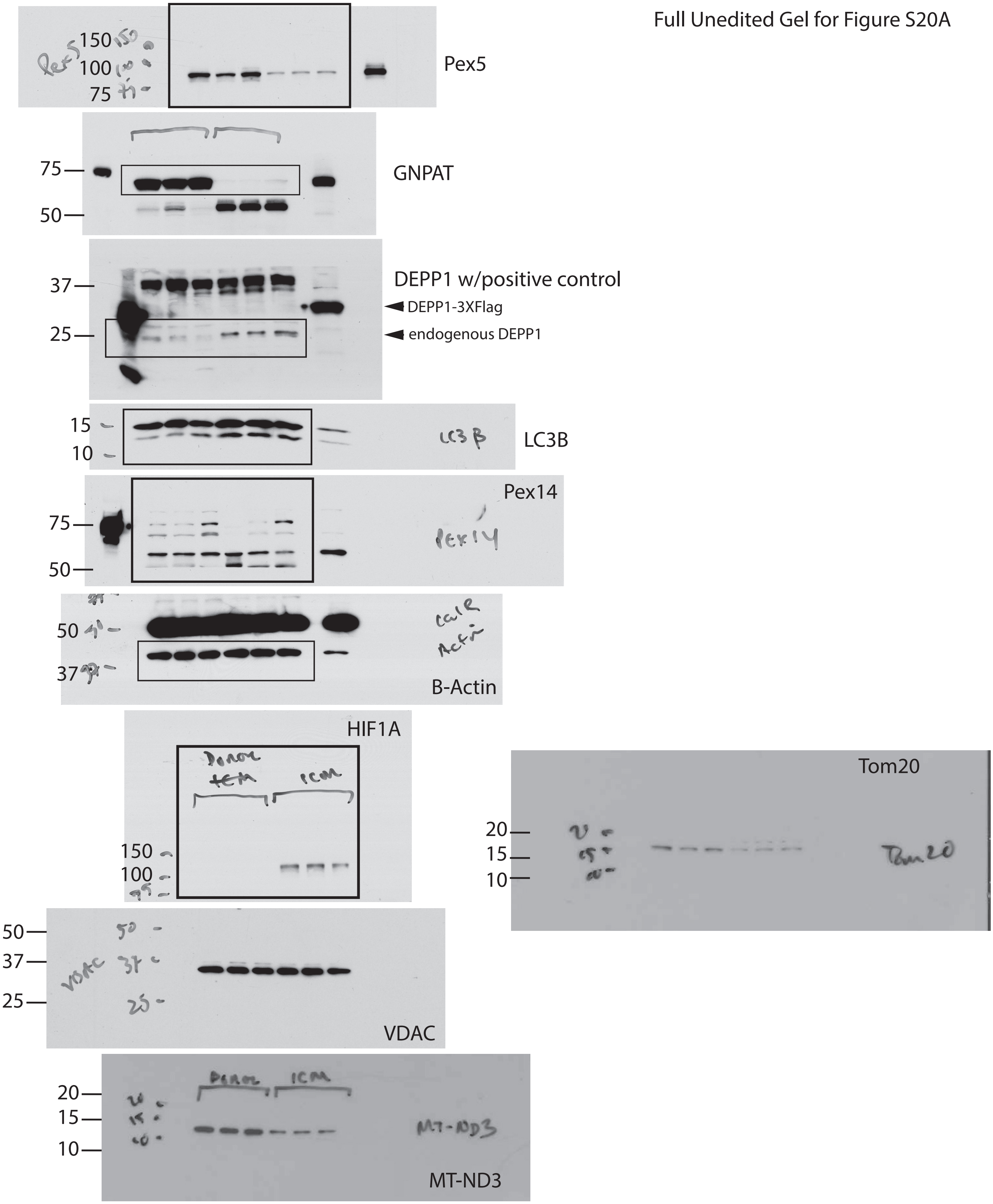

Supplement: Supplementary file 2 [file cir-150-770-s002.pdf]
